# Constructing Built-In Electric Fields with Semiconductor Junctions and Schottky Junctions Based on Mo–MXene/Mo–Metal Sulfides for Electromagnetic Response

**DOI:** 10.1007/s40820-024-01449-7

**Published:** 2024-06-11

**Authors:** Xiaojun Zeng, Xiao Jiang, Ya Ning, Yanfeng Gao, Renchao Che

**Affiliations:** 1 School of Materials Science and Engineering, Jingdezhen Ceramic University, Jingdezhen, 333403 People’s Republic of China; 2 School of Materials Science and Engineering, Shanghai University, Shanghai, 200444 People’s Republic of China; 3 Laboratory of Advanced Materials, Shanghai Key Lab of Molecular Catalysis and Innovative Materials, Academy for Engineering and Technology, Fudan University, Shanghai, 200438 People’s Republic of China; 4 Zhejiang Laboratory, Hangzhou, 311100 People’s Republic of China

**Keywords:** Semiconductor–semiconductor–metal heterostructure, Semiconductor junctions, Mott–Schottky junctions, Built-in electric field, Electromagnetic wave absorption

## Abstract

The exploration of novel multivariate heterostructures has emerged as a pivotal strategy for developing high-performance electromagnetic wave (EMW) absorption materials. However, the loss mechanism in traditional heterostructures is relatively simple, guided by empirical observations, and is not monotonous. In this work, we presented a novel semiconductor–semiconductor–metal heterostructure system, Mo–MXene/Mo–metal sulfides (metal = Sn, Fe, Mn, Co, Ni, Zn, and Cu), including semiconductor junctions and Mott–Schottky junctions. By skillfully combining these distinct functional components (Mo–MXene, MoS_2_, metal sulfides), we can engineer a multiple heterogeneous interface with superior absorption capabilities, broad effective absorption bandwidths, and ultrathin matching thickness. The successful establishment of semiconductor–semiconductor–metal heterostructures gives rise to a built-in electric field that intensifies electron transfer, as confirmed by density functional theory, which collaborates with multiple dielectric polarization mechanisms to substantially amplify EMW absorption. We detailed a successful synthesis of a series of Mo–MXene/Mo–metal sulfides featuring both semiconductor–semiconductor and semiconductor–metal interfaces. The achievements were most pronounced in Mo–MXene/Mo–Sn sulfide, which achieved remarkable reflection loss values of − 70.6 dB at a matching thickness of only 1.885 mm. Radar cross-section calculations indicate that these MXene/Mo–metal sulfides have tremendous potential in practical military stealth technology. This work marks a departure from conventional component design limitations and presents a novel pathway for the creation of advanced MXene-based composites with potent EMW absorption capabilities.

**Supplementary Information:**

The online version contains supplementary material available at 10.1007/s40820-024-01449-7.

## Highlights

- Mo–MXene/Mo–metal sulfides with semiconductor junctions and Mott–Schottky junctions are designed.
- Built-in electric field are constructed in semiconductor–semiconductor–metal heterostructure, enhancing dielectric polarization and impedance matching.
- Density functional theory calculations and Radar cross-section simulations confirmed the excellent electromagnetic wave absorption ability of heterostructures.

**Supplementary Information:**

The online version contains supplementary material available at 10.1007/s40820-024-01449-7.

## Graphical abstract

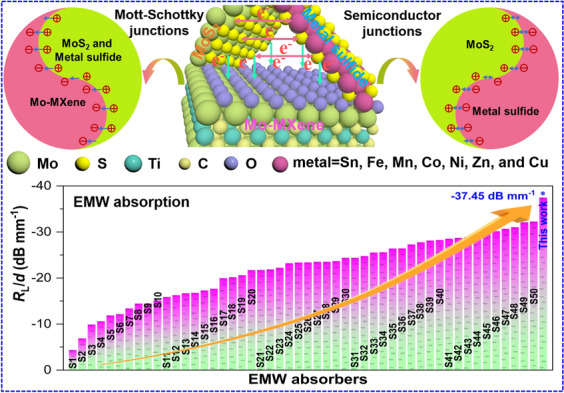

## Introduction

In light of the rapid advancements in electronic technology in the domains of 5G communication and military applications, there has been a significant development of electromagnetic wave (EMW) absorption materials, which play a pivotal role as responsive materials in the realm of EMW technology [1–4]. Furthermore, in response to escalating concerns about electromagnetic pollution, heightened attention has been directed toward efficient EMW absorption materials, such as carbon-based substances, metal compounds, metal–organic framework (MOF) derivatives, and MXene in recent times [5–8]. These absorption materials have been crafted with a keen focus on fulfilling distinct EMW absorption criteria. These criteria encompass diverse facets including robust absorption capabilities, adaptability to harsh environments, and broad effective absorption bandwidths (EAB) [9]. However, the pursuit of a single material capable of satisfying all these requisites while simultaneously achieving excellent impedance matching remains a formidable challenge [10–12]. As a result, there exists an imperative to synergistically integrate diverse materials with complementary properties [13]. This strategic amalgamation not only addresses specific EMW absorption mandates, but also leverages the synergy of different components to create a unique and effective solution.

Two-dimensional (2D) materials often have unique electronic states, such as quantum-limited-domain effects, attenuated Coulomb shielding effects, etc. As an example of 2D transition metal sulfides (TMDs), MoS_2_ possesses a trilayer structure consisting of S–Mo–S layers. This distinctive architecture, characterized by a trilayer stacked atomic configuration connected via van der Waals forces, facilitates rapid charge transfer. MoS_2_ has emerged as a notable contender in the EMW absorption field, attributed to its ease of fabrication, cost-effectiveness, and semiconductor attributes [14]. However, MoS_2_ absorbers inherent challenges related to inadequate electrical conductivity and lack of edge sites, which resulted in suboptimal EMW absorption performance [15]. It has been discerned that adding other materials such as graphene, MXene, and semiconductor materials to MoS_2_ can enhance EMW absorption to some degree [16, 17]. Furthermore, the creation of a heterojunction between two semiconductors is also a promising avenue to improve absorption capacity [14]. Similarly, metal sulfides (metal = Sn, Fe, Mn, Co, Ni, Zn, and Cu) have received great attention as a class of semiconductor that can be used to construct heterojunctions. This is mainly because metal sulfides typically have narrow band gaps (~ 2 eV) and exhibit good EMW absorption properties when combined with other semiconductors [18–20]. Generally, a larger band gap makes it more difficult for electrons to be excited from the valence band to the conduction band, resulting in lower electrical conductivity. For example, Xing and co-workers created 3D layered MoS_2_/FeS_2_ composites with heterojunctions through a straightforward hydrothermal process. This heterojunction leads to close ohmic contacts and generates a local electric field, resulting in substantial enhancements in absorption performance [21]. Based on the insights garnered from these precedents, we contemplate the establishment of semiconductor heterojunctions by incorporating metal sulfides into the MoS_2_ matrix. This strategic integration aims to engender a built-in electric field (BIEF) at the interface, thereby enhancing the EMW absorption capacity of semiconductor material.

As a novel 2D material, MXene has garnered substantial attention in terms of EMW shielding and EMW absorption [22–25]. Notably, MXene boasts metallic conductivity and a heightened density of electronic states proximate to the Fermi energy level, rendering it a promising electron donor that can significantly contribute to the realization of BIEF at the interface [26]. For instance, Chen et al. demonstrated the creation of SnS_2_-MXene Mott–Schottky heterostructures, featuring interfacial BIEF, by one-step hydrothermal growth of SnS_2_ on the MXene surface [27]. Choi et al. achieved the construction of semiconductor/metal Schottky junctions by converting the edges of Ti_3_C_2_ MXene into semiconductor TiO_2_ material [28]. These findings emphasize that MXene with metallic properties can be used as a key element in the fabrication of Mott–Schottky heterojunctions, working with semiconductor materials to build multidimensional heterojunctions and generate interfacial BIEFs.

The concept of BIEF was initially proposed by the physics community in 1999, and has since been applied in fields such as energy storage, conversion, electrocatalysis, and zinc-air batteries [29]. Essentially, the creation of BIEF is contingent upon the charge redistribution stemming from the spontaneous flow of electrons at the interface. This can be realized by contacting two semiconductors with distinct work functions or by forming a Mott–Schottky heterojunction through the interaction of metal and semiconductor [29]. However, researchers have identified inherent challenges associated with BIEF generated through semiconductor contacts. For instance, at the interface of semiconductor heterojunctions, a rapid recombination of accumulated electrons and holes often transpires within a short interval, thus affecting the establishment of interfacial BIEF [30, 31]. Fortunately, investigations have shown that Mott–Schottky heterojunctions established through interactions between metals and semiconductors can produce more robust interfacial BIEF and significantly enhance electron transfer [32]. By heterojunction engineering, the synergistic coupling effect between different interfaces can be exploited to improve the electronic states of 2D materials and obtain distinctive properties. Therefore, it is possible to design a multidimensional heterojunction with enhanced EMW absorption properties by combining the advantages of semiconductor junctions and Mott–Schottky junctions. In addition, this unique heterojunction may lead to more complex electronic states than a single semiconductor junction and Mott–Schottky junction, forming multiple BIEF and modulating the electron transfer capability to bring about a suitable dielectric loss capability. Although some researchers have sought to enhance EMW absorption performance through the construction of semiconductor and Mott–Schottky heterojunctions, the literature scarcely addresses the amalgamation of both types of junctions to heighten EMW absorption efficacy in ongoing research efforts.

Guided by these considerations, we devised and synthesized a novel material characterized by a semiconductor–semiconductor–metal heterostructure. This intricate architecture contains synergistic heterojunctions composed of both semiconductor junctions and Mott–Schottky junctions. In this heterostructure, the artful combination of two distinct heterojunctions engenders a distinctive interfacial BIEF, orchestrating optimal electron transfer and appropriate impedance matching. This endeavor aimed to establish a universal approach that satisfies the requisites of both semiconductor and Mott–Schottky junctions by leveraging different semiconductor materials to form the corresponding heterojunctions. Consequently, we successfully engineered a series of Mo–MXene multi-heterostructures featuring semiconductor–semiconductor interfaces and semiconductor–metal interfaces through a facile hydrothermal approach. In this composite, MoS_2_ nanosheets and metal sulfides (metal = Sn, Fe, Mn, Co, Ni, Zn, and Cu) were grown atop the Mo–MXene substrate. Given the semiconducting nature of MoS_2_ and metal sulfides (metal = Sn, Fe, Mn, Co, Ni, Zn, and Cu), a semiconductor–semiconductor heterointerface with semiconductor junction properties arises between them. Moreover, a semiconductor–metal heterointerface characterized by Mott–Schottky characteristics emerges between Mo–MXene and the two semiconductor materials. The creation of these dual interfaces creates multiple BIEFs that accelerate fast electron transfer, thereby amplifying their dielectric loss capability. Meanwhile, the oscillating electromagnetic field prompts interface polarization, essentially bestowing a capacitor-like configuration that amplifies dielectric loss and enhances robust EMW absorption capabilities. Hence, the Mo–MXene/Mo–metal sulfide (metal = Sn, Fe, Mn, Co, Ni, Zn, and Cu) systems, featuring semiconductor–semiconductor–metal heterointerfaces, exhibit remarkable EMW absorption performance.

## Experimental

### Chemicals

Mo_2_TiAlC_2_ MAX (≥ 99%, 200 mesh) was purchased from Kai Xi Ceramic Materials Co., Ltd. Hydrofluoric acid (HF, 40 wt%), ammonium molybdate tetrahydrate ((NH_4_)_6_Mo_7_O_24_·4H_2_O, 99%), tin chloride pentahydrate (SnCl_4_·5H_2_O, 99%), iron chloride hexahydrate (FeCl_3_·6H_2_O, 99%), manganese acetate tetrahydrate (Mn(CH_3_COO)_2_·4H_2_O, 99%), cobalt nitrate hexahydrate (Co(NO_3_)_2_·6H_2_O, 99%), nickel nitrate hexahydrate (Ni(NO_3_)_2_·6H_2_O, 99%), zinc nitrate hexahydrate (Zn(NO_3_)_2_·6H_2_O, 99%), cupric nitrate trihydrate (Cu(NO_3_)_2_·3H_2_O, 99%), thiourea (CH_4_N_2_S, 99%), and citric acid monohydrate (C_6_H_8_O_7_·H_2_O, 99.8%) were purchased from Sinopharm Chemical Reagent Co., Ltd. and used directly without further purification.

### Preparation of Mo–MXene

Mo-based MXene (Mo–MXene) was synthesized using the previously reported etching method [33]. Briefly, 1 g of Mo_2_TiAlC_2_ was introduced into 30 mL of concentrated HF solution (40 wt%) in a Teflon container and stirred magnetically at 55 °C for 72 h. Subsequently, the solution underwent centrifugation and subjected to multiple washes with deionized water. Finally, it was vacuum-dried at a temperature of 40 °C for a duration of 24 h.

### Preparation of Mo–MXene/MoS_2_

To begin, 0.3322 g of (NH_4_)_6_Mo_7_O_24_·4H_2_O and 0.6518 g of CH_4_N_2_S were introduced into 20 mL of deionized water and stirred for 30 min. Afterwards, 75 mg of Mo_2_TiC_2_ and 41.3 mg of C_6_H_8_O_7_·H_2_O were added to the solution and stirred for 30 min. Finally, the mixed solution was transferred to a 50 mL Teflon vessel and subjected to hydrothermal reaction at 200 °C for 18 h. After cooling, it underwent multiple washes with deionized water and ethanol, and then it was dried in a vacuum drying oven at 40 °C for 24 h.

### Preparation of Mo–MXene/Mo–Sn Sulfide

Initially, 0.3322 g of (NH_4_)_6_Mo_7_O_24_·4H_2_O, 0.15 g of SnCl_4_·5H_2_O, and 0.6518 g of CH_4_N_2_S were added to 20 mL of deionized water and agitated for 30 min. Afterwards, 75 mg of Mo_2_TiC_2_ and 41.3 mg of C_6_H_8_O_7_·H_2_O were added to the solution and stirred for 30 min. Finally, the mixed solution was transferred to a 50 mL Teflon vessel and subjected to hydrothermal reaction at 200 °C for 18 h. After cooling, it underwent multiple washes with deionized water and ethanol, and then it was dried in a vacuum drying oven at 40 °C for 24 h.

### Preparation of Mo–MXene/Mo–Metal Sulfides, Mo–MXene/Metal Sulfides, and Metal Sulfides

The synthesis method of Mo–MXene/Mo–metal sulfides (metal = Fe, Mn, Co, Ni, Zn, and Cu) was similar to the preparation process of Mo–MXene/Mo–Sn sulfide, except that SnCl_4_·5H_2_O was replaced with other metal salts, such as FeCl_3_·6H_2_O, Mn(CH_3_COO)_2_·4H_2_O, Co(NO_3_)_2_·6H_2_O, Ni(NO_3_)_2_·6H_2_O, Zn(NO_3_)_2_·6H_2_O, Cu(NO_3_)_2_·3H_2_O. Similarly, the preparation process of Mo–MXene/metal sulfides (metal = Sn, Fe, Mn, Co, Ni, Zn, and Cu) only differ in that (NH_4_)_6_Mo_7_O_24_·4H_2_O was not added. For comparison, pure metal sulfide (metal = Sn, Fe, Mn, Co, Ni, Zn, and Cu) was synthesized from metal salts and thiourea under the same hydrothermal reaction conditions.

### Materials Characterization

The crystalline phases of the samples were analyzed using an X-ray diffraction spectrometer (XRD, D8-Advance, Bruker, Germany) utilizing a Cu–Ka radiation source (*λ* = 0.15406 nm, 40 kV, 30 mA). The microstructure of the samples was investigated by field emission scanning electron microscopy (FE-SEM, HITACHI SU8010, Japan) and transmission electron microscopy (TEM, JEM-2100F, Japan). Energy dispersive X-ray spectroscopy (EDS) was used for elemental mapping of the samples. X-ray photoelectron spectroscopy (XPS, Thermo escalade 250Xi) measurements were taken to record the chemical composition of the samples. Raman spectra were measured by LabRAM HR800 (HORIBA) with a 532 nm laser excitation wavelength. The electrochemical impedance spectroscopy (EIS) of the sample was tested using an electrochemical workstation (CHI 760E, Shanghai Huachen Instrument Co. Ltd., China).

### Electromagnetic Parameter Measurement

The electromagnetic properties of the EMW absorber in the frequency range of 2–18 GHz were measured using a vector network analyzer (Agilent E5071C) through the coaxial transmission method. EMW absorber rings were created by combining samples with paraffin wax, before being compacted into a ring with an inner diameter of 3.04 mm, an outer diameter of 7.00 mm, and a thickness of 2.00–3.00 mm. The mass ratio of the sample to paraffin wax was 7:3.

The EMW absorption characteristics of the product were determined by the reflection loss (*R*_L_) values that conform with transmission line assumptions. These values can be evaluated through the relationship between complex permittivity (*ɛ*_r_) and complex permeability (*µ*_r_).

1 $$R_{{\text{L}}} = {2}0{\text{log}}_{{{1}0}} \left| {\left( {Z_{{{\text{in}}}} - Z_{0} } \right)/\left( {Z_{{{\text{in}}}} + Z_{0} } \right)} \right|$$

2 $$Z_{{{\text{in}}}} = Z_{0} \left( {\mu_{{\text{r}}} /\varepsilon_{{\text{r}}} } \right)^{{{1}/{2}}} {\text{tanh}}[j({\text{2p}}fd/c)\left( {\mu_{{\text{r}}} \varepsilon_{{\text{r}}} } \right)^{{{1}/{2}}} ]$$

where *Z*_in_ and *Z*_0_ represent the input characteristic impedance and free space impedance, respectively. The velocity, thickness, and frequency are represented by *c*, *d*, and *f*, respectively.

### Radar Cross-Section and Density Functional Theory Simulations

To investigate the EMW absorption mechanism and practical application of the Mo–MXene/Mo–metal sulfides (metal = Sn, Fe, Mn, Co, Ni, Zn, and Cu) in depth, the radar cross-section (RCS) was simulated by a computer simulation technology (CST) program (see details in the Supporting Information).

To further reveal the charge density of the EMW absorbers, density functional theory (DFT) calculations were conducted (see details in the Supporting Information).

## Results and Discussion

### Composition and Microstructure

**Scheme 1:**
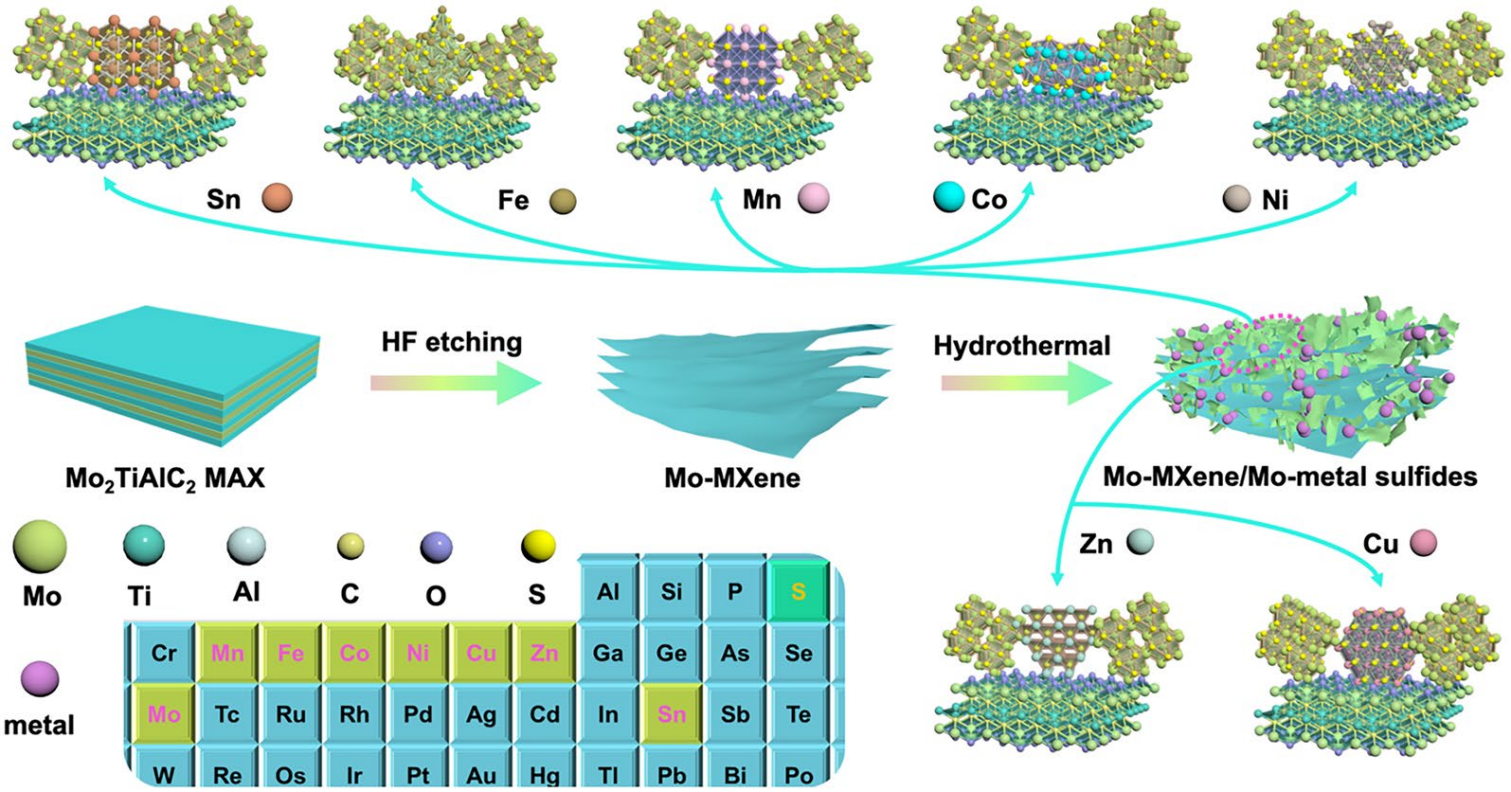
Illustration of the synthesis process for Mo–MXene/Mo–metal sulfides

The synthetic pathway for producing the Mo–MXene/Mo–metal sulfides (semiconductor–semiconductor–metal mixed-phase) heterostructures is depicted in Scheme 1. Initially, the Al layer of Mo_2_TiAlC_2_ MAX was corroded using HF solution to create a multilayer Mo-based MXene. Subsequently, MoS_2_ was grown on the surface and interlayer of the layered Mo–MXene through a straightforward hydrothermal synthesis process. In this procedure, MoS_2_ acted as a *n*-type semiconductor, forming a semiconductor–metal heterojunction with the metallic Mo–MXene phase. However, the successful formation of a semiconductor–metal heterojunction led to issues with poor impedance matching due to the high conductivity of Mo–MXene/MoS_2_. To amplify the EMW absorption capabilities of MXene-based materials, we introduced seven additional metal ions (Sn^4+^, Fe^3+^, Mn^2+^, Co^2+^, Ni^2+^, Zn^2+^, and Cu^2+^) as supplementary semiconductors during the hydrothermal process. This incorporation aimed to enhance the EMW absorption performance. The "one-pot" hydrothermal synthesis process facilitated the creation of both MoS_2_ semiconductor phases and metal sulfides (metal = Sn, Fe, Mn, Co, Ni, Zn, and Cu) semiconductor phases. The combination of these two semiconductor phases with the Mo–MXene metallic phase resulted in the formation of two semiconductor–metal heterojunctions and semiconductor–semiconductor heterojunctions. These heterojunctions synergistically contributed to promoting the EMW absorption properties.

**Fig. 1:**
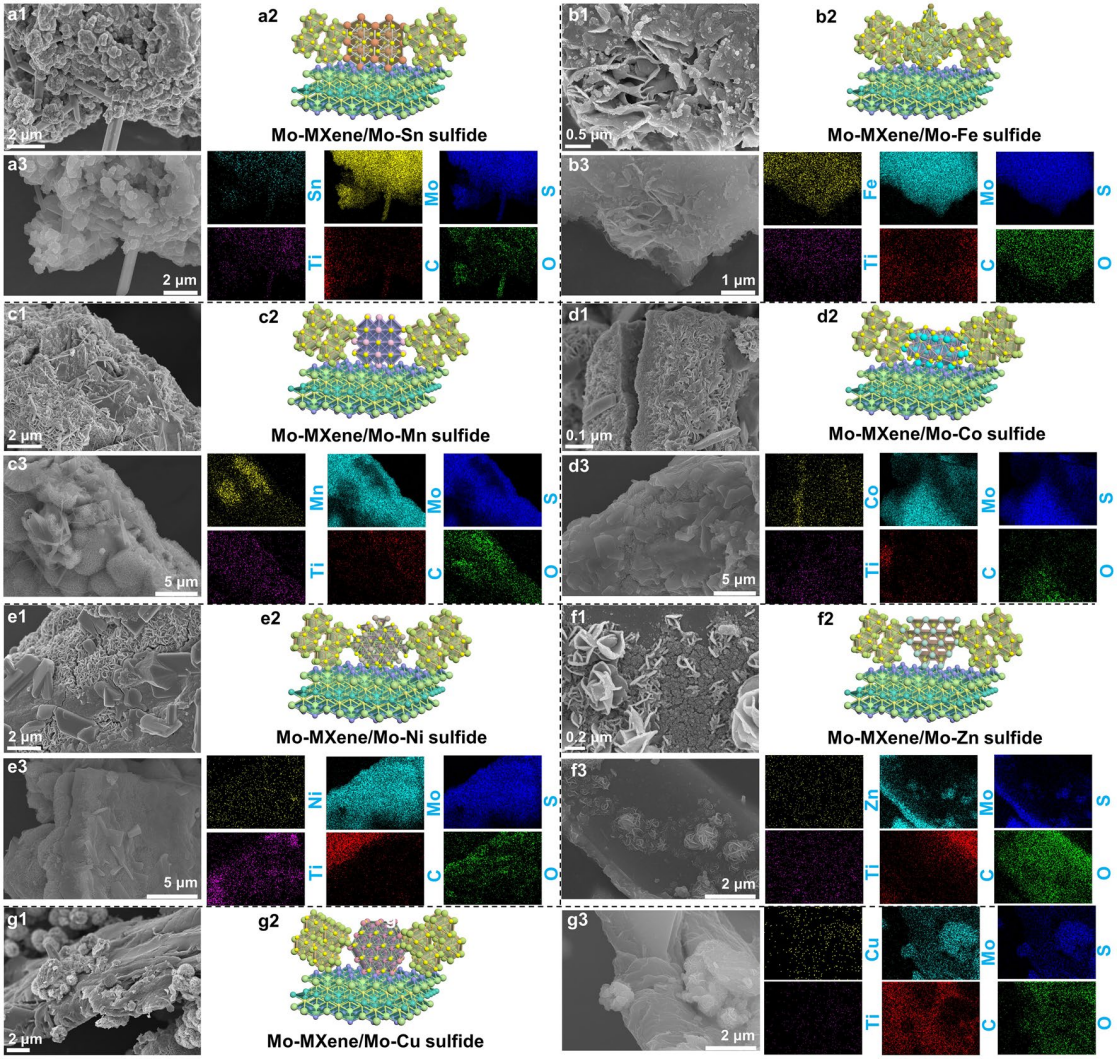
SEM images, atomic structure, and EDS mapping images of **a** Mo–MXene/Mo–Sn sulfide, **b** Mo–MXene/Mo–Fe sulfide, **c** Mo–MXene/Mo–Mn sulfide, **d** Mo–MXene/Mo–Co sulfide, **e** Mo–MXene/Mo–Ni sulfide, **f** Mo–MXene/Mo–Zn sulfide, and **g** Mo–MXene/Mo–Cu sulfide

Figure S1 shows the scanning electron microscopy (SEM) image of Mo–MXene/MoS_2_. Clearly, the ultrathin MoS_2_ nanosheets with a thickness of ~ 8 nm are uniformly grown on the surface of Mo–MXene. After adding the second metal element, the formed Mo–MXene/Mo–metal sulfide (metal = Sn, Fe, Mn, Co, Ni, Zn, and Cu) heterostructure exhibits a slightly different structure. Specifically, small flower-like structures are observed in Sn, Zn, and Cu systems (Figs. 1a1, f1, g1 and S2a, f, g). Furthermore, nanoparticles, nanoplates, and nanoplates appear in the Mo–MXene/Mo-Fe sulfide, Mo–MXene/Mo–Co sulfide, and Mo–MXene/Mo–Ni sulfide systems (Figs. 1b1, d1, e1 and S2b, d, e). In addition, nanofibers exist in Mo–MXene/Mo–Mn sulfide (Fig. 1c1 and S2c). In conclusion, compared to Mo–MXene/MoS_2_, the appearance of different morphologies is beneficial for constructing semiconductor–semiconductor–metal heterostructures. This tailored design strategy aims to establish rich heterogeneous interfaces to enhance interfacial polarization.

As illustrated in Fig. S3, in addition to the morphological changes, there are also significant differences in the compositions of Mo–MXene/Mo–metal sulfides in the X-ray diffraction (XRD) pattern. In all samples, discernible diffraction peaks corresponding to 2H–MoS_2_ (PDF#37–1492), 1T–MoS_2_ [15], and Mo_2_TiC_2_ MXene are detected [34]. The investigation of Mo–MXene/Mo–Sn sulfide shows the emergence of Sn_2_S_3_ phase (PDF#14-0619) (Figs. 1a2 and S3a), indicating that indicating that some S^2−^ ions bind to Sn^4+^ except for Mo^4+^. Similarly, the substitution of Sn^4+^ with Fe^3+^ yields corresponding sulfide phases, namely FeS_2_ (PDF#74–1051) and Fe_7_S_8_ (PDF#76-2308), alongside other existing phases in the heterostructure (Figs. 1b2 and S3b). Upon the introduction of various other metal ions (Mn^2+^, Co^2+^, Ni^2+^, Zn^2+^, and Cu^2+^) to the reaction system, some sulfur ions react with the respective metal ions to form metal sulfides. Specifically, these sulfides include MnS (PDF#88-2223), CoS (PDF#75–0605), Ni_3_S_4_ (PDF#76-1813), ZnS (PDF#77-2100), and CuS (PDF#76-1725) (Figs. 1c2–g2 and S3c–g). Based on these findings, we infer that the coexistence of MoS_2_, metal sulfides (metal = Sn, Fe, Mn, Co, Ni, Zn, and Cu), and Mo–MXene substantiates, confirming the formation of semiconductor–semiconductor–metal heterostructures. Energy-dispersive X-ray spectroscopy (EDS) confirms the presence of Mo, Ti, C, O, S, and corresponding metal elements, as well as the uniform dispersion in the heterostructure (Fig. 1a3–g3).

To gain a comprehensive understanding of the influence of the metal ions, we further added other metal ions (Sn^4+^, Fe^3+^, Mn^2+^, Co^2+^, Ni^2+^, Zn^2+^, and Cu^2+^) in the absence of Mo^4+^. Apparently, we observed that the presence of MoS_2_ plays a pivotal role in forming the morphology of the resultant samples. Specifically, hexagonal nanosheets are found in Mo–MXene/Sn sulfide heterostructures (Fig. S4a). This may be attributed to the competition between Mo^4+^ and Sn^4+^ for S^2−^, ultimately hindering the formation of hexagonal nanosheets in Sn sulfide. Moreover, small nanocubes, irregular polyhedra, dodecahedra, large cubes, small nanoparticles, and nanosheets are found in Mo–MXene/Fe sulfide (Fig. S5a), Mo–MXene/Mn sulfide (Fig. S6a), Mo–MXene/Co sulfide (Fig. S7a), Mo–MXene/Ni sulfide (Fig. S8a), Mo–MXene/Zn sulfide (Fig. S9a), and Mo–MXene/Cu sulfide (Fig. S10a), respectively. Similarly, it may also be the result of competition between metal ions (Fe^2+^, Mn^2+^, Co^2+^, Ni^2+^, Zn^2+^, Cu^2+^) and Mo^4+^ for S^2−^. On the other hand, it can be noticed from Fig. S11 that compared to the samples with the addition of Mo–MXene, the metal sulfides synthesized separately undergo self-assembly process. For example, Fe sulfide and Zn sulfide will self-assemble into spherical structures (Fig. S11b, f), Cu sulfide will form a flower-like structure (Fig. S11g), and other metal sulfide (metal = Sn, Mn, Co, and Ni) will exhibit certain agglomeration phenomena (Fig. S11a, c–e). However, when Mo–MXene is present, the synthesized metal sulfides do not self-assemble, but instead grow or attach to the Mo–MXene substrate to form Schottky junctions (semiconductor–metal heterojunction).

Further analysis by XRD corroborated the presence of corresponding metal sulfide phases in the sample (Figs. S3 and S4b–S10b). Notably, in the systems of Sn, Fe, or Co sulfides, the introduction of Mo^4+^ leads to changes in the crystalline phase (Fig. S3(a, b, d), S4b, S5b, and S7b). In other systems, the crystalline phase remains unchanged (Figs. S3(c, e–g), S6b, S8b–S10b). The XRD pattern of pure metal sulfides is shown in Fig. S12. Obviously, except for Mo–MXene, the phase of pure metal sulfides is consistent with that of Mo–MXene/metal sulfide (metal = Sn, Fe, Mn, Co, Ni, Zn, and Cu). These results indicate that the addition of MoS_2_ not only affects the morphology, but also to some extent affects the composition. This may be the result of the competition between Mo^4+^ and other metal ions for S^2−^. Based on XRD and SEM results, it can be demonstrated that the introduction of Mo–MXene does not change the crystalline phase of the sample, but mainly serves as a carrier for metal sulfides. EDS elemental mapping images further indicates that metal sulfides are mainly distributed on Mo–MXene (Figs. S4c–S10c), which is consistent with the SEM results.

**Fig. 2:**
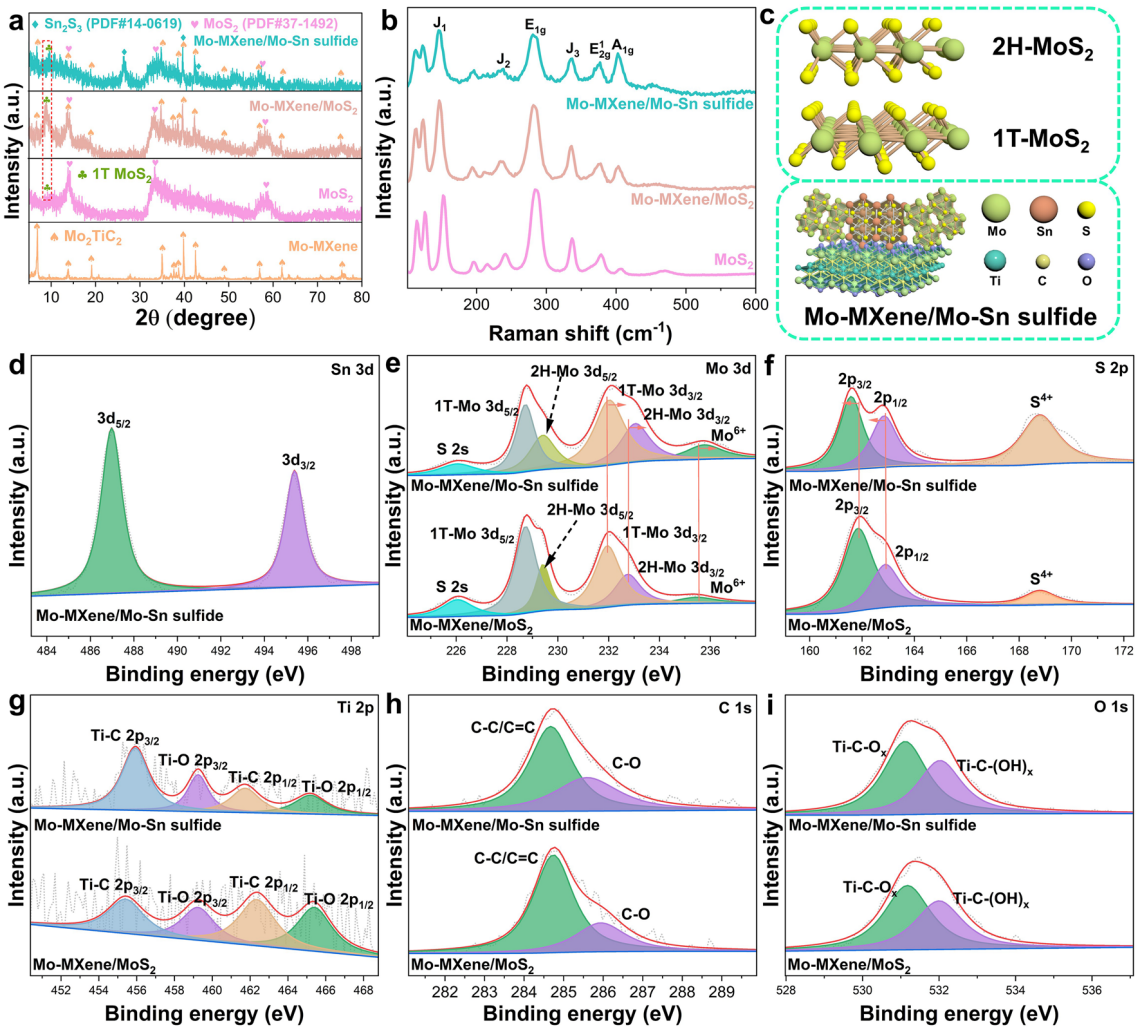
**a** XRD patterns and **b** Raman spectra of the samples. **c** Schematic of atomic structure for 2H–MoS_2_, 1T–MoS_2_, and Mo–MXene/Mo–Sn sulfide. High-resolution XPS spectra of **d** Sn 3*d*, **e** Mo 3*d*, **f** S 2*p*, **g** Ti 2*p*, **h** C 1*s*, and **i** O 1*s* for Mo–MXene/Mo–Sn sulfide and Mo–MXene/MoS_2_

For Mo–MXene/Mo–metal sulfides system, Mo–MXene/Mo–Sn sulfide is chosen as the representative material to investigate the effect of added metal ions on its composition, morphology, and electromagnetic properties. As illustrated in XRD patterns, the diffraction peaks of layered Mo–MXene align with prior findings (Fig. 2a) [35], affirming the successful synthesis of MXene. The XRD analysis of the MoS_2_ reveal the diffraction peaks at 14.38°, 33.4°, and 58.3°, corresponding to the (002), (101), and (110) crystal planes of 2H–MoS_2_ (PDF#37-1492) phase, respectively. Intriguingly, a newly observed diffraction peak at 9.22° can be attributed to the (002) crystal plane of 1T–MoS_2_ [15]. The XRD patterns of Mo–MXene/Mo–Sn sulfide and Mo–MXene/MoS_2_ are scrutinized, revealing that the physical phases of Mo–MXene remains unaffected in both cases. This finding underscores the preservation of the layered structure of Mo–MXene during the synthesis process. Further XRD analysis unveils the presence of both 2H–MoS_2_ and 1T–MoS_2_ phases. Notably, the slight low-angle shift of the (110) crystal plane at 58.3° indicates an enlargement of the interlayer spacing in the prepared samples. Furthermore, Mo–MXene/Mo–Sn sulfide also exhibits diffraction peaks at 26.59°, 39.86°, and 42.8°, corresponding to the (111), (160), and (420) crystal planes of the Sn_2_S_3_ (PDF#14-0619) phase. To further substantiate the existence of 1T and 2H phases of MoS_2_ in the composites, Raman spectroscopic analysis of Mo–MXene/Mo–Sn sulfide, Mo–MXene/MoS_2_, and MoS_2_ was performed (Fig. 2b). The results exhibit three distinct peaks at 282.2, 376.8, and 403.9 cm^−1^, corresponding to the Raman vibrational modes of E_1g_, E_2g_^1^, and A_1g_ of 2H–MoS_2_, respectively [36]. Additionally, distinctive peaks at 145.6, 236.9, and 335.3 cm^−1^ are evident, corresponding to the J_1_, J_2_, and J_3_ vibrational modes of 1T–MoS_2_, respectively [37]. Compared to MoS_2_, the peak intensity of Raman vibrational modes in Mo–MXene/MoS_2_ and Mo–MXene/Mo–Sn sulfide decreases, indicating that some of the 2H–phase MoS_2_ transforms into the 1T-phase MoS_2_. These XRD and Raman results collectively support the presence of semiconductor–metal heterojunctions within the MoS_2_ structure. This phenomenon is attributed to the semiconductor nature of the 2H phase and the metallic characteristics of the 1T phase. The presence of the 1T–2H phase in MoS_2_ may enhance the BIEF constructed by the Schottky junction. Moreover, the presence of Mo–MXene metallic phase and Sn_2_S_3_ semiconductor phase further supports the idea of forming a semiconductor–semiconductor–metal heterojunction during the synthesis process (Fig. 2c).

X-ray photoelectron spectroscopy (XPS) investigation of Mo–MXene/Mo–Sn sulfide and Mo–MXene/MoS_2_ reveal the presence of Sn, Mo, S, Ti, C, and O elements (Fig. S13). The molar ratio of Mo–Sn is 5:1, indicating a small amount of Sn doping. In the high-resolution XPS spectrum of Sn 3*d*, the peaks at binding energies of 486.98 and 495.39 eV correspond to Sn 3*d*_5/2_ and 3*d*_3/2_, respectively (Fig. 2d) [19]. The high-resolution XPS spectrum of Mo 3*d* reveals that the peaks at 233.06 and 229.42 eV are associated with Mo 3*d*_3/2_ and Mo 3*d*_5/2_ of Mo^4+^ in the semiconductor 2H–MoS_2_ phase (Fig. 2e). Additionally, peaks located at 232.03 and 228.73 eV correspond to Mo 3*d*_3/2_ and Mo 3*d*_5/2_ of the metal 1T–MoS_2_ phase. The presence of both 2H and 1T phases in the Mo elements further confirms the formation of semiconductor–metal heterostructures in the MoS_2_ species. The two weaker peaks at 226.05 and 235.77 eV correspond to S 2*s* and Mo^6+^, respectively, indicating that S ions are bonded to Mo ions and that oxidation states of Mo elements are present [37]. Raman spectra show that the peaks at 662, 818, and 992 cm^−1^ correspond to the stretching modes of three-coordinated oxygen (3Mo–O), double-coordinated oxygen (2Mo–O), and terminal oxygen (Mo^6+^-O) (Fig. S14), respectively. This further explains the oxidation of elemental Mo. The high-resolution XPS spectrum of S 2*p* (Fig. 2f) display peaks at 168.8 eV, 161.59 eV (S 2*p*_3/2_), and 162.85 eV (S 2*p*_1/2_), attributed to the binding energies of the S^4+^ species of MoS_2_, bridging S_2_^2−^, and apical S^2−^ sites, respectively. This indicates the presence of S-edge sites in Mo–MXene/Mo–Sn sulfide [38]. A subtle binding energy shift in S 2*p* and Mo 3*d* can be observed in Mo–MXene/Mo–Sn sulfide compared to Mo–MXene/MoS_2_, as evident from the XPS spectra. This result reveals a strong electronic interaction between S and Mo elements upon introducing Sn elements into Mo–MXene/MoS_2_ [39]. This interaction is known to create defects and promote efficient electron transfer at semiconductor–semiconductor–metal heterointerfaces [35, 40]. Regarding the high-resolution XPS spectrum of Ti 2*p* for Mo–MXene/Mo–Sn sulfide and Mo–MXene/MoS_2_ (Fig. 2g), the binding energies of Ti–O 2*p*_1/2_ and 2*p*_3/2_ are located at 465.2 and 459.26 eV, respectively, while the binding energies of Ti–C 2*p*_1/2_ and 2*p*_3/2_ are centered at 461.75 and 455.94 eV, respectively. The presence of Ti–O bonds is attributed to weak oxidation of the MXene surface [41]. The absence of Ti–Al bonds in the Ti 2*p* XPS spectrum indicates that HF etching removes the Al layer. In the high-resolution XPS spectrum of C 1*s* (Fig. 2h), the peaks at 284.68 and 285.6 eV correspond to C–C/C = C and C–O bonds. The high-resolution XPS spectrum of O 1*s* show the Ti–C–O_x_ bond at 530.9 eV and the Ti–C–(OH)_x_ bond at 531.8 eV (Fig. 2i) [42]. The O 1*s* spectrum further confirms the weak oxidation of the MXene surface.

**Fig. 3:**
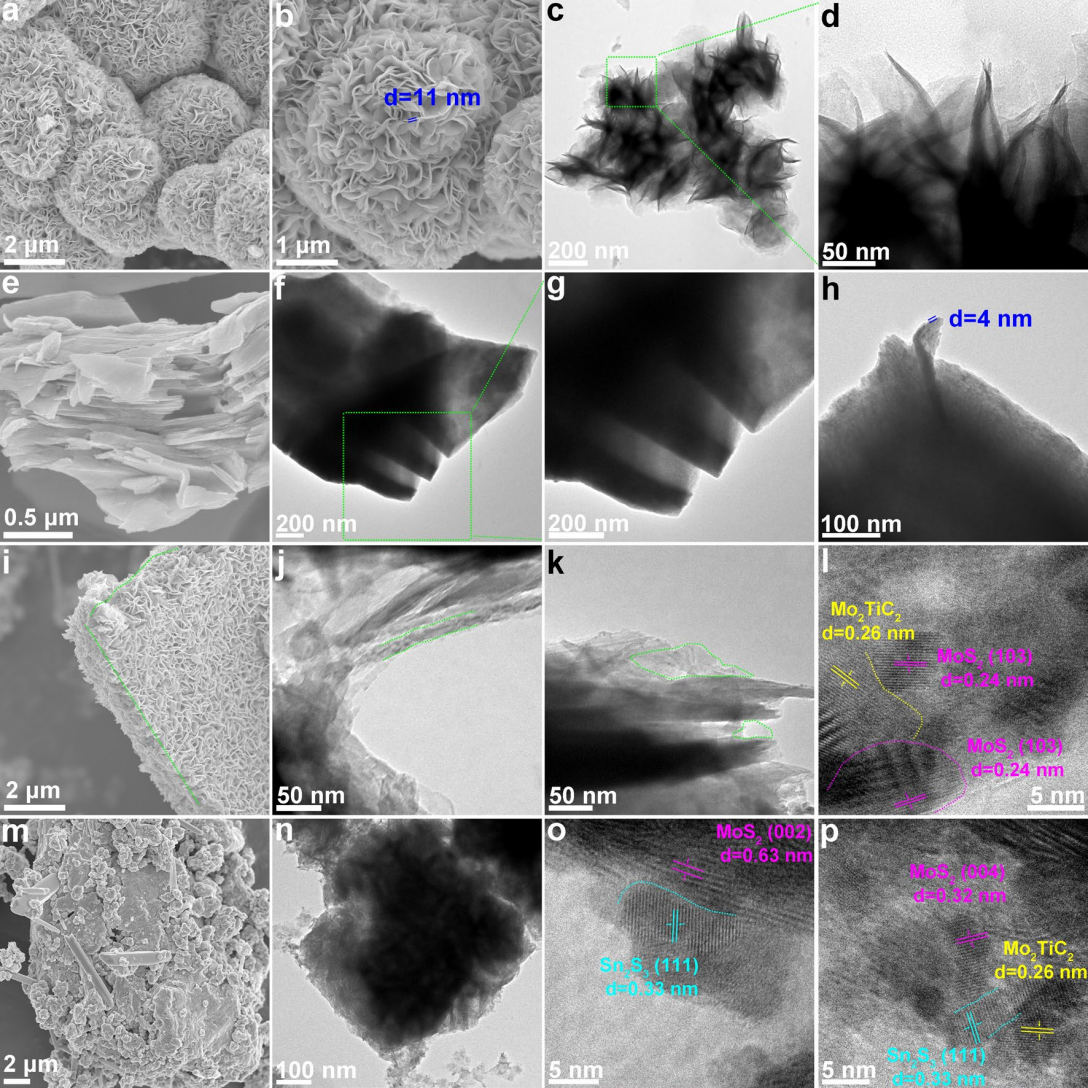
SEM, TEM, and HRTEM images of **a–d** MoS_2_, **e–h** Mo–MXene, **i–l** Mo–MXene/MoS_2_, and **m–p** Mo–MXene/Mo–Sn sulfide

For a deeper understanding, the morphological and structural characteristics of MoS_2_, Mo–MXene, Mo–MXene/MoS_2_, and Mo–MXene/Mo–Sn sulfide were examined using SEM and TEM. Figure 3a–d displays the successful synthesis of MoS_2_ samples characterized by nanoflowers. MoS_2_ nanoflowers are assembled by numerous nanosheets with a thickness of ~ 11 nm. Figure 3e–h reveals the accordion-like structure of Mo–MXene with distinct lamellae (thickness ≈ 4 nm) and smooth surfaces, confirming the successful removal of Al layer. Additionally, Fig. 3i presents the SEM images of Mo–MXene/MoS_2_, retaining the layered structure of Mo–MXene while incorporating MoS_2_ nanosheets on the surface and interlayer of Mo–MXene. The EDS elemental mapping images further demonstrate the combination of MoS_2_ and Mo–MXene (Fig. S15). In Fig. 3j, k, uniform growth of MoS_2_ nanosheets on Mo–MXene can be observed. The MoS_2_ nanosheets that tend to grow vertically contribute to increasing surface area and improving electromagnetic wave dissipation pathways. Notably, lattice spacings of 0.24 and 0.26 nm in high-resolution TEM (HRTEM) correspond to MoS_2_ (103) and Mo_2_TiC_2_ crystal faces, respectively (Fig. 3l) [43, 44], implying the coexistence of MoS_2_ and Mo_2_TiC_2_ and suggesting the formation of semiconductor–metal heterointerfaces. Meanwhile, the (006) crystal face of Mo_2_TiC_2_ MXene is further depicted in Fig. S16, corresponding to a crystal face spacing of 0.45 nm [45]. The microstructure of Mo–MXene/Mo–Sn sulfide shows that the addition of Sn will compete with Mo^4+^ for S^2−^ to a certain extent, resulting in changes in the morphology of Mo–MXene/MoS_2_, but the growth of MoS_2_ nanosheets is still preserved (Fig. 1a1). Moreover, due to the formation of Sn_2_S_3_, a unique structure emerges in Mo–MXene/Mo–Sn sulfide, with fine particles attaching to the nanosheets (Figs. 3m and S18a). This distinct structure provides numerous heterogeneous interfaces, facilitating the formation of semiconductor–semiconductor–metal heterointerfaces and enhancing electron transfer capability and interfacial polarization [46]. Figures 3n and S17 further confirm the growth of MoS_2_ nanosheets on Mo–MXene. Figures 3o, p and S18b, c demonstrate lattice spacings of 0.63 and 0.32 nm, which can be related to the (002) and (004) crystal facets of MoS_2_, respectively [47, 48]. Additionally, an observed lattice spacing of 0.33 nm in HRTEM is attributed to the (111) crystal face of Sn_2_S_3_ [49]. The lattice spacing of 0.24 nm in HRTEM further confirms the presence of MoS_2_ (103) crystal faces (Fig. S18d). These results further indicate the presence of a semiconductor–semiconductor–metal hetero-interface, corroborating the successful formation of the Mo–MXene/Mo–Sn sulfide heterostructure.

### EMW Absorption Performance

**Fig. 4:**
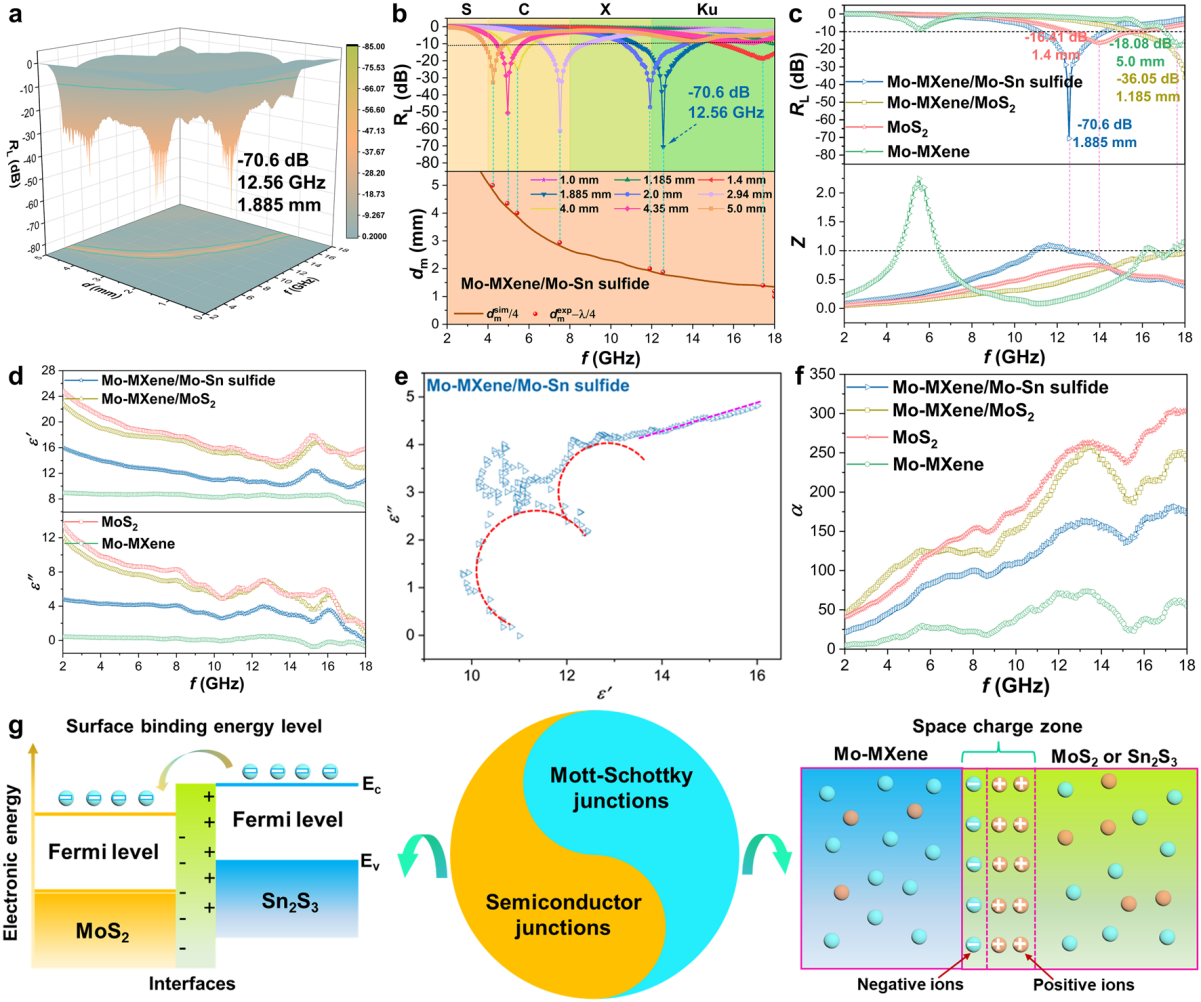
**a** 3D *R*_L_ values of Mo–MXene/Mo–Sn sulfide. **b** 2D *R*_L_ values and simulation of *d*_m_ (*d*_m_^sim^) vs. *f*_m_ curves for Mo–MXene/Mo–Sn sulfide. **c** Optimized 2D *R*_L_ and *Z* values of the samples. **d** Permittivity of the samples. **e** *ɛ*′-*ɛ*′′ curves of Mo–MXene/Mo–Sn sulfide. **f** *α* values of the samples. **g** Schematic illustration of BIEF formation between semiconductor and metallic phases

Comparative analysis of the electromagnetic wave (EMW) absorption performance of MoS_2_, Mo–MXene, Mo–MXene/MoS_2_, and Mo–MXene/Mo–Sn sulfide is presented, with detailed data depicted in Figs. 4a and S19. Remarkably, Mo–MXene/Mo–Sn sulfide demonstrates exceptional EMW absorption performance, exhibiting a reflection loss (*R*_L_) value of − 70.6 dB at a matching thickness of only 1.885 mm (Fig. 4a). This result can be predominantly attributed to the formation of a semiconductor–semiconductor–metal mixed phase in the sample. Interestingly, Mo–MXene/Mo–Sn sulfide also displays low *R*_L_ values (< − 45.0 dB) in the C-band (4–8 GHz) and X-band (8–12 GHz), accompanied by matching thicknesses below 5 mm (Fig. 4b). Adhering to the quarter-wavelength theory (*d*_*m*_ = *nc*/(4* fm*(|*μ*_*r*_||*ε*_*r*_|)^1/2^), *n* = 1, 3, 5, …), the findings demonstrate that the values of *d*_m_ and *f*_m_ for Mo–MXene/Mo–Sn sulfide adhere to the expected pattern. Consequently, Mo–MXene/Mo–Sn sulfide exhibits optimal EMW absorption performance and impeccable impedance matching compared to the other three samples (Fig. 4c). An in-depth analysis of the Sn system indicates that the absence of MoS_2_ significantly impairs the EMW absorption capacity (Fig. S20). The poor EMW absorption performance of MoS_2_, Mo–MXene, Mo–MXene/MoS_2_, Mo–MXene/MoS_2_, Mo–MXene/Sn sulfide, and Sn sulfide fully identifies the synergistic effect of MoS_2_, Mo–MXene, and Mo–Sn sulfide. This performance enhancement should be attributed to the special electric field established between the semiconductor junction and Schottky junction, which increases the electron transport capacity [15]. It is also proved that the superiority of the constructed semiconduction–semiconduction–metal heterostructure and the presence of 1T–2H phase enhance the lifting effect of the semiconduction–metal junction.

To elucidate the EMW absorption characteristics of the samples, an exploration of its complex permittivity (*ɛ*_r_ = *ɛ′*—j*ɛ*ʺ) and complex permeability (*μ*_r_ = *μ′*—j*μ*ʺ) was conducted. Here, *ɛ′* and *μ′* represent the storage capabilities of EM waves, while *ɛ*ʺ and *μ*ʺ reflect the dissipation capacities of EM waves. Generally, excessively high or low values of the complex permittivity are undesirable for optimal impedance matching [50]. As depicted in Fig. 4d, the value of the complex permittivity exhibits a declining trend with increasing frequency, attributed to the polarization hysteresis phenomena observed at higher frequencies. A conspicuous polarization peak is discernible within the Ku band, signifying the presence of polarization loss. In the four samples, the order of the complex permittivity from high to low is MoS_2_, Mo–MXene/MoS_2_, Mo–MXene/Mo–Sn sulfide, and Mo–MXene. Notably, MoS_2_ yields the highest permittivity, indicative of its enhanced conductivity. Following the electron mobility theory (*ɛ*ʺ = *σ*/(2*πɛ*_0_*f*)), heightened conductivity leads to robust EMW reflection, which is detrimental to impedance matching (Fig. 4c) [51]. Mo–MXene has the lowest permittivity, possibly due to the introduction of numerous F^−^ surface groups during the HF etching process [52]. With the incorporation of Sn element, the excessive conductivity in the material is improved. This signifies that Mo–MXene/Mo–Sn sulfide attains superior performance and impeccable impedance matching. As demonstrated in Fig. S21, it becomes evident that the permittivity values of Mo–MXene/Sn sulfide and Sn sulfide are excessively low, contributing to the subpar EMW absorption performance. Therefore, the coexistence of Mo–MXene, Sn_2_S_3_, and MoS_2_ is instrumental in regulating the permittivity to an optimal level, thus enhancing absorption performance. Additionally, the electrical conductivity of Mo–MXene/Mo–Sn sulfide is verified to be different from that of Mo–MXene/MoS_2_ by EIS (Fig. S22). This result indicates that heterostructures composed of semiconductor junctions and Mott–Schottky junctions have a modulation effect on the electron transfer of the material [53–55]. In particular, the formation of semiconductor–semiconductor and metal–semiconductor heterogeneous interfaces in Mo–MXene/Mo–Sn sulfide engenders strong interfacial polarization. As evidenced in Figs. S23 and S24, the permeability curve exhibits strong resonance peaks in the high-frequency region, indicating the presence of exchange resonance. *µ′* remains close to 1, *µ*ʺ is nearly 0, and tan *δ*_µ_ fluctuates around 0. This indicates that dielectric loss dominates the EMW absorption capacities of the samples. On the other hand, the fluctuation of the curve within the testing frequency range indicates the presence of natural and exchange resonances, with dielectric loss dominates. Furthermore, *µ*ʺ assumes a negative value at low frequencies, primarily due to the generation of a magnetic field opposing the incident EM wave induced by the BIEF. According to Maxwell’s equations, when the charges in the BIEF initiate movement, a counteractive magnetic field is generated. In materials with high electrical conductivity, a magnetic field arises to counteract the external magnetic field, leading to the suppression of magnetic losses and the emergence of negative *μ*ʺ values [56].

According to the Debye theory ((*ɛ′*–*ɛ*_∞_)^2^ + (*ɛ*ʺ)^2^ = (*ɛ*_s_–*ɛ*_∞_)^2^), the relationship between *ɛ′* and *ɛ*ʺ is linked to Debye dielectric relaxation [57]. From Figs. 4e and S25, it can be found that Mo–MXene/Mo–Sn sulfides exhibit excellent polarization loss [58, 59]. Moreover, the presence of MoS_2_ can provide additional conduction loss for the Mo–MXene/Mo–Sn sulfide heterostructures and enhance the attenuation ability [60, 61]. In addition, both Mo–MXene/Sn sulfide and Sn sulfide exhibit Cole–Cole curves similar to Mo–MXene (Fig. S26). This indicates that the introduction of Sn elements has a limited effect on their polarization relaxation. The total *ε*″ is composed of the contributions from conduction loss (*ε*″_*σ*_) and polarization loss (*ε*″_p_), and can be expressed as *ε*″ = *ε*″_*σ*_ + *ε*″_p_ = *σ*/(2π*fε*_0_) + 2π*fτ*(*ε*_s_–*ε*_∞_)/(1 + (2π*f*)^2^*τ*^2^) [57, 60]. Therefore, the polarization loss in Mo–MXene/Mo–Sn sulfides plays a major role in the dielectric loss. Polarization loss can be divided into interfacial polarization, dipole polarization, ionic polarization, and electronic polarization. In general, ionic polarization and electronic polarization occur at 10^3^–10^6^ GHz [35]. Therefore, interfacial polarization and dipole polarization are part of dielectric loss mechanism for the Mo–MXene/Mo–Sn sulfide.

In the context of EMW absorption, the parameters of eddy current loss (*C*_0_ = *μ"*(*μ′*)^−2^*f*^−1^ = 2π*μ*_0_*d*^2^*ϭ*) and attenuation constant (*α* = [((2)^1/2^π*f*)/*c*] × {(*μ"ε"*—*μ′ε′*) + [(*μ"ε"*—*μ′ε′*)^2^ + (*ε′μ"* + *ε"μ′*)^2^]^1/2^}^1/2^) play a crucial role in assessing EMW absorption capabilities [62]. As indicated by the observations in Figs. S27 and S28, the curve within the range of 6–18 GHz exhibits nearly linear behavior, confirming the presence of eddy current losses. Furthermore, the *C*_0_ curve of the material exhibits nonlinear behavior in the low frequency region (2–6 GHz), which is mainly attributed to the presence of natural resonance [51]. In terms of *α* values (Fig. 4f), the order is MoS_2_, Mo–MXene/MoS_2_, Mo–MXene/Mo–Sn sulfide, and Mo–MXene, in descending order. MoS_2_ and Mo–MXene/MoS_2_ exhibit similar *α* values, with MoS_2_ having the larger value. Excessively high *α* values can result in significant EM waves reflection [51]. In addition, the *α* value of Mo–MXene/Mo–Sn sulfide is much larger than that of Mo–MXene/Sn sulfide and Sn sulfide (Fig. S29), confirming that a low *α* value is detrimental to improving performance. In Fig. 4g, the deviation in Fermi energy levels between the semiconductor MoS_2_ and semiconductor Sn_2_S_3_ gives rise to bending of semiconductor energy bands at the interface. This effect fosters the creation of a BIEF as electrons flow from Sn_2_S_3_ to MoS_2_, augmenting dielectric loss improvement. Around heterogeneous interfaces, the distribution of positive and negative charges induces interfacial polarization, enhancing dissipation capabilities. Furthermore, the formation of a Schottky barrier between the metal-phase Mo–MXene and semiconductor metal sulfide (MoS_2_ and Sn_2_S_3_) contributes to the increased interfacial resistance. This phenomenon facilitates charge accumulation and the generation of interfacial polarization.

**Fig. 5:**
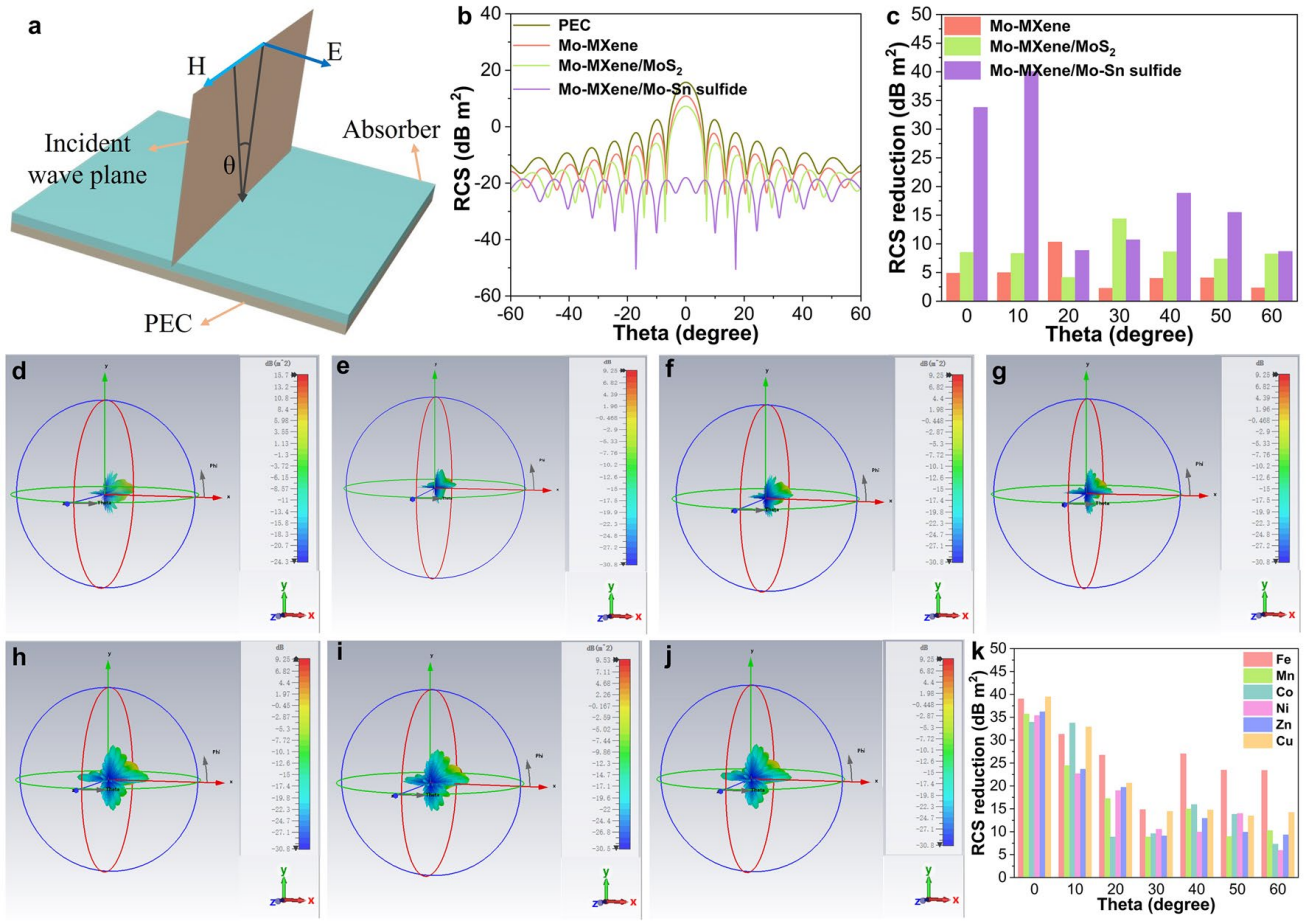
**a** RCS model. **b** 2D RCS values of PEC, Mo–MXene, Mo–MXene/MoS_2_, and Mo–MXene/Mo–Sn sulfide. **c** RCS reduction values of the samples. CST simulation results of PEC covered with Mo–MXene/Mo–metal sulfide, where metal is represented by **d** Sn, **e** Fe, **f** Mn, **g** Co, **h** Ni, **i** Zn, and **j** Cu. **k** RCS reduction values of Mo–MXene/Mo–metal sulfide (metal = Fe, Mn, Co, Ni, Zn, and Cu)

To investigate the potential of Mo–MXene/Mo–Sn sulfide in practical applications, the radar cross-section (RCS) was simulated by a computer simulation technology (CST) software [63]. The smaller the RCS value, the better the EMW absorption performance of the material [64]. Initially, the PEC layer was set as a rectangular ultrathin plane of 200 × 200 mm^2^, and the coating was also 200 × 200 mm^2^. The model used in the RCS simulation is depicted in Fig. 5a. Figure 5b shows the RCS values for PEC and the three coatings at different angles of incidence (− 60° to 60°). Compared with PEC, the RCS values of each model change gradually with increasing angle, and the Mo–MXene/Mo–Sn sulfide coating has the smallest RCS signal, especially at 0° detection angle. To evaluate the intrinsic EMW absorption capacity of different absorbers more comprehensively, the results for seven specific detection angles (0°, 10°, 20°, 30°, 40°, 50°, and 60°) were obtained by subtracting the RCS values of the PEC layer (Fig. 5c). All Mo–MXene -based materials have the ability to reduce RCS. Compared to Mo–MXene/MoS_2_ (4.13–14.4) and Mo–MXene (2.24–10.3), Mo–MXene/Mo–Sn sulfide showed a stronger RCS reduction capability at a range of 8.66–40 dB m^2^, which can also be demonstrated through Fig. S30a. These results are consistent with the *R*_L_ value, further confirming that Mo–MXene/Mo–Sn sulfide has a strong absorption capability. From Figs. 5d and S30b–d, it can be observed that the reflection signal changes correspondingly with the variation of the coating. Compared to the PEC model, the model covered with Mo–MXene/Mo–Sn sulfide exhibits a smaller reflection signal.

To verify the effectiveness of constructing BIEF between semiconductor and metallic phases, we also designed other Mo–MXene/Mo–metal sulfides (metal = Fe, Mn, Co, Ni, Zn, and Cu). The remaining six coatings (Fe, Mn, Co, Ni, Zn, and Cu) were simulated with sizes of 200 × 200 mm^2^. Figure 5e–j demonstrate that the reflected signals correspondingly change with variations in the coating. In comparison to the PEC model, the model covered with Mo–MXene/Mo–metal sulfide (metal = Fe, Mn, Co, Ni, Zn, and Cu) exhibits smaller reflected signals. All materials based on Mo–MXene possess the ability to reduce RCS. The Fe, Mn, Co, Ni, Zn, and Cu systems exhibit strong RCS reduction capabilities within the ranges of 14.8–39, 8.84–35.7, 7.27–33.92, 5.89–35.36, 9.07–36.17, and 13.45–39.42, as shown in Fig. 5k. Based on RCS simulations, it can be inferred that Mo–MXene/Mo–metal sulfide (metal = Fe, Mn, Co, Ni, Zn, and Cu) can also exhibit excellent EMW absorption capabilities in theory. The RCS simulation results further indicate that the constructed semiconductor–semiconductor–metal heterojunction has the potential to be utilized in different materials, demonstrating a degree of universality.

**Fig. 6:**
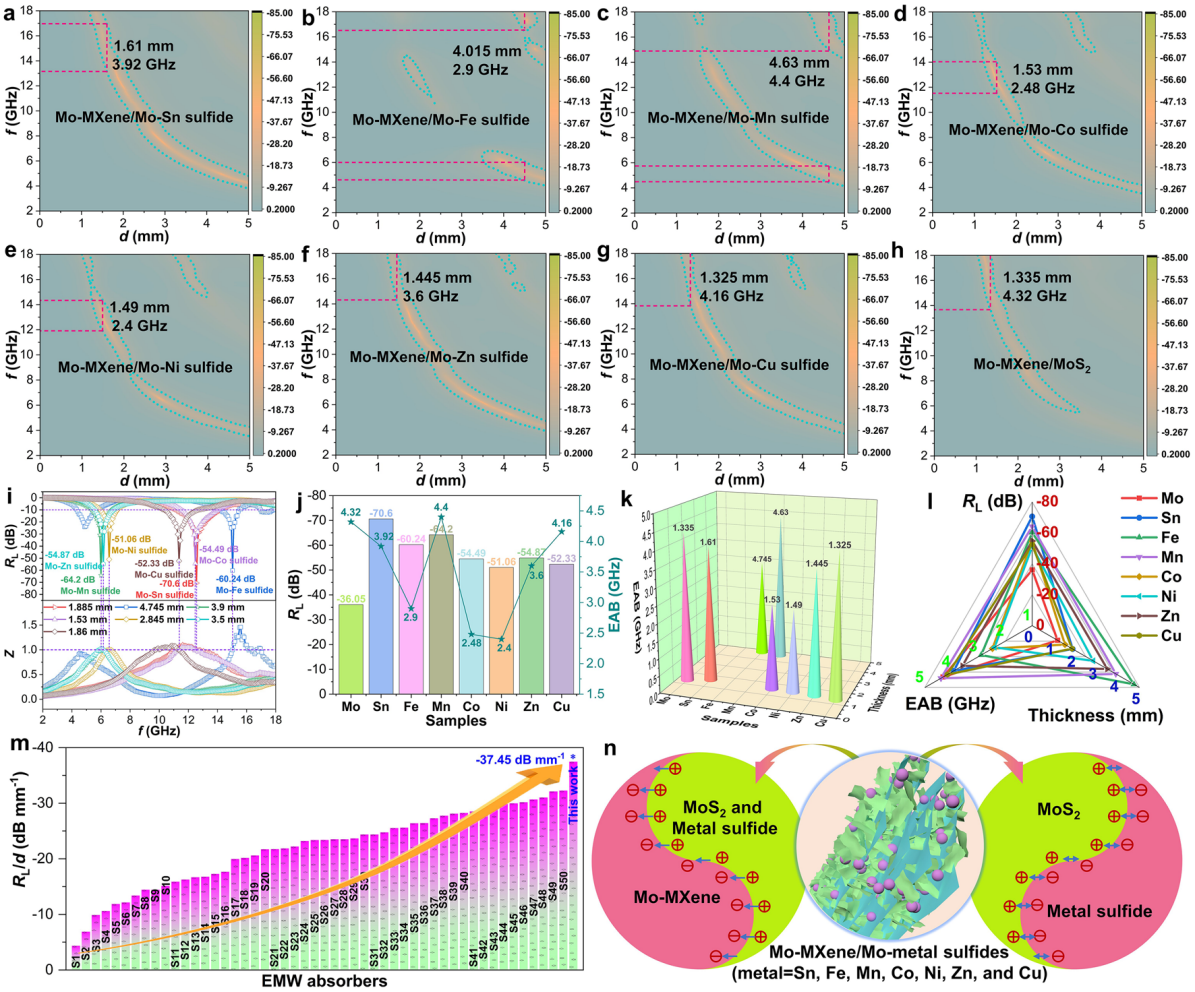
**a–h** 2D *R*_L_ plots. **i** Corresponding Z and *R*_L_ values. **j** Comparison of minimum reflection loss and EAB for the samples and **k** EAB for optimal thickness of all samples. **l** Performance comparison of all samples. **m** Comparison of *R*_L_/*d* of the MXene-based EMW absorbers (see Table S1 for details). **n** Schematic illustration of BIEF formation between Mo–MXene and metal sulfides

Based on earlier analyses of the Sn system, it is evident that the addition of Sn elements greatly enhances the EMW absorption performance of the material (Fig. 6a). Investigation of the reasons behind the outstanding performance of Mo–MXene/Mo–Sn sulfide reveals that the formation of a semiconductor–semiconductor–metal heterostructure is a key factor. This heterostructure contributes to Mo–MXene/Mo–Sn sulfide achieving an impressive *R*_L_ value of − 70.6 dB at a matching thickness of only 1.885 mm, and the EAB up to 3.92 GHz (Fig. 6i–k). To validate the applicability of this mechanism to other composites, the properties of Mo–MXene-based EMW absorption materials prepared under the same reaction conditions were explored. Specifically, different metal ions (Fe^3+^, Mn^2+^, Co^2+^, Ni^2+^, Zn^2+^, and Cu^2+^) were introduced to the reaction system to modulate electromagnetic parameters and EMW absorption properties. The resulting Mo–MXene/Mo–metal sulfides exhibit significantly improved EMW absorption capabilities (Fig. 6b–h) and impedance matching (Fig. 6i) compared to Mo–MXene/MoS_2_. In the Fe system, although the EAB of Mo–MXene/Mo–Fe sulfide is only 2.9 GHz, its *R*_L_ value is as high as − 60.24 dB, while the impedance matching is significantly improved (Figs. 6i, S31a, S32a, and S33a). According to the XRD results, this improvement may be due to the transition from Fe_0.95_S_1.05_ to Fe_7_S_8_ (Figs. S5b and S12b). On the other hand, when introducing metal ions like Co^2+^, Ni^2+^, Zn^2+^, and Cu^2+^, the *R*_L_ values are further enhanced to − 54.49, − 51.06, − 54.87, and − 52.33 dB, respectively (Fig. S31c–f and S32c–f). Notably, the EAB of Mo–MXene/Mo–Zn sulfide and Mo–MXene/Mo–Cu sulfide reach 3.6 and 4.16 GHz at smaller thicknesses (Fig. 6j, k). Mo–MXene/Mo–Mn sulfide displays a substantial performance increase of − 64.2 dB upon introducing the Mn element (Figs. S31b and S32b). Figure 6j, k also reveals that the EAB of Mo–MXene/Mo–Mn sulfide can reach 4.4 GHz. Further exploration of impedance matching indicates that the addition of these metal ions (Fe^3+^, Mn^2+^, Co^2+^, Ni^2+^, Zn^2+^, and Cu^2+^) results in good impedance matching, similar to the Sn system (Fig. S33). Figure S34 shows that the values of *d*_m_ and *f*_m_ for Mo–MXene/Mo–metal sulfides (metal = Fe, Mn, Co, Ni, Zn, and Cu) adhere to the quarter-wavelength cancellation theory, which confirms the superior absorption performance of Mo–MXene/Mo–metal sulfides. Therefore, the construction of semiconductor–semiconductor–metal phases, as described earlier, is applicable to other systems. It is evident that the unique BIEF constructed by both the semiconductor junction and the Schottky junction significantly enhances the EMW absorption performance. Furthermore, the presence of the 1T–2H phase produced by MoS_2_ may play an auxiliary role for Schottky junctions in semiconductor–metal mixed phases. Specifically, when Mo–MXene/Mo–metal sulfides are formed and 1T–2H MoS_2_ is present in the heterostructure, the *R*_L_ and EAB values of the material are significantly enhanced. Apparently, by incorporating different metal ions, optimal performance can be achieved in the C, X, or Ku bands, which is valuable for low and intermediate frequency absorption research. Figure 6l indicates that Mo–MXene/Mo–Sn sulfide and Mo–MXene/Mo–Mn sulfide exhibit the best performance based on a combination of *R*_L_, EAB, and matching thickness, surpassing the majority of MXene-based absorprion materials (Fig. 6m).

To assess the impact of non-formation of semiconductor–semiconductor–metal mixtures on absorption characteristics, we conducted EMW absorption studies on Mo–MXene/metal sulfides and metal sulfides (metal = Fe, Mn, Co, Ni, Zn, and Cu). Detailed information can be found in Figs. S35 and S36. Substituting Mo with Fe, Mn, Zn, or Cu led to poor EMW absorption properties for the resulting Mo–MXene/metal sulfides (Fig. S35a, b, e, f) or metal sulfides (Fig. S36a, b, e, f). Furthermore, Mo–MXene/metal sulfides and metal sulfides (metal = Co and Ni) exhibit exceptional properties (Figs. S35c, d, and S36c, d). To explain the exceptional behavior of the Co and Ni systems, we investigated their complex permittivity, complex permeability, and associated tangent values (Figs. S37–S39). In the Co and Ni systems, we observed the highest dielectric constants in Mo–MXene/Mo–Co sulfide and Mo–MXene/Mo–Ni sulfide (Fig. S37c, d). However, their high conductivity, as previously mentioned, results in strong reflection of EMW, which is detrimental to EMW absorption performance. Despite the *R*_L_ values of Mo–MXene/Mo–Co sulfide and Mo–MXene/Mo–Ni sulfide reaching − 54.49 and − 51.06 dB, their performance is similar to Mo–MXene/metal sulfides and metal sulfides (metal = Co and Ni), with only smaller matching thicknesses. This indicates that the formation of semiconductor–semiconductor–metal hybrid phases yields excellent reflection loss at small matching thicknesses, but the high dielectric loss capability hinders further performance enhancement and undesirable EAB (Fig. 6j, k). Moving on to the Fe, Mn, and Zn systems, we observed the highest permittivity in Mo–MXene/Mo–metal sulfide (metal = Fe, Mn, and Zn) (Fig. S37a, b, e). However, the EMW absorption capacity of Mo–MXene/metal sulfides and metal sulfides (metal = Fe, Mn, and Zn) is negligible due to their low permittivity, resulting in poor dielectric loss capability. Consequently, the high permittivity of Mo–MXene/Mo–metal sulfides (metal = Fe, Mn, and Zn) enhances their EMW absorption properties. Figure S37f indicates that the permittivity of the Cu system. Mo–MXene/Mo–Cu sulfide exhibits the best performance in this system.

The Cole–Cole curves of Mo–MXene/Mo–metal sulfides (metal = Fe, Mn, Co, Ni, Zn, and Cu) exhibit multiple semicircles and a straight line at the end, similar to those of Mo–MXene/Mo–Sn sulfide (Fig. S40). This suggests that all systems experience multiple polarization relaxations and conductive losses. Furthermore, the dielectric loss capability of Mo–MXene/Mo–metal sulfides is significantly enhanced compared to Mo–MXene/metal sulfides and metal sulfides (metal = Fe, Mn, Co, Ni, Zn, and Cu) (Figs. S40–S42). For the *α* values, the descending order is Mo–MXene/Mo–metal sulfides, Mo–MXene/metal sulfides, and metal sulfides (metal = Mn, Co, Ni, and Zn) (Fig. S43). Mo–MXene/Mo–metal sulfides exhibit stronger attenuation ability, which indicates that the construction of semiconductor–semiconductor–metal heterostructure effectively improves EMW absorption performance. In summary, significant performance improvement is achieved when semiconductor–semiconductor–metal hybrid phases are successfully constructed. This implies that the semiconductor–semiconductor–metal mixed phase should dominate over the semiconductor–metal mixed phase. In addition, the special interfacial BIEF formed by the semiconductor junction and the Schottky junction in the semiconductor–semiconductor–metal hybrid phase has a significant effect on enhancing the EMW absorption performance, as well as demonstrating that the 1T–2H phase generated by MoS_2_ itself acts as an auxiliary Schottky junction. As depicted in Fig. 6n, a BIEF is generated between the semiconductor and semiconductor mixed phases in Mo–MXene/Mo–metal sulfide, facilitating electron flow. Between the metal phase Mo–MXene and the semiconductor phase metal sulfide, the semiconductor’s energy bands bend at the interface to form a Schottky barrier. Electrons flow from metal sulfide to Mo–MXene, creating an electric field directed from metal sulfide to Mo–MXene. Schottky barrier formation also increases interface resistance, leading to charge accumulation. When EM waves are incident, the interface’s charge transforms the EMW energy into thermal energy through polarization behavior, improving EMW absorption performance.

### DFT Simulation and Absorption Mechanisms

**Fig. 7:**
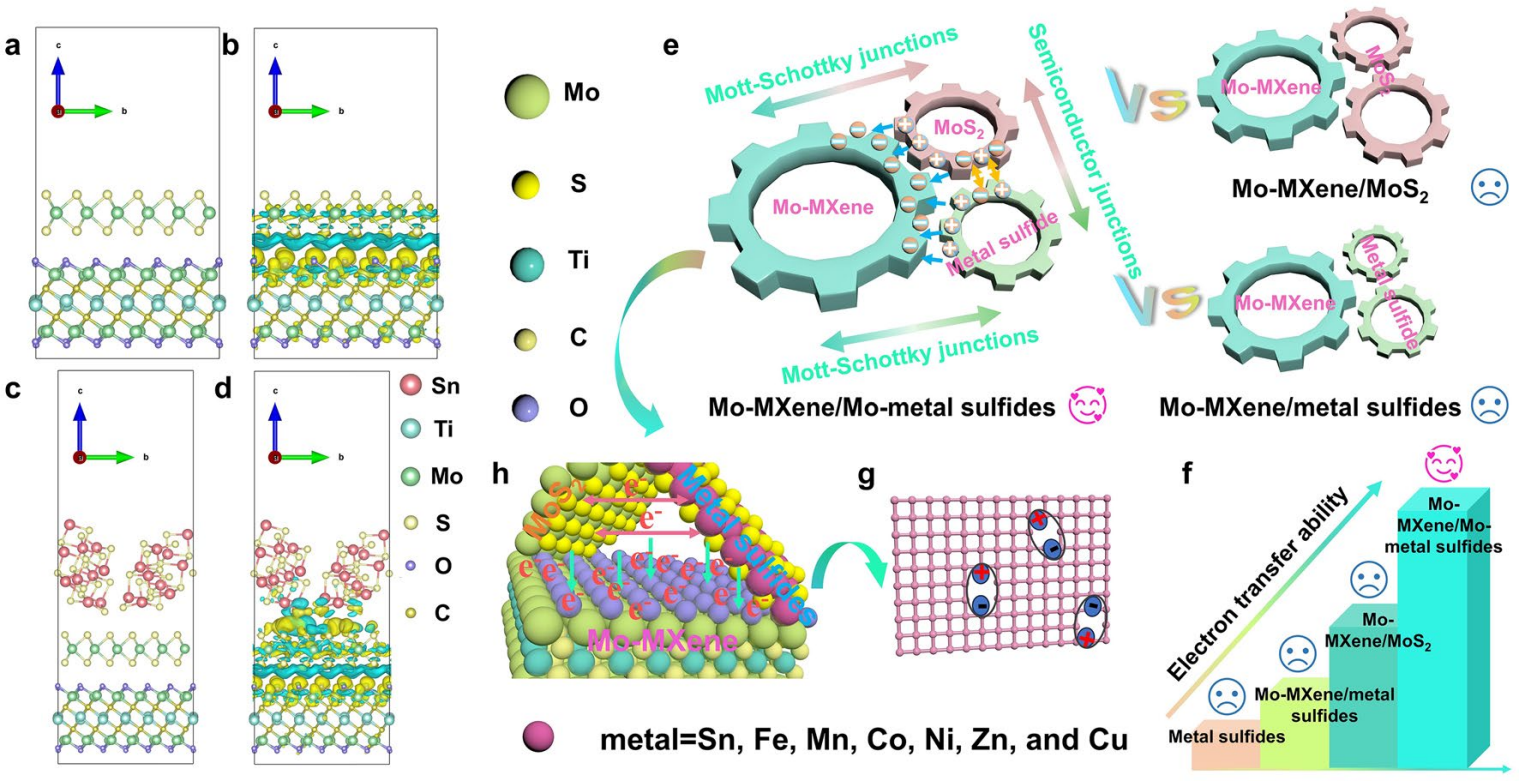
Differential charge density of **a, b** Mo–MXene/MoS_2_ and **c, d** Mo–MXene/Mo–Sn sulfide models, where the blue–green region represents electron consumption and the yellow region represents electron accumulation. **e–h** Schematic diagram of the EMW absorption mechanism for Mo–MXene/Mo–metal sulfide semiconductor–semiconductor–metal heterostructure

To theoretically verify the electron cloud distribution and BIEF effect in heterogeneous structures, differential charge density is calculated by density functional theory (DFT) simulation. The blue–green area represents electron consumption and the yellow area represents electron accumulation. The difference in charge density of the heterostructure model is shown in Figs. 7a, b, and S44a, b, where electron accumulation occurs around the Mo atom in Mo–MXene, while electron consumption occurs around MoS_2_. The corresponding differential charge-density map shows a strong and spontaneous electron migration from MoS_2_ to Mo–MXene. The ultrathin MoS_2_ nanosheet acts as a conductive platform, controlling the transfer of charge inside the heterogeneous structure. When Sn is doped, the differential charge density diagram shows that electrons flow from Sn_2_S_3_ to MoS_2_ and then to Mo–MXene (Figs. 7c, d, and S44c, d). This shows that at the semiconductor–semiconductor interface, electrons accumulate on MoS_2_. At the semiconductor–metal interface, electrons accumulate on Mo–MXene. According to these results, multiple BIEF can be formed in Mo–MXene/Mo–Sn sulfide. When semiconductor junctions and Mott–Schottky junctions are built in Mo–MXene, MoS_2_, and Sn_2_S_3_, the electron density of the total density of orbital states (TDOS) of Mo–MXene/Mo–Sn sulfides changes (Fig. S45) [59]. This indicates that the construction of BIEF helps to modulate the electron migration ability of Mo–MXene/Mo–Sn sulfides. This result validates the concept proposed above and also proves the electron transfer phenomenon found in XPS. In addition, the resulting BIEF can regulate the dielectric loss capacity and promote the internal stability of the heterogeneous structure. This is the key to understand the efficient absorption performance of the Mo–MXene/Mo–Sn sulfide heterostructure.

Based on the above results and analysis, the EMW absorption mechanism of Mo–MXene/Mo–metal sulfides is depicted in Fig. 7e–h. The dielectric loss model of Mo–MXene/Mo–metal sulfides is shown in Fig. 7e, where the three cogs of Mo–MXene, MoS_2_, and metal–sulfides are interlinked. When the semiconductor junction and the Mott–Schottky junction coexist, a unique BIEF is generated between the two, which affects the electron transfer capability and leads to the emergence of excellent dielectric loss (Fig. 7e, f). This constitutes the main EMW absorption mechanism of Mo–MXene/Mo–metal sulfides. As observed in Mo–MXene/Mo–metal sulfides, Mo–MXene/metal sulfides, and metal sulfides systems, MoS_2_ has high electrical conductivity and once formed, it can contribute to the conductive loss [61]. In an alternating magnetic field, both the surface functional groups of Mo–MXene and the surface defects of MoS_2_ serve as polarization centers, prompting dipoles to undergo polarization processes (Fig. 7g) [65, 66]. Furthermore, Mo–MXene/Mo–metal sulfides foster the creation of multiple interfaces, such as MoS_2_-metal sulfide (semiconductor–semiconductor), MoS_2_- Mo–MXene (semiconductor–metal), and metal sulfides- Mo–MXene (semiconductor–metal) (Fig. 7h), which enhances interfacial polarization. The BIEF constructed in the Mo–MXene/Mo–metal sulfide heterostructure exhibits changes in energy band structure, which enhances the oscillatory polarization relaxation of space charges in an alternating electric field environment [59]. Specifically, the discrepancy in Fermi energy levels between the semiconductor MoS_2_ and the semiconductor metal sulfides leads to the bending of semiconductor energy bands at the interface. This phenomenon induces electron flow between the semiconductor phases, generating a BIEF. At the interface, free electrons accumulate, forming a capacitor-like structure. Secondly, the energy difference between the metallic phase Mo–MXene and the semiconductor phases (MoS_2_ and metal sulfide) results in the formation of a Schottky barrier. Electrons flow from metal sulfides to Mo–MXene, creating an electric field directed from metal sulfides to Mo–MXene. These Schottky barriers also heighten interfacial resistance, leading to the accumulation of space charges. The influence of the incident EMW prompts the accumulated charges at both the semiconductor–semiconductor interface and the semiconductor–metal interface to convert EMW energy into thermal energy through polarization behavior. The abundant MoS_2_ nanosheets grown on the Mo–MXene precursor enhance the likelihood of multiple reflections and refractions of the incident EM waves. Additionally, the sample encompasses eddy current loss, natural resonance, and exchange resonance, all contributing to its magnetic loss capacity. Consequently, through the construction of semiconductor junctions and Mott–Schottky junctions between Mo–MXene, MoS_2_, and metal sulfides, the dielectric loss capability of Mo–MXene/Mo–metal sulfides has been significantly improved, which enhances the EMW absorption performance. The loss mechanisms that have the greatest impact on EMW attenuation is mainly the related polarization loss induced by BIEF [67]. Our findings emphasize that utilizing solely semiconductor–metal mixed phase or single phase materials results in poor EMW absorption performance. The successful construction of semiconductor–semiconductor–metal hybrid phases has a substantial positive impact on improving absorption performance.

## Conclusions

In summary, we introduce a novel strategy for the fabrication of high-performance EMW absorption materials through the construction of semiconductor–semiconductor–metal multi-heterostructures, including semiconductor junctions and Mott–Schottky junctions. The generation and polarization behavior of BIEF is shown to be a key factor in the improvement of EMW absorption properties, based on experimental results and theoretical calculations. Building upon this innovative strategy, we conducted a comparative assessment of all samples within the Sn system, uncovering that the successful realization of semiconductor–semiconductor–metal heterostructures leads to outstanding performance. As a remarkable result of this approach, Mo–MXene/Mo–Sn sulfide exhibits remarkable EMW absorption capabilities with a *R*_L_ value of − 70.6 dB at a matching thickness of only 1.885 mm, accompanied by an extensive EAB of up to 3.92 GHz. The correctness of the results was further verified by RCS simulation further confirms the possibility of practical application of Mo–MXene/Mo–Sn sulfide in anti-radar detection. Furthermore, our investigations have uncovered exceptional *R*_L_ values (< − 45.0 dB) at the C-band and X-band, with matching thicknesses of less than 5 mm. Moreover, this strategic approach can be extended to other MXene/Mo–metal sulfides (metal = Fe, Mn, Co, Ni, Zn, and Cu) through the selection of different metal ions. Consequently, this work not only establishes innovative guidelines for structural design but also introduces a fresh avenue for semiconductor materials research in the realm of EMW absorption. The successful integration of semiconductor–semiconductor–metal heterostructures presents an avenue to significantly enhance EMW absorption capabilities and paves the way for novel advancements in this field.

## Supplementary Information

Below is the link to the electronic supplementary material.

Supplementary file1 (PDF 8264 KB)
