# Supplementary material for: Constructing Built-In Electric Fields with Semiconductor Junctions and Schottky Junctions Based on Mo–MXene/Mo–Metal Sulfides for Electromagnetic Response
Source: Nanomicro Lett. 2024 Jun 11;16:213. doi: 10.1007/s40820-024-01449-7 (PMC11166625; doi:10.1007/s40820-024-01449-7)
Supplement: Supplementary file 1 — Supplementary file1 (PDF 8264 KB) [file 40820_2024_1449_MOESM1_ESM.pdf]

Supporting Information for

Constructing Built-In Electric Fields with Semiconductor Junctions and Schottky Junctions Based on Mo-MXene/Mo-Metal Sulfides for Electromagnetic Response

Xiaojun Zeng[1,#,*], Xiao Jiang[1,#] , Ya Ning[1], Yanfeng Gao[1, 2], Renchao Che[3, 4,*]

[1] School of Materials Science and Engineering, Jingdezhen Ceramic University, Jingdezhen 333403, P. R. China

[2] School of Materials Science and Engineering, Shanghai University, Shanghai 200444, P. R. China

[3] Laboratory of Advanced Materials, Shanghai Key Lab of Molecular Catalysis and Innovative Materials, Academy for Engineering & Technology, Fudan University, Shanghai 200438, P. R. China

[4] Zhejiang Laboratory, Hangzhou 311100, P. R. China

[#] Xiaojun Zeng and Xiao Jiang contributed equally to this work.

* Corresponding authors. E-mail: zengxiaojun@jcu.edu.cn (Xiaojun Zeng), rcche@fudan.edu.cn (Renchao Che)

S1 RCS Simulation Process

CST Studio Suite 2022 was employed to simulate the Radar Cross-Section (RCS) of MXene/Mo-metal sulfides (metal = Sn, Fe, Mn, Co, Ni, Zn, and Cu). Typically, the perfect electric conductor (PEC) model with a base area of 200 mm × 200 mm is established, while the PEC surface (200 mm × 200 mm) is coated with a wave absorption material. The model plate was placed on the XOY plane, the linearly polarized plane electromagnetic wave was incident from the positive direction of the Z axis to the negative direction of the Z axis, and the electric polarization direction propagates along the X axis. Open boundary conditions are set in all directions. The scattering direction is determined by theta and phi in spherical coordinates. RCS can be defined as:

$$\sigma\ (\mathrm{dB\ m^2}) = 10\log\{(4\pi S/\lambda^2)(|E_s/E_i|)\}^2$$

where S, λ, E_s, and E_i represent the area of the simulation model, the wavelength of electromagnetic wave, the electric field intensity of scattered wave and the incident wave, respectively.

S2 DFT Simulation Process

We utilized first-principles to conduct Spin-polarization density functional theory (DFT) calculations within the generalized gradient approximation (GGA) based on the Perdew-Burke-Ernzerhof (PBE) formulation [S1-S3]. The projected augmented wave (PAW) potentials were chosen to represent the ionic cores and consider valence electrons using a plane wave basis set with a kinetic energy cutoff of 450 Ev [S4, S5]. Van der Waals interactions were included using the DFT-D3 method developed by Grimme [S6, S7]. The electronic energy was deemed self-consistent if the energy change was less than 10^{-5} eV. A geometry optimization was deemed converged when the energy change was below 0.02 eV Å^{-1}. Throughout the relaxation process, a $1 \times 1 \times 1$ Gamma centered grid was employed in the Brillouin zone. A 15 Å vacuum layer was typically added to the surface to eliminate artificial interactions between periodic images. Spin-polarized calculations were carried out in this study.

S3 Supplementary Figures and Tables

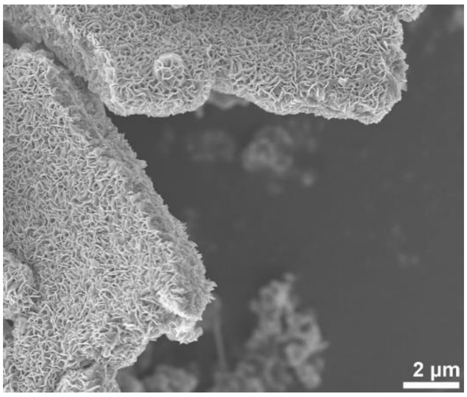

Fig. S1 SEM images of Mo-MXene/MoS_2

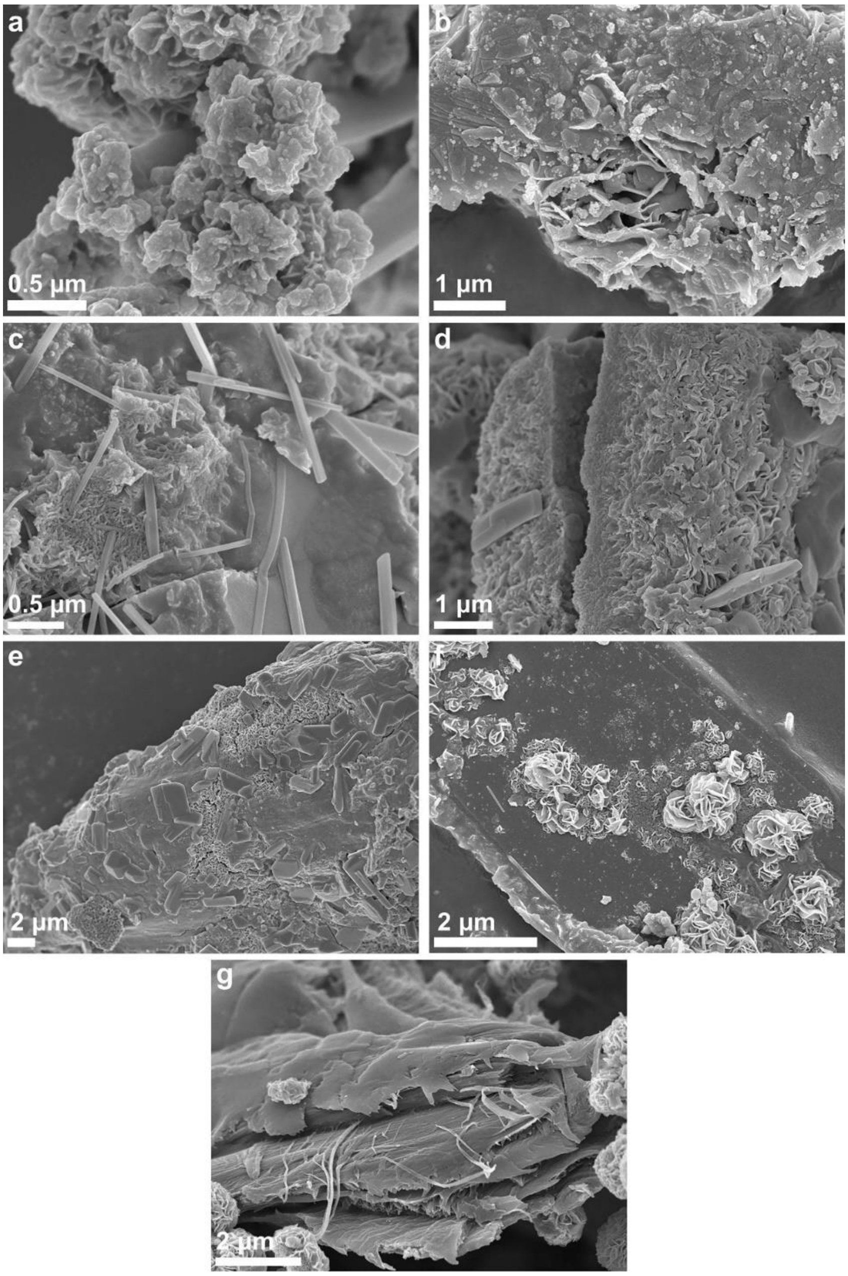

Fig. S2 SEM images of (**a**) Mo-MXene/Mo-Sn sulfide, (**b**) Mo-MXene/Mo-Fe sulfide, (**c**) Mo-MXene/Mo-Mn sulfide, (**d**) Mo-MXene/Mo-Co sulfide, (**e**) Mo-MXene/Mo-Ni sulfide, (**f**) Mo-MXene/Mo-Zn sulfide, and (**g**) Mo-MXene/Mo-Cu sulfide

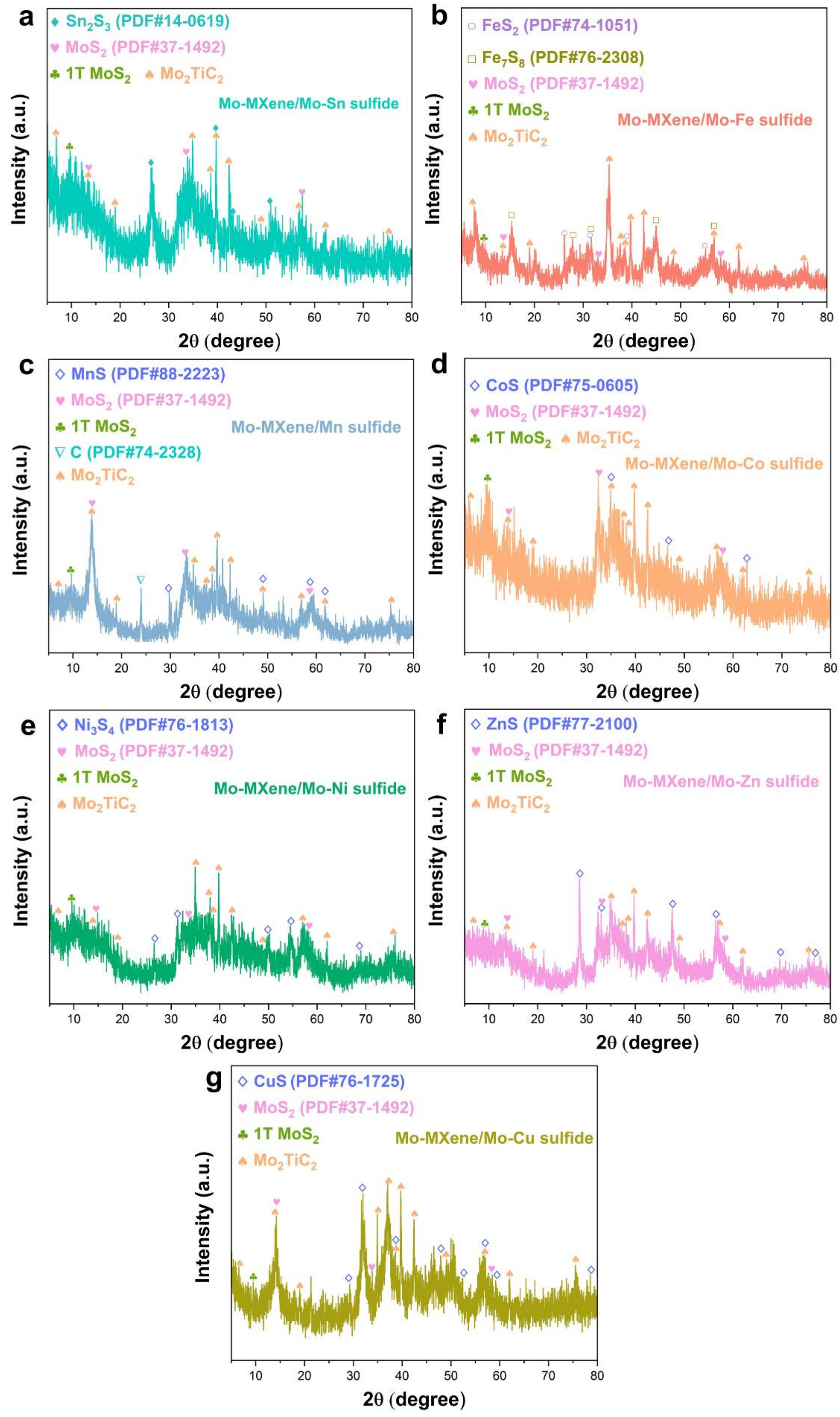

Fig. S3 XRD patterns of (**a**) Mo-MXene/Mo-Sn sulfide, (**b**) Mo-MXene/Mo-Fe sulfide, (**c**) Mo-MXene/Mo-Mn sulfide, (**d**) Mo-MXene/Mo-Co sulfide, (**e**) Mo-MXene/Mo-Ni sulfide, (**f**) Mo-MXene/Mo-Zn sulfide, and (**g**) Mo-MXene/Mo-Cu sulfide

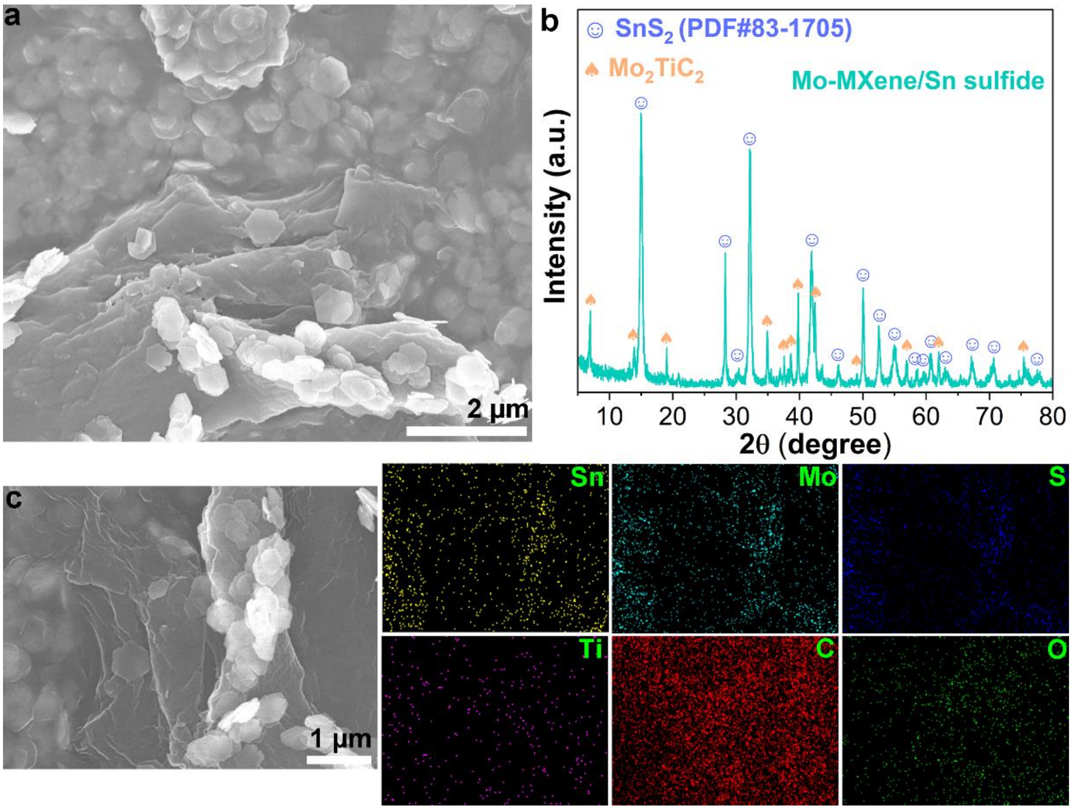

Fig. S4 (**a**) SEM image, (**b**) XRD pattern, and (**c**) EDS mapping image of Mo-MXene/Sn sulfide

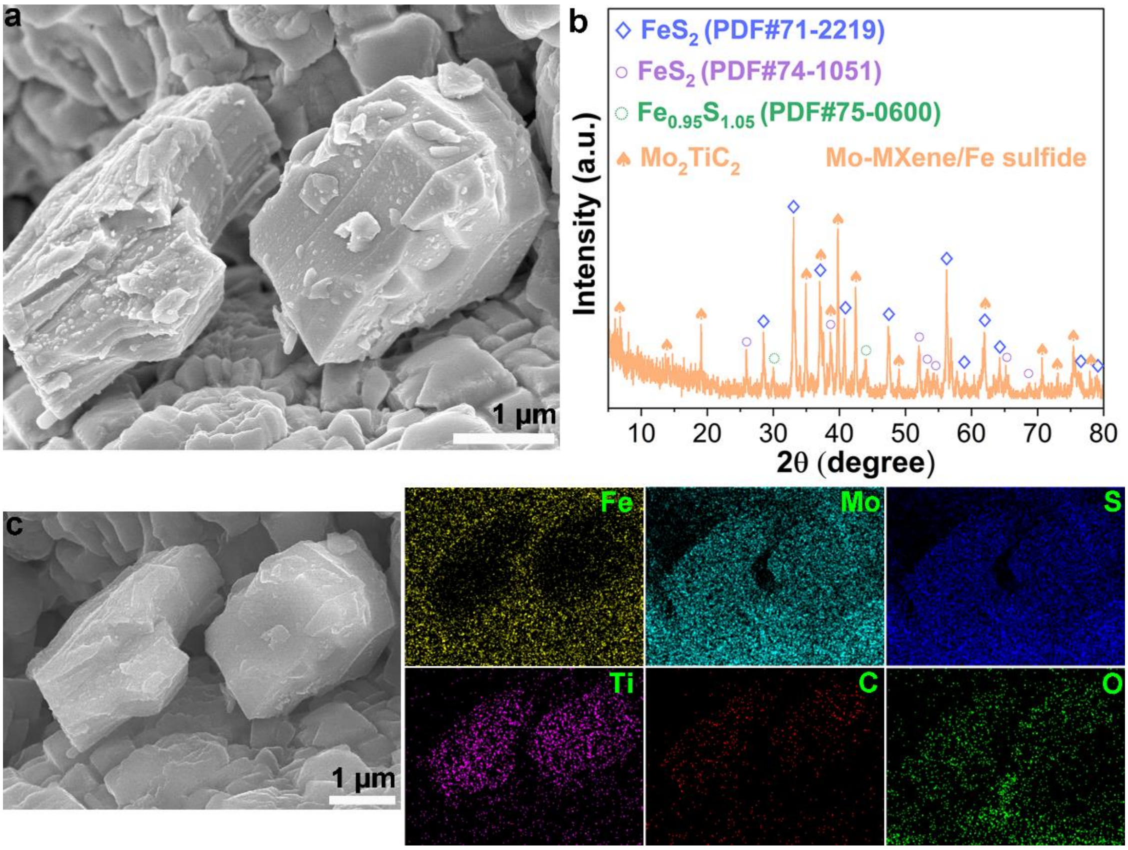

Fig. S5 (**a**) SEM image, (**b**) XRD pattern, and (**c**) EDS mapping image of Mo-MXene/Fe sulfide

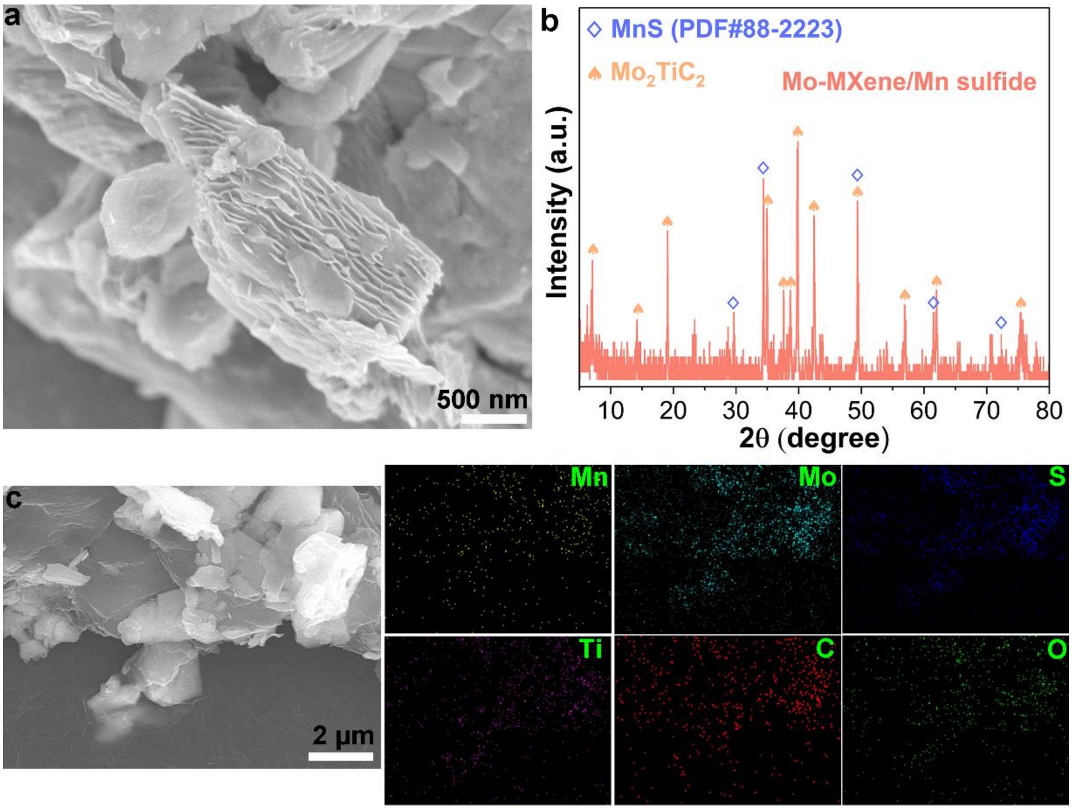

Fig. S6 (**a**) SEM image, (**b**) XRD pattern, and (**c**) EDS mapping image of Mo-MXene/Mn sulfide

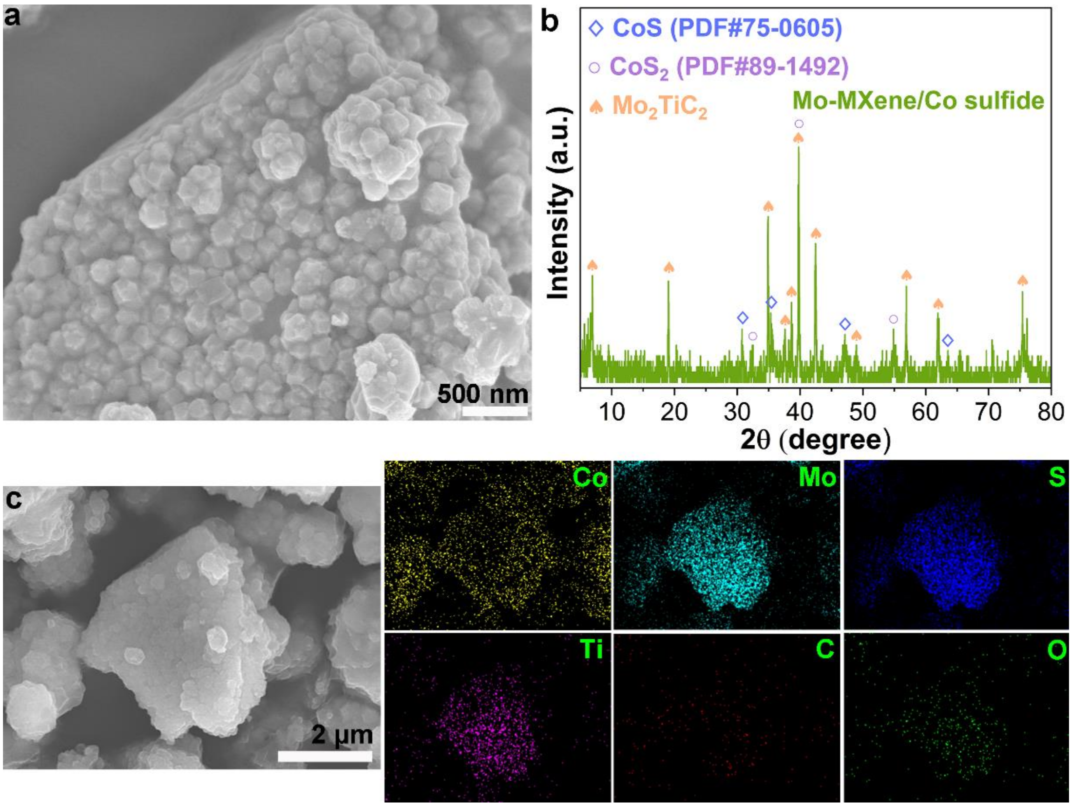

Fig. S7 (**a**) SEM image, (**b**) XRD pattern, and (**c**) EDS mapping image of Mo-MXene/Co sulfide

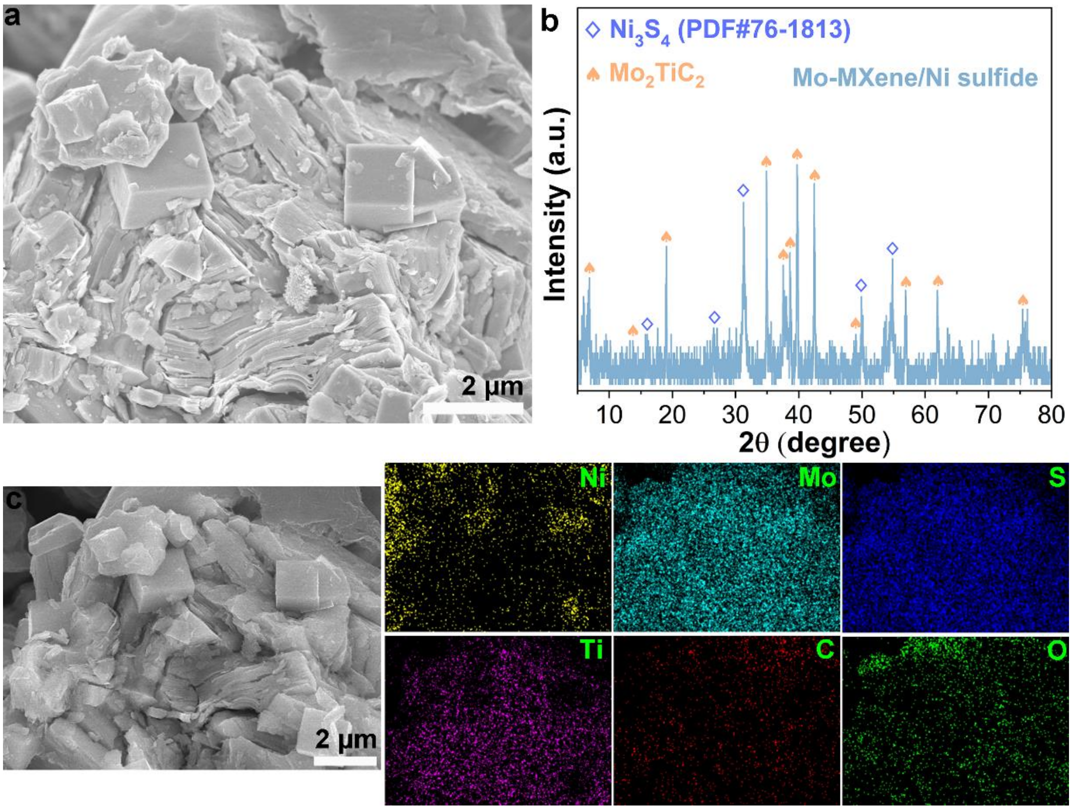

Fig. S8 (**a**) SEM image, (**b**) XRD pattern, and (**c**) EDS mapping image of Mo-MXene/Ni sulfide

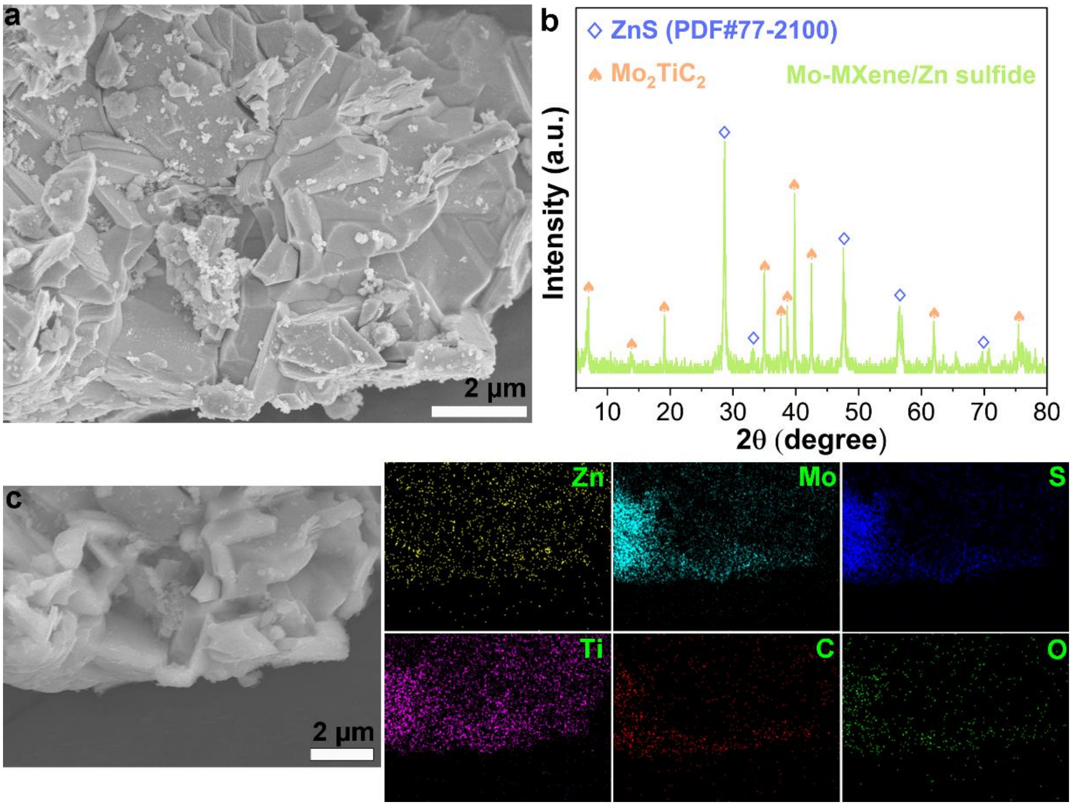

Fig. S9 (**a**) SEM image, (**b**) XRD pattern, and (**c**) EDS mapping image of Mo-MXene/Zn sulfide

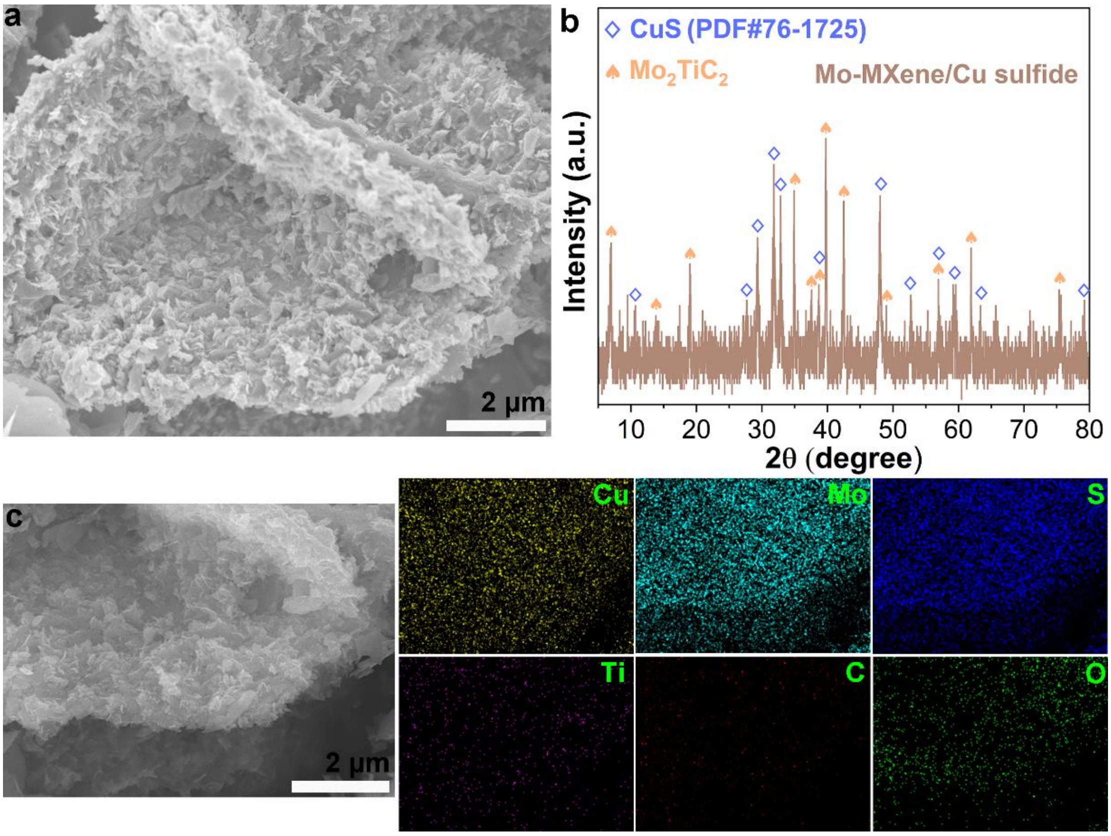

Fig. S10 (**a**) SEM image, (**b**) XRD pattern, and (**c**) EDS mapping image of Mo-MXene/Cu sulfide

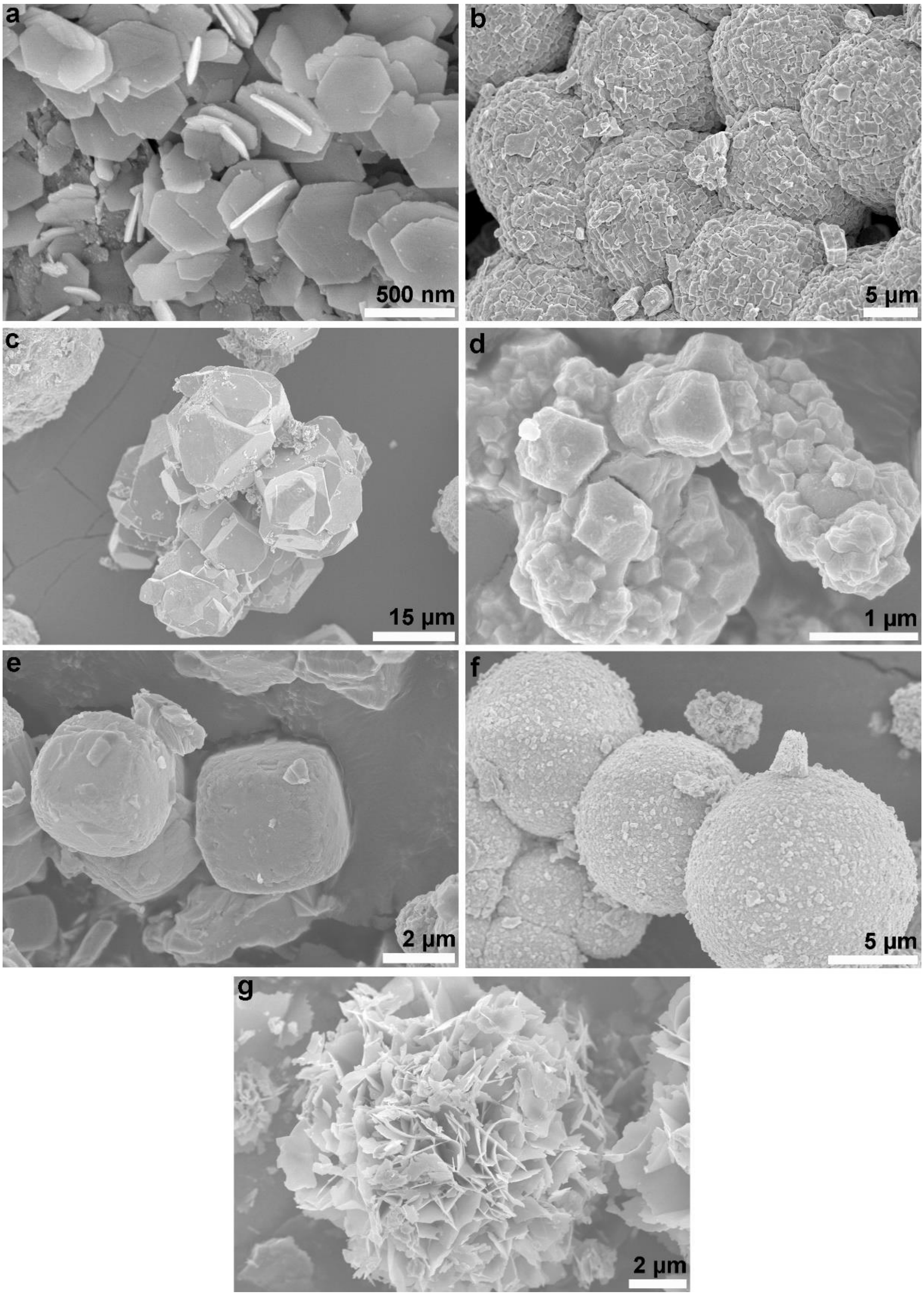

Fig. S11 SEM images for (**a**) Sn sulfide, (**b**) Fe sulfide, (**c**) Mn sulfide, (**d**) Co sulfide, (**e**) Ni sulfide, (**f**) Zn sulfide, and (**g**) Cu sulfide

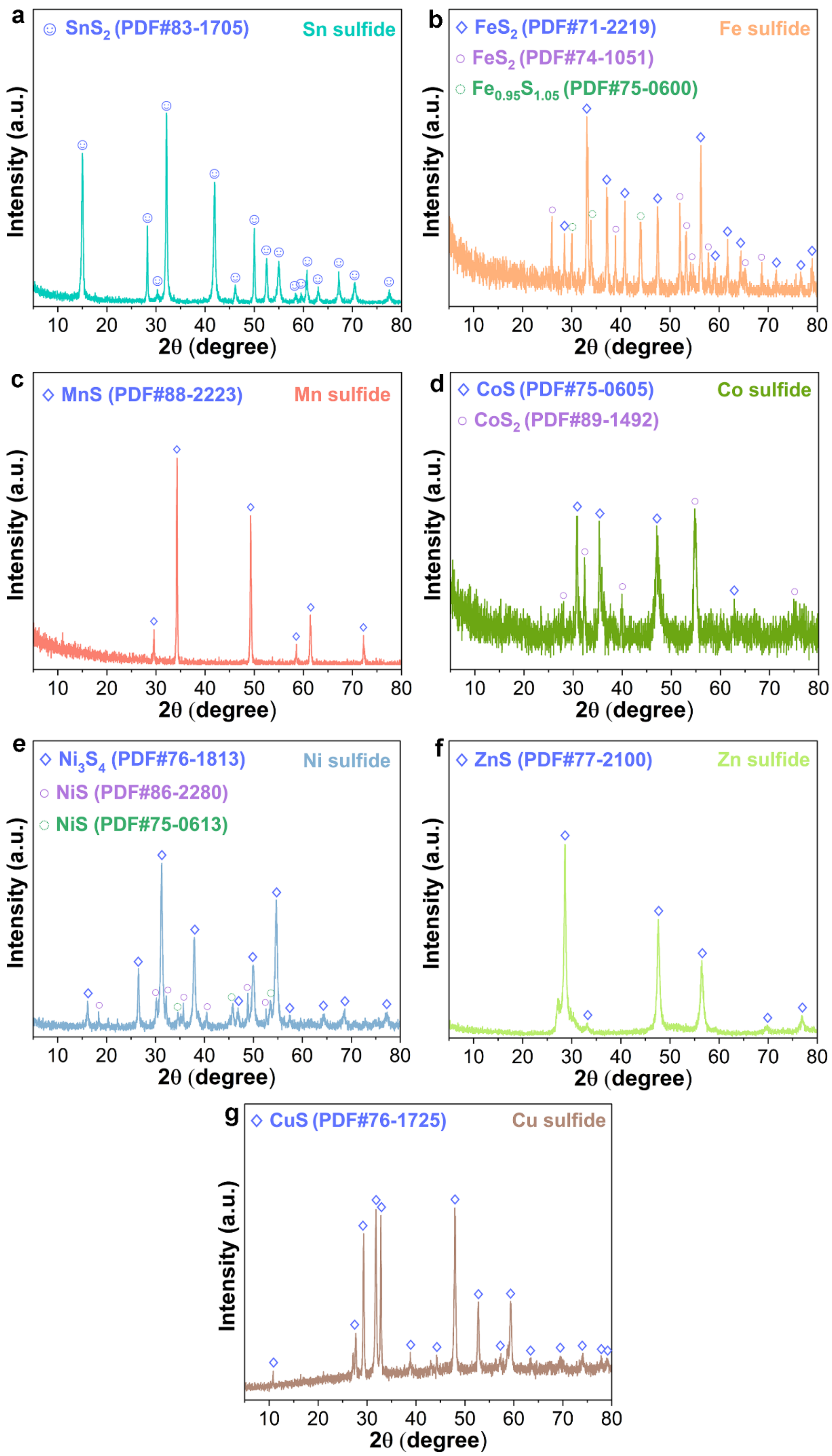

Fig. S12 XRD patterns of (**a**) Sn sulfide, (**b**) Fe sulfide, (**c**) Mn sulfide, (**d**) Co sulfide, (**e**) Ni sulfide, (**f**) Zn sulfide, and (**g**) Cu sulfide

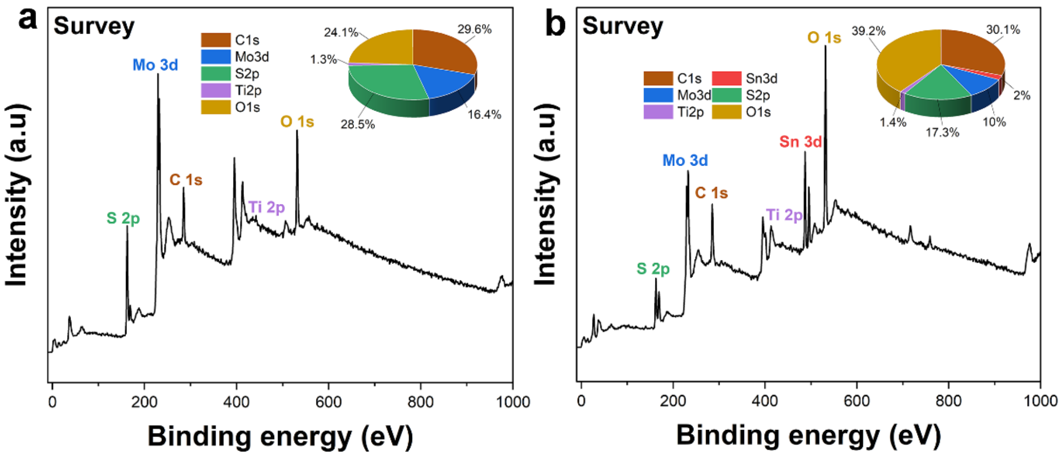

Fig. S13 XPS survey spectra of (**a**) Mo-MXene/Mo-Sn sulfide and (**b**) Mo-MXene/MoS_2

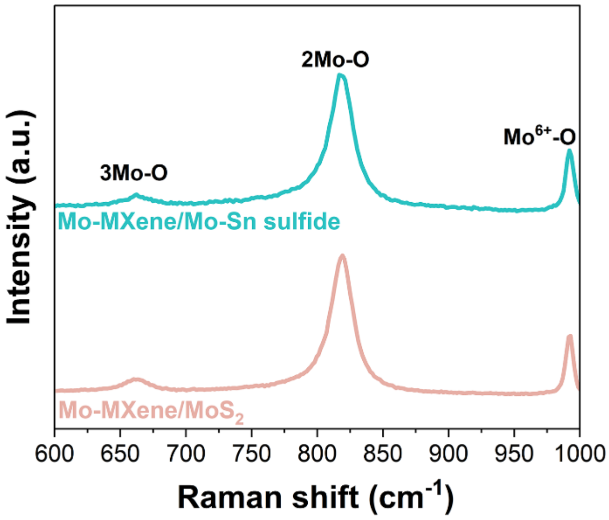

Fig. S14 Raman spectra of Mo-MXene/Mo-Sn sulfide and Mo-MXene/MoS_2

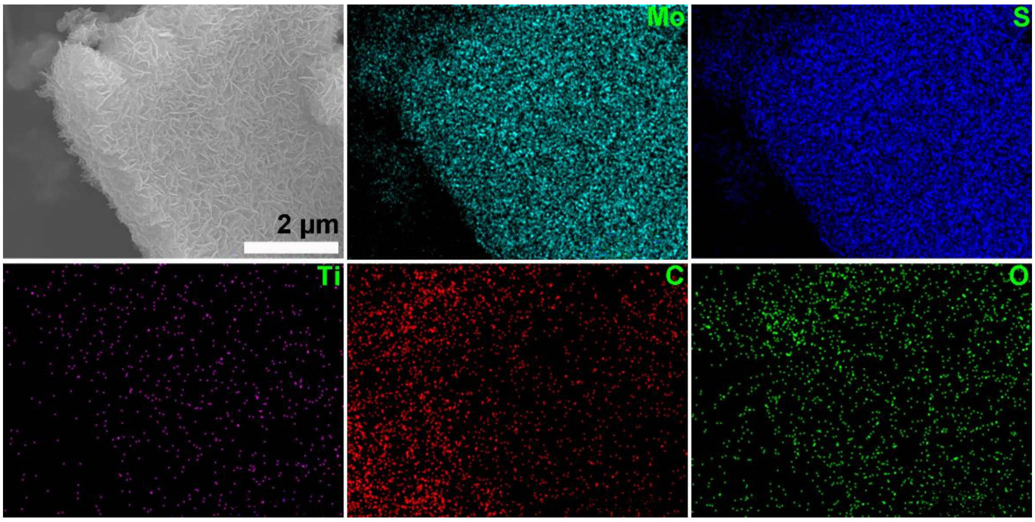

Fig. S15 EDS elemental mapping images of Mo-MXene/MoS_2

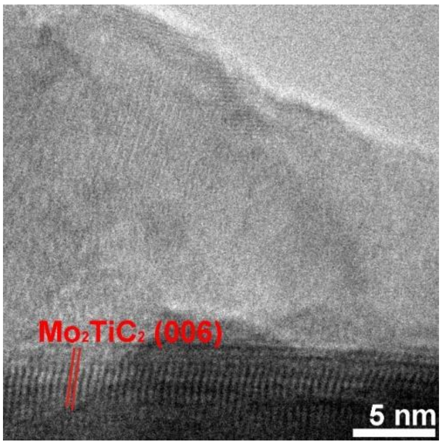

Fig. S16 HRTEM images of Mo-MXene/MoS_2

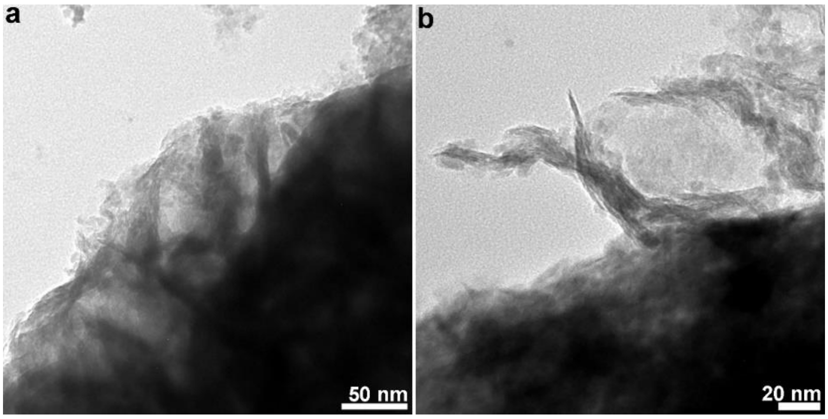

Fig. S17 TEM images of Mo-MXene/Mo-Sn sulfide

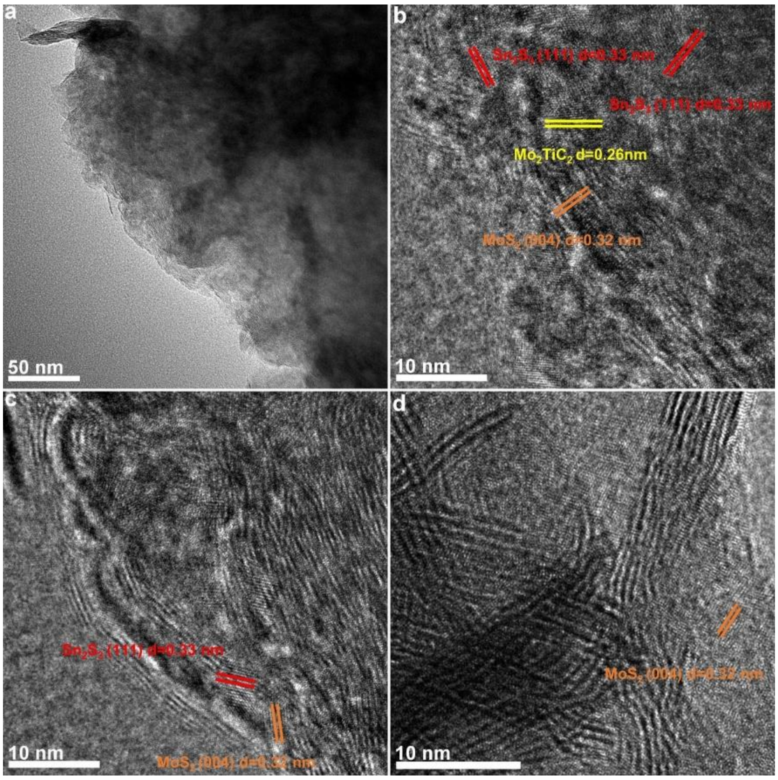

Fig. S18 (**a**) TEM and (**b-d**) HRTEM images of Mo-MXene/Mo-Sn sulfide

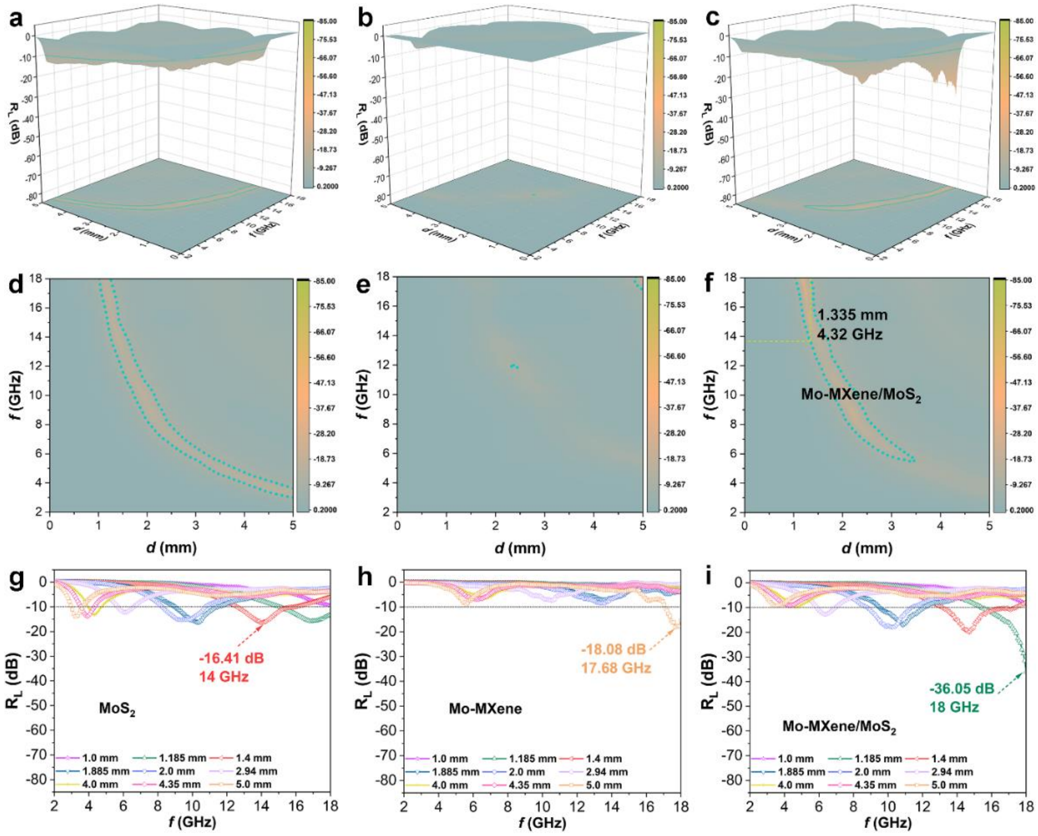

Fig. S19 (**a-c**) 3D R_L values, (**d-f**) 2D contour maps, and (**g-i**) 2D R_L values of (**a, d, g**) MoS_2, (**b, e, h**) Mo-MXene, and (**c, f, i**) Mo-MXene/MoS_2

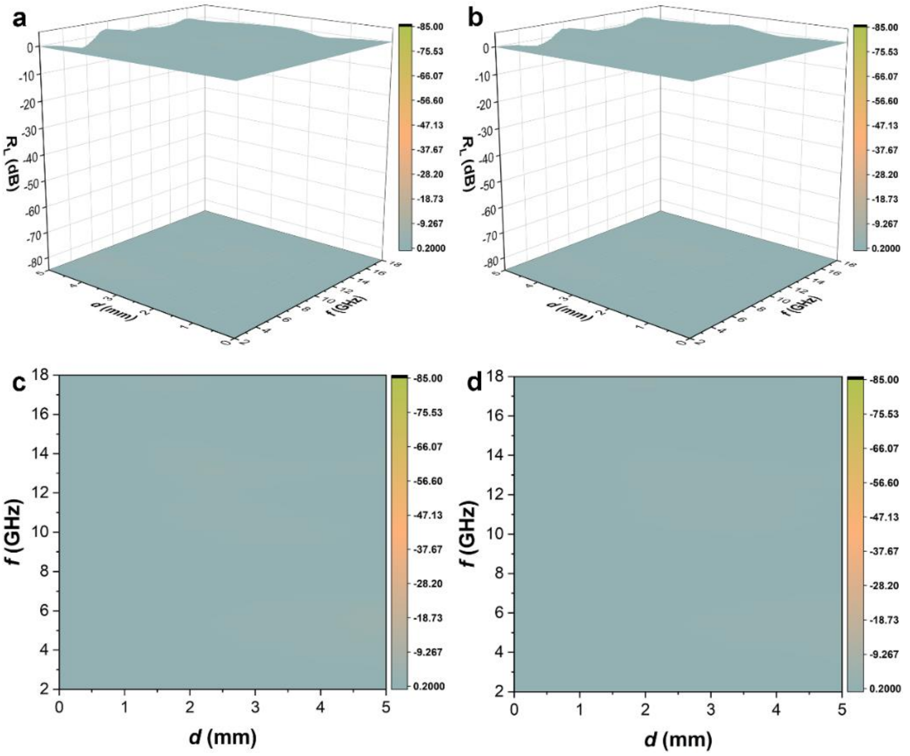

Fig. S20 (**a, b**) 3D R_L values and (**c, d**) 2D contour maps of (**a, c**) Mo-MXene/Sn sulfide and (**b, d**) Sn sulfide

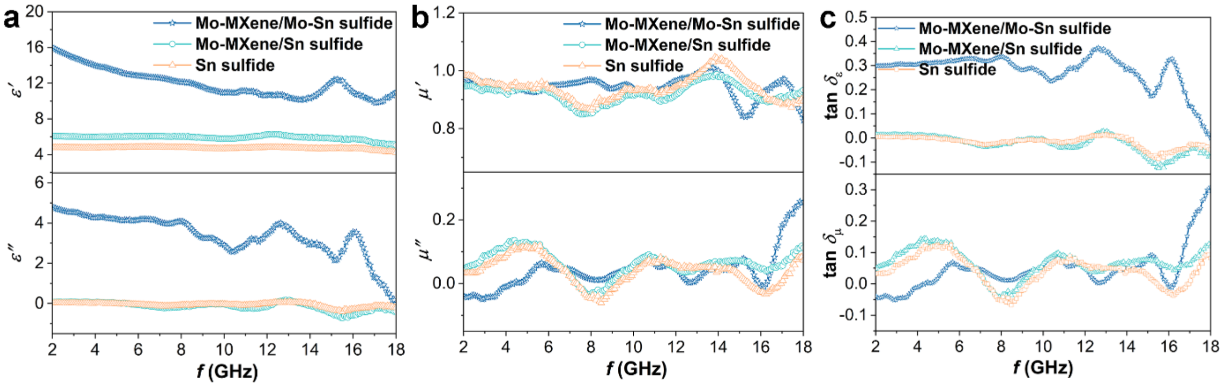

Fig. S21 (a) Permittivity, (b) permeability, and (c) tan δ_ε and tan δ_μ values of Mo-MXene/Mo-Sn sulfide, Mo-MXene/Sn sulfide, and Sn sulfide

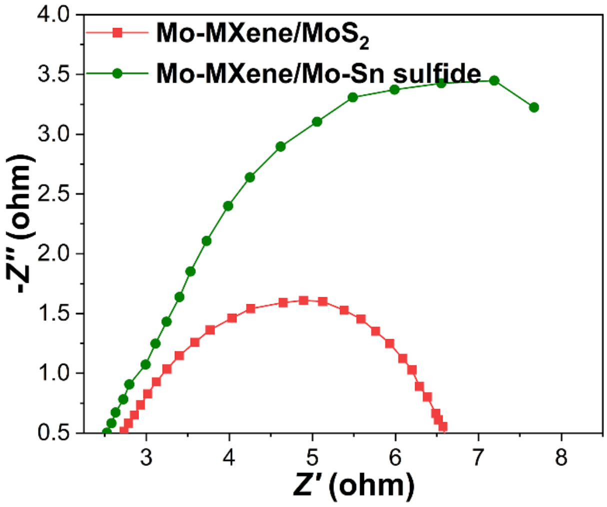

Fig. S22 Nyquist plots of Mo-MXene/MoS_2 and Mo-MXene/Mo-Sn sulfide

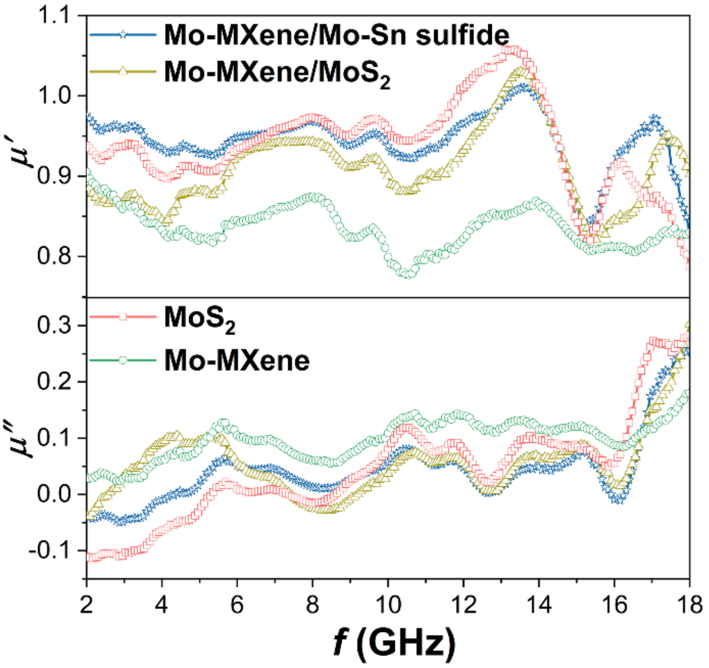

Fig. S23 Permeability of MoS_2, Mo-MXene, Mo-MXene/MoS_2, and Mo-MXene/Mo-Sn sulfide

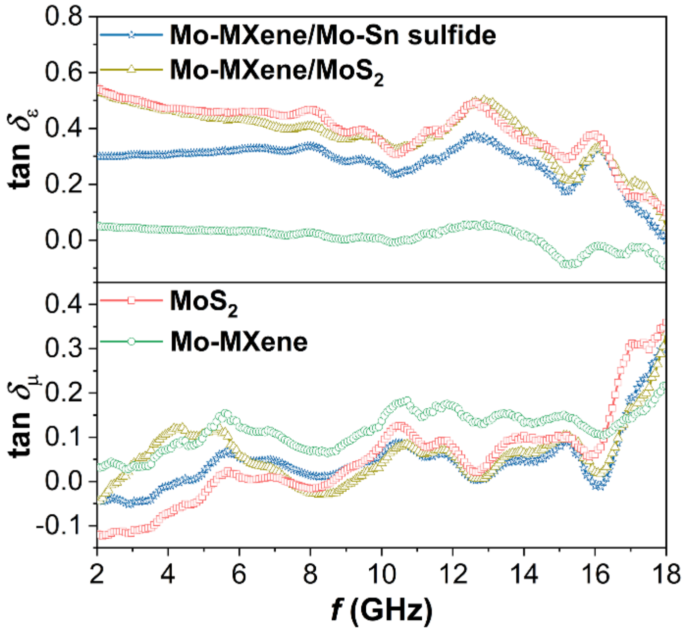

Fig. S24 tan δ_ε and tan δ_μ values of MoS_2, Mo-MXene, Mo-MXene/MoS_2, and Mo-MXene/Mo-Sn sulfide

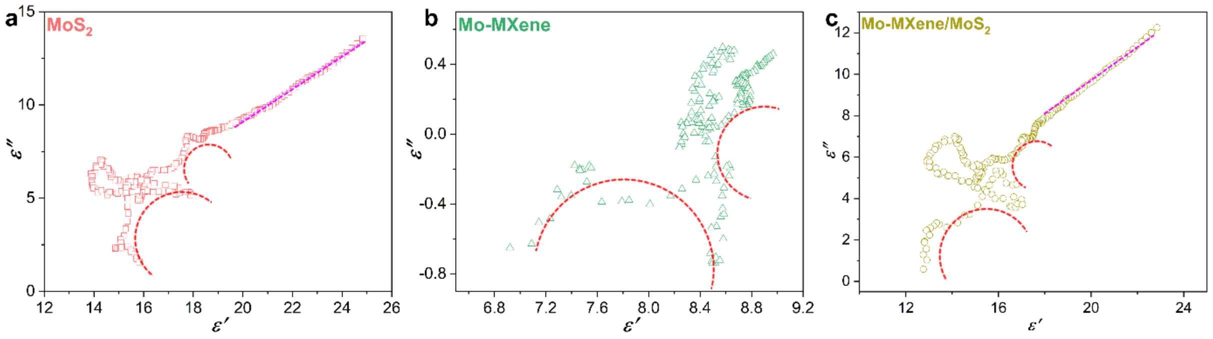

Fig. S25 ε'-ε'' curves of (**a**) MoS_2, (**b**) Mo-MXene, and (**c**) Mo-MXene/MoS_2

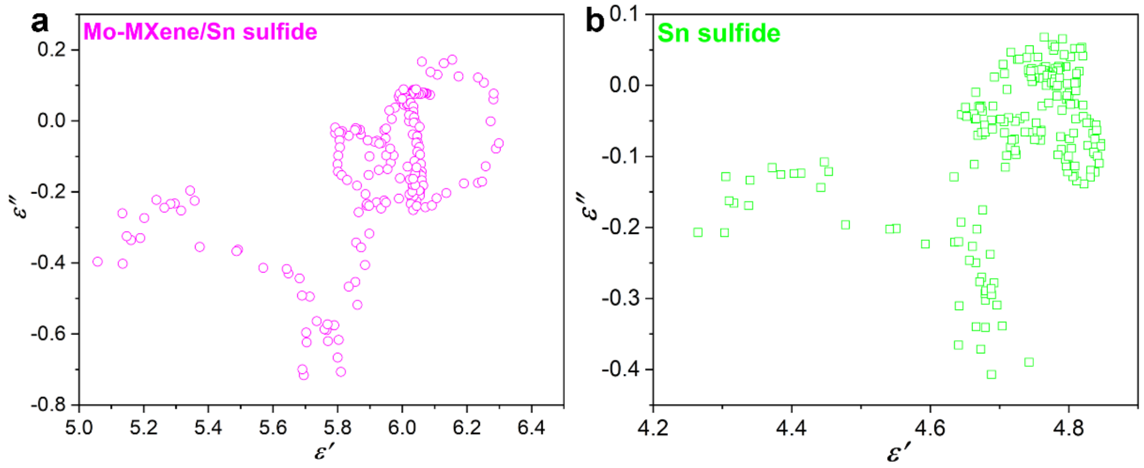

Fig. S26 ε'-ε'' curves of (**a**) Mo-MXene/Sn sulfide and (**b**) Sn sulfide

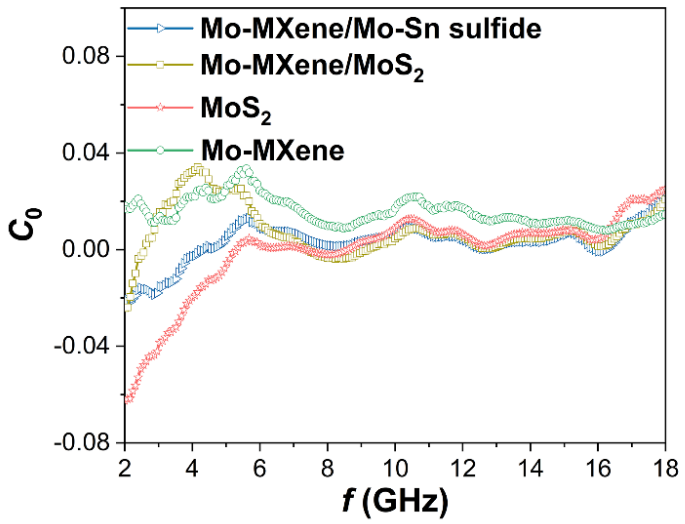

Fig. S27 C_0 values of MoS_2, Mo-MXene, Mo-MXene/MoS_2, and Mo-MXene/Mo-Sn sulfide

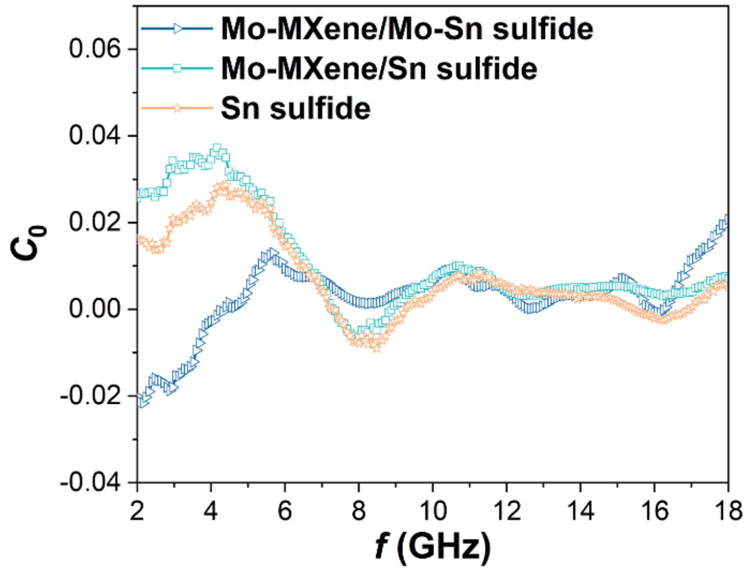

Fig. S28 C_0 values of Mo-MXene/Mo-Sn sulfide, Mo-MXene/Sn sulfide, and Sn sulfide

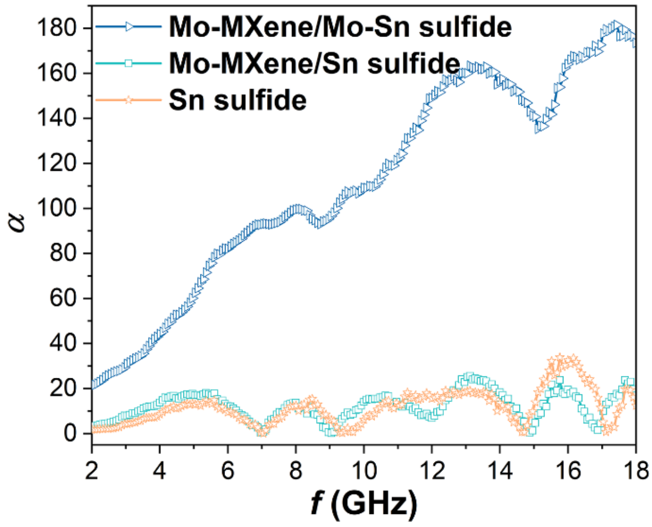

Fig. S29 α values of Mo-MXene/Mo-Sn sulfide, Mo-MXene/Sn sulfide, and Sn sulfide

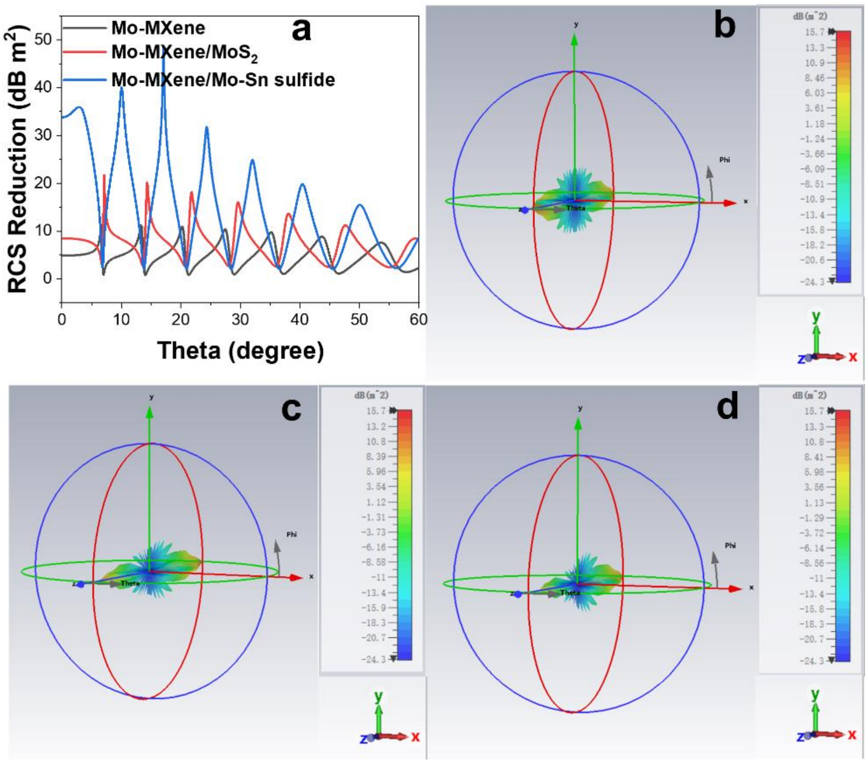

Fig. S30 (**a**) RCS reduction values of Mo-MXene, Mo-MXene/MoS_2, and Mo-MXene/Mo-Sn sulfide. CST simulation results of (**b**) PEC, PEC covered with (**c**) Mo-MXene, and (**d**) Mo-MXene/MoS_2

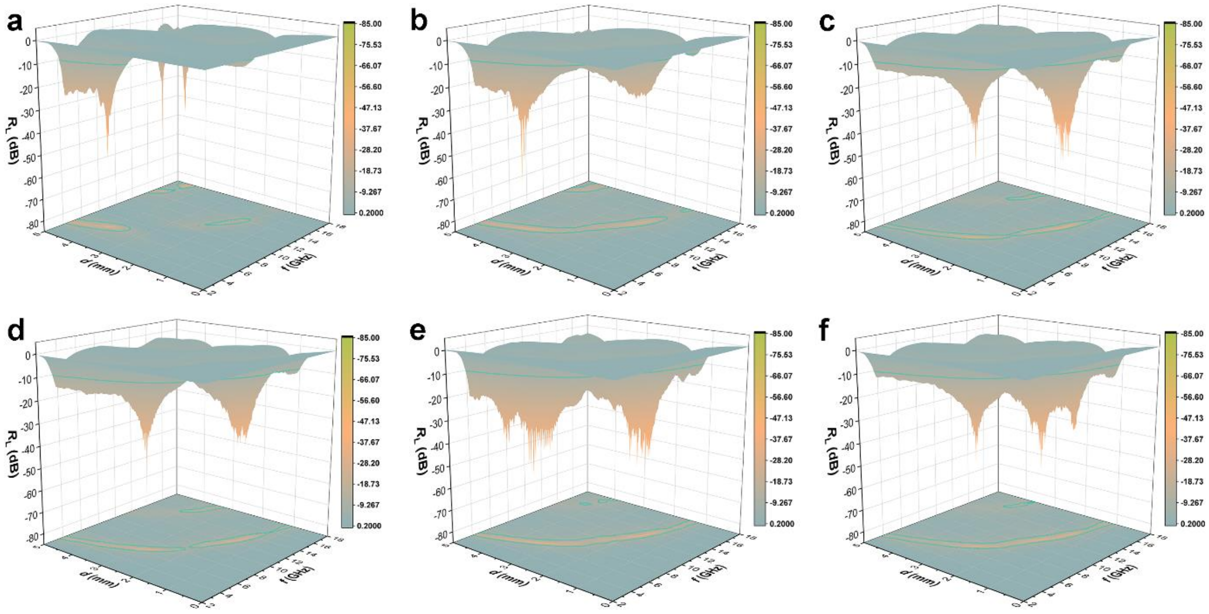

Fig. S31 3D R_L values of (**a**) Mo-MXene/Mo-Fe sulfide, (**b**) Mo-MXene/Mo-Mn sulfide, (**c**) Mo-MXene/Mo-Co sulfide, (**d**) Mo-MXene/Mo-Ni sulfide, (**e**) Mo-MXene/Mo-Zn sulfide, and (**f**) Mo-MXene/Mo-Cu sulfide

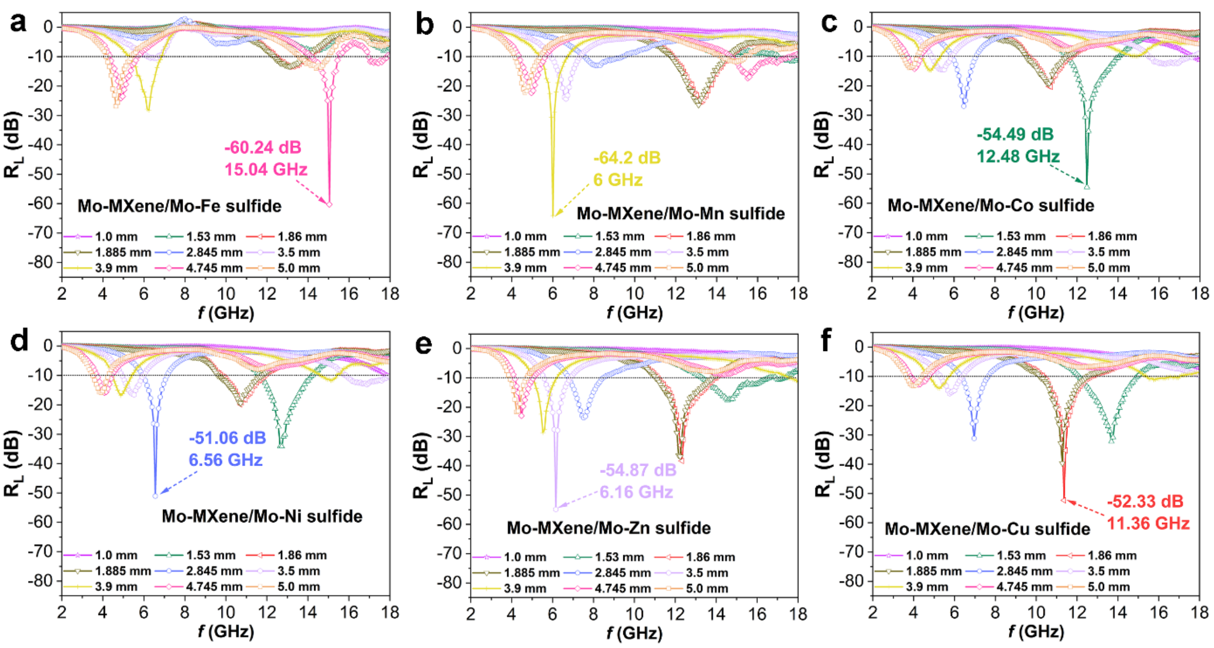

Fig. S32 2D R_L values of (**a**) Mo-MXene/Mo-Fe sulfide, (**b**) Mo-MXene/Mo-Mn sulfide, (**c**) Mo-MXene/Mo-Co sulfide, (**d**) Mo-MXene/Mo-Ni sulfide, (**e**) Mo-MXene/Mo-Zn sulfide, and (**f**) Mo-MXene/Mo-Cu sulfide

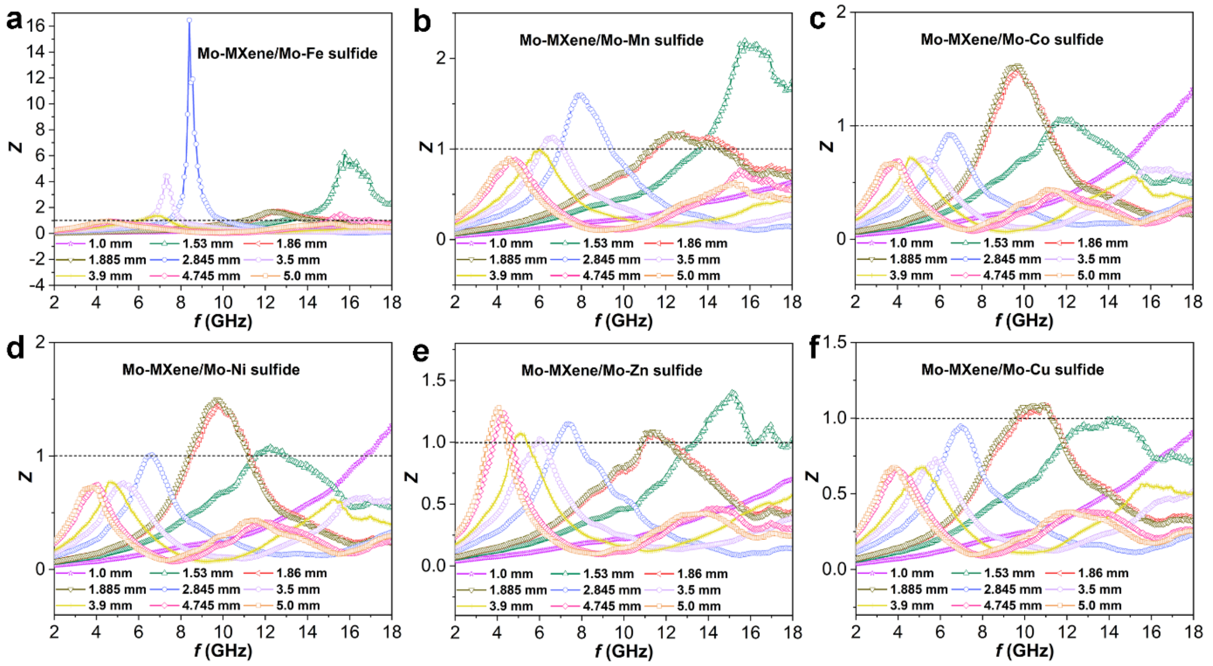

Fig. S33 *Z* values of (**a**) Mo-MXene/Mo-Fe sulfide, (**b**) Mo-MXene/Mo-Mn sulfide, (**c**) Mo-MXene/Mo-Co sulfide, (**d**) Mo-MXene/Mo-Ni sulfide, (**e**) Mo-MXene/Mo-Zn sulfide, and (**f**) Mo-MXene/Mo-Cu sulfide

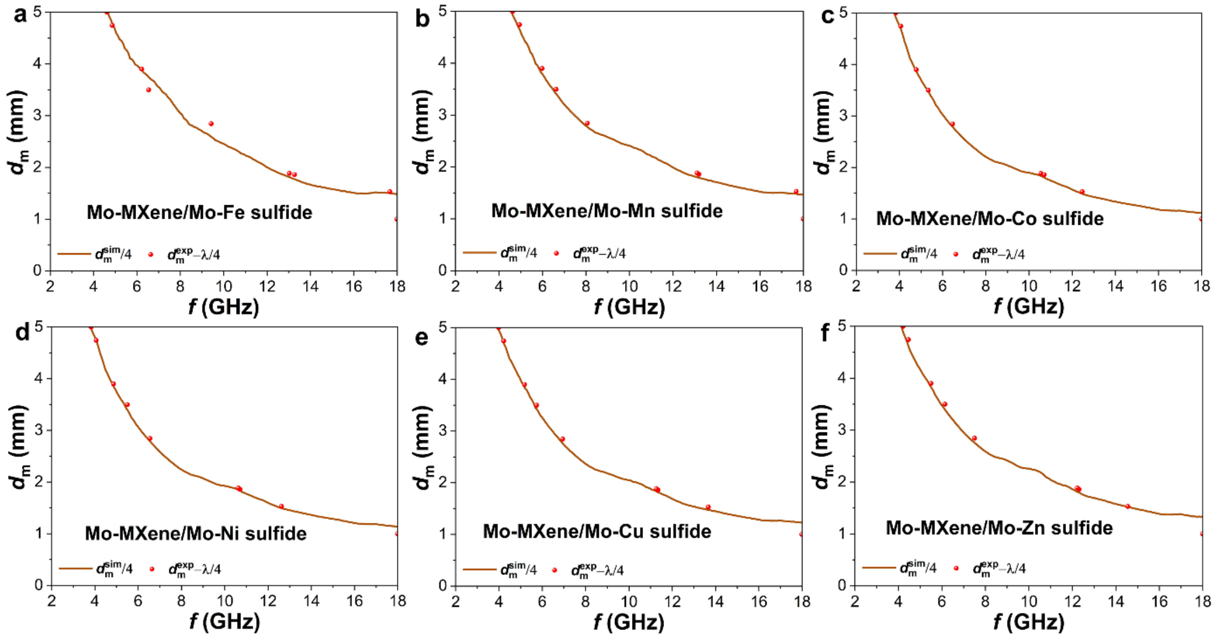

Fig. S34 Simulation of d_m (d_m^{sim}) vs. f_m curves for (**a**) Mo-MXene/Mo-Fe sulfide, (**b**) Mo-MXene/Mo-Mn sulfide, (**c**) Mo-MXene/Mo-Co sulfide, (**d**) Mo-MXene/Mo-N sulfide, (**e**) Mo-MXene/Mo-Zn sulfide, and (**f**) Mo-MXene/Mo-Cu sulfide

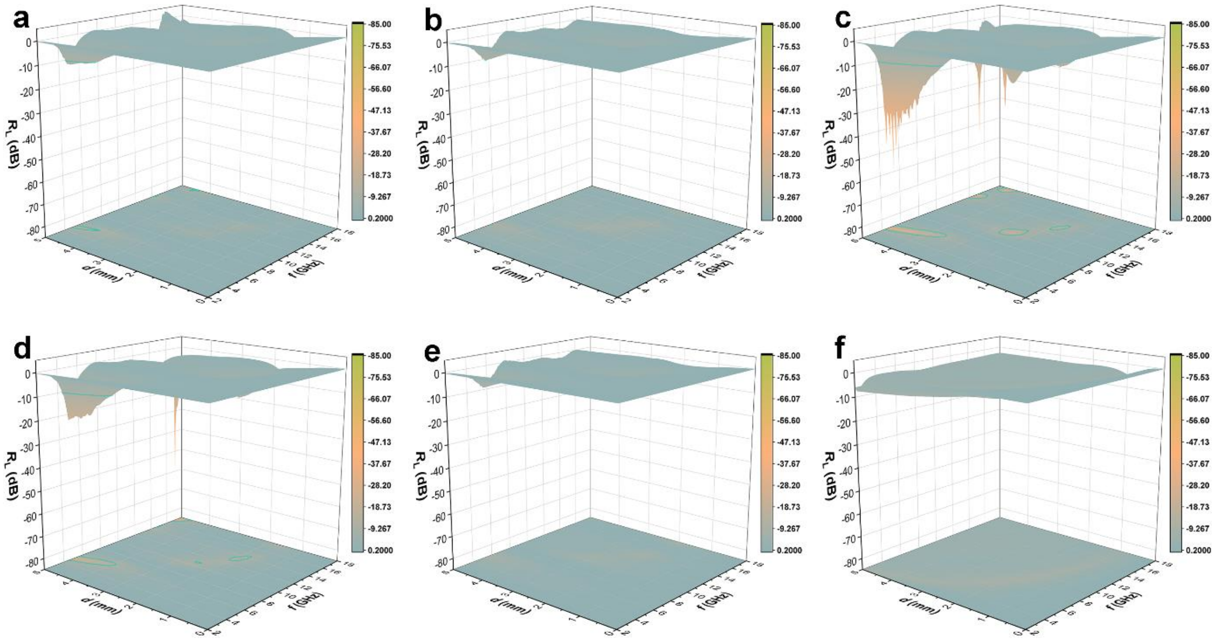

Fig. S35 3D R_L values of (**a**) Mo-MXene/Fe sulfide, (**b**) Mo-MXene/Mn sulfide, (**c**) Mo-MXene/Co sulfide, (**d**) Mo-MXene/Ni sulfide, (**e**) Mo-MXene/Zn sulfide, and (**f**) Mo-MXene/Cu sulfide

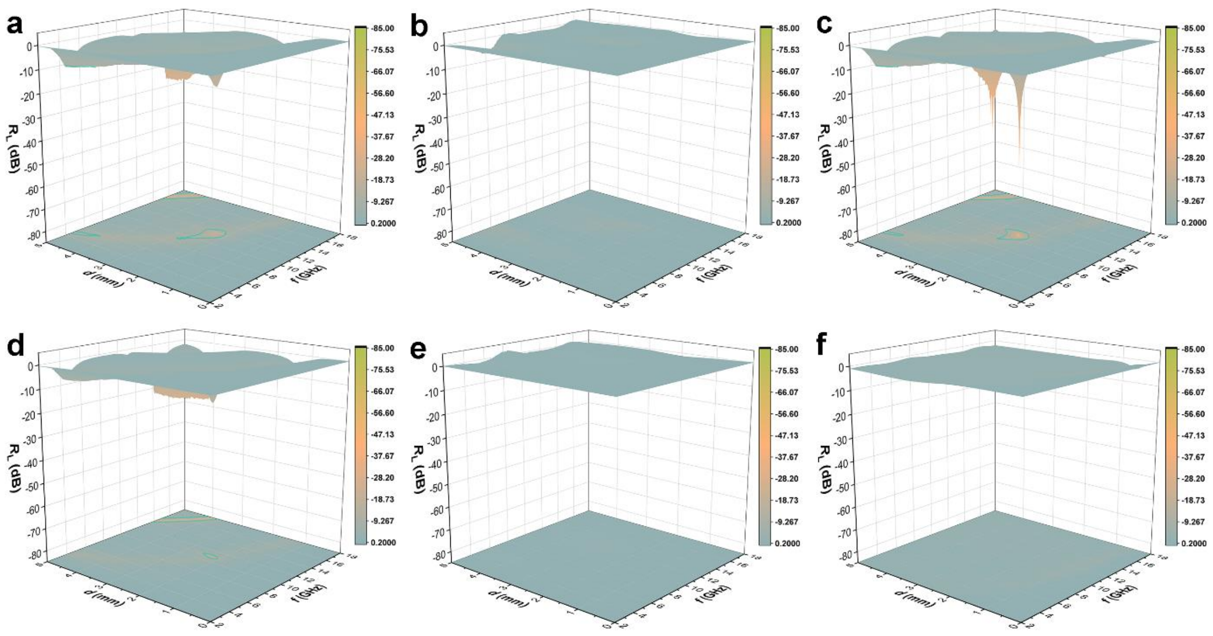

Fig. S36 3D R_L values of (**a**) Fe sulfide, (**b**) Mn sulfide, (**c**) Co sulfide, (**d**) Ni sulfide, (**e**) Zn sulfide, and (**f**) Cu sulfide

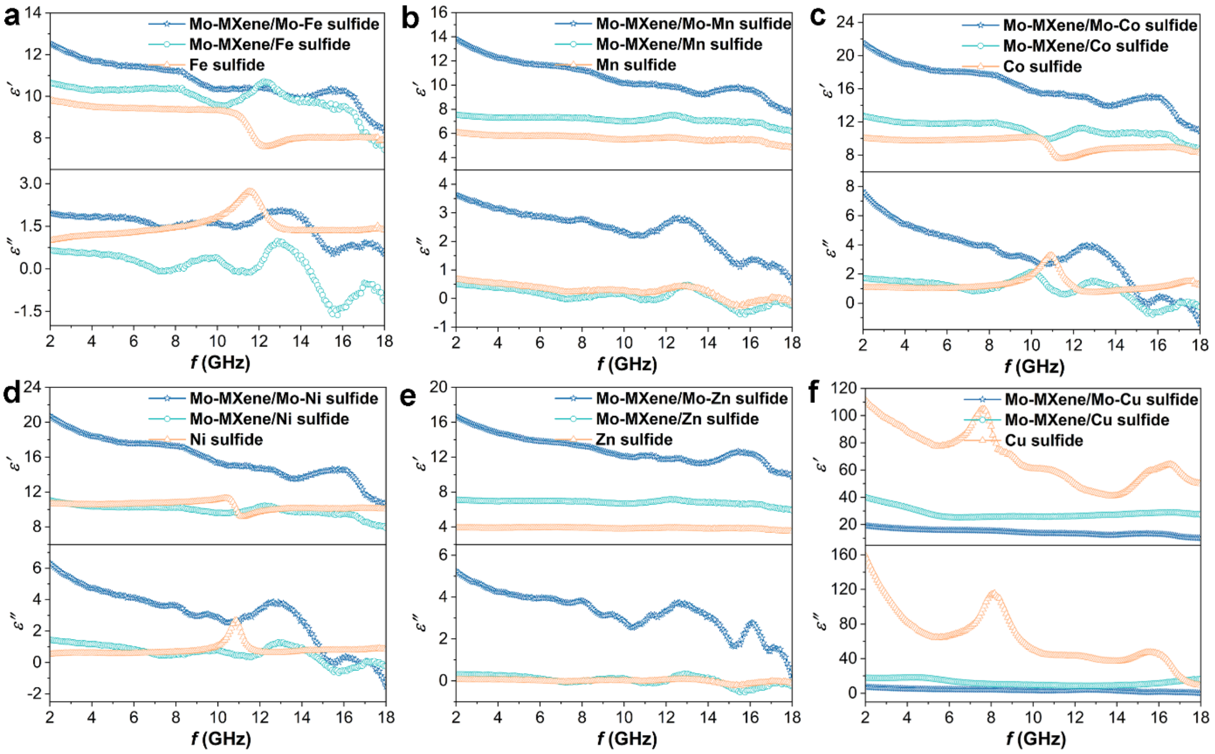

Fig. S37 Permittivity of (**a**) Fe system, (**b**) Mn system, (**c**) Co system, (**d**) Ni system, (**e**) Zn system, and (**f**) Cu system

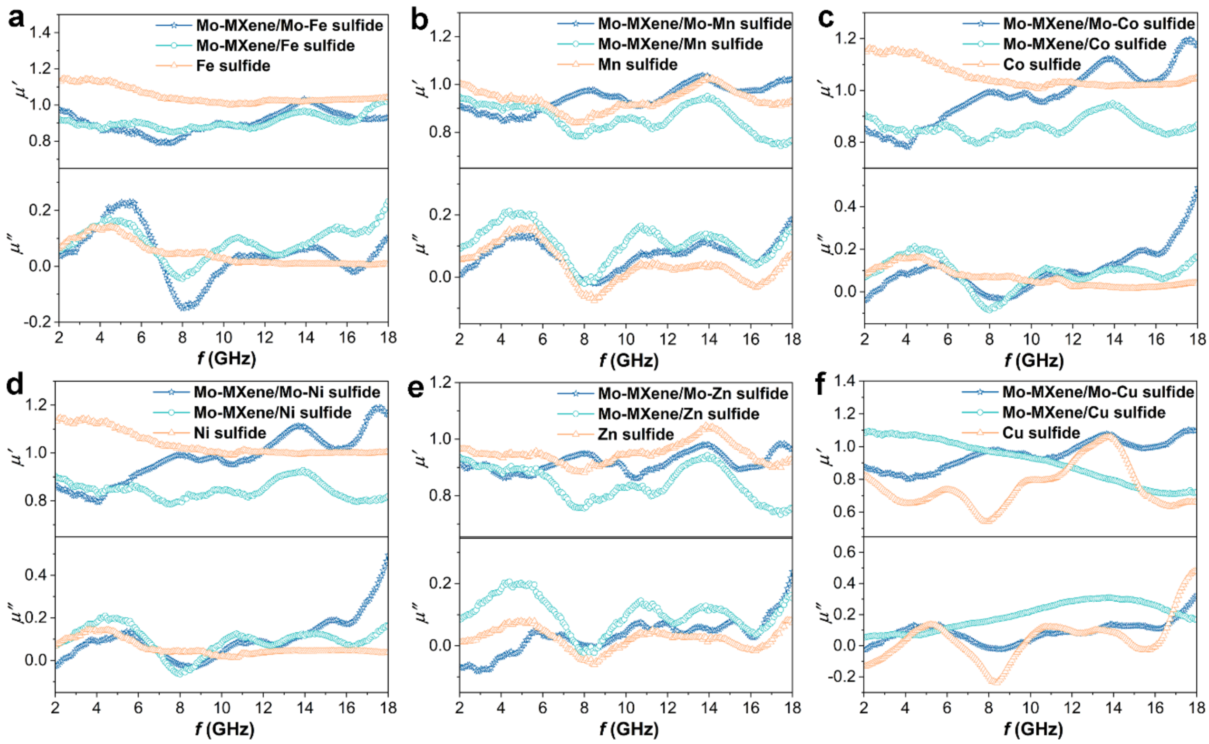

Fig. S38 Permeability of (**a**) Fe system, (**b**) Mn system, (**c**) Co system, (**d**) Ni system, (**e**) Zn system, and (**f**) Cu system

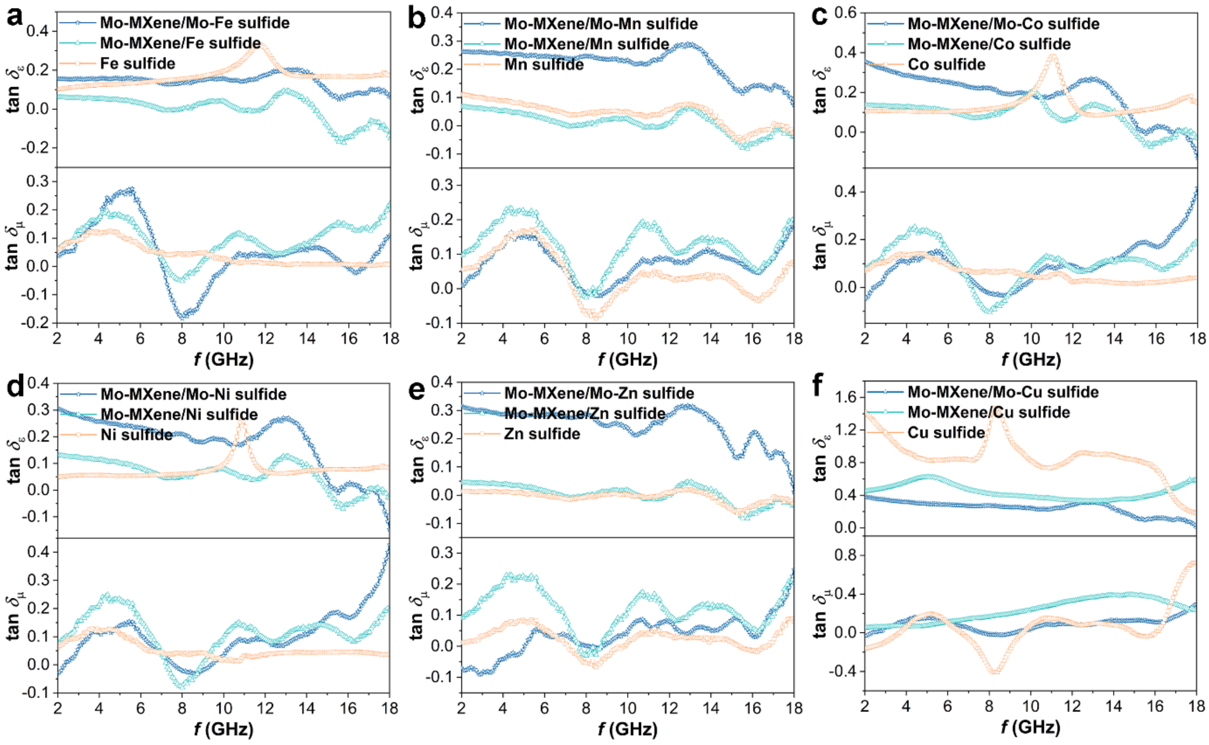

Fig. S39 tan δ_ε and tan δ_μ of (**a**) Fe system, (**b**) Mn system, (**c**) Co system, (**d**) Ni system, (**e**) Zn system, and (**f**) Cu system

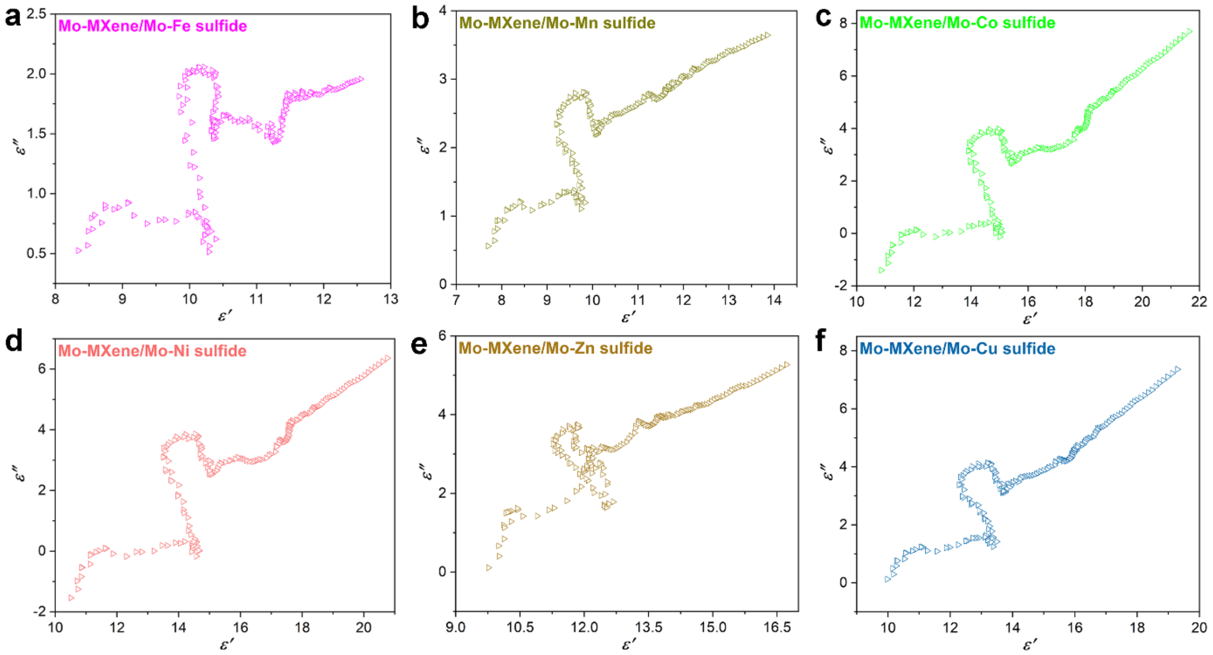

Fig. S40 ε'-ε'' curves of (**a**) Mo-MXene/Mo-Fe sulfide, (**b**) Mo-MXene/Mo-Mn sulfide, (**c**) Mo-MXene/Mo-Co sulfide, (**d**) Mo-MXene/Mo-Ni sulfide, (**e**) Mo-MXene/Mo-Zn sulfide, and (**f**) Mo-MXene/Mo-Cu sulfide

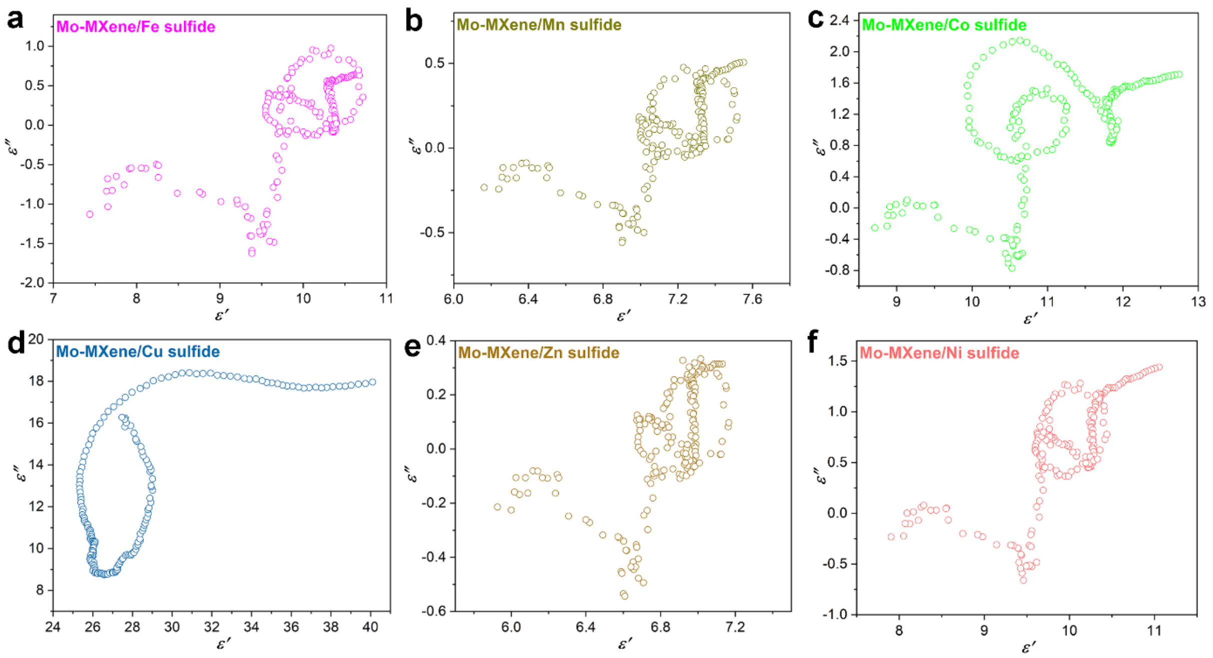

Fig. S41 ε'-ε'' curves of (**a**) Mo-MXene/Fe sulfide, (**b**) Mo-MXene/Mn sulfide, (**c**) Mo-MXene/Co sulfide, (**d**) Mo-MXene/Ni sulfide, (**e**) Mo-MXene/Zn sulfide, and (**f**) Mo-MXene/Cu sulfide

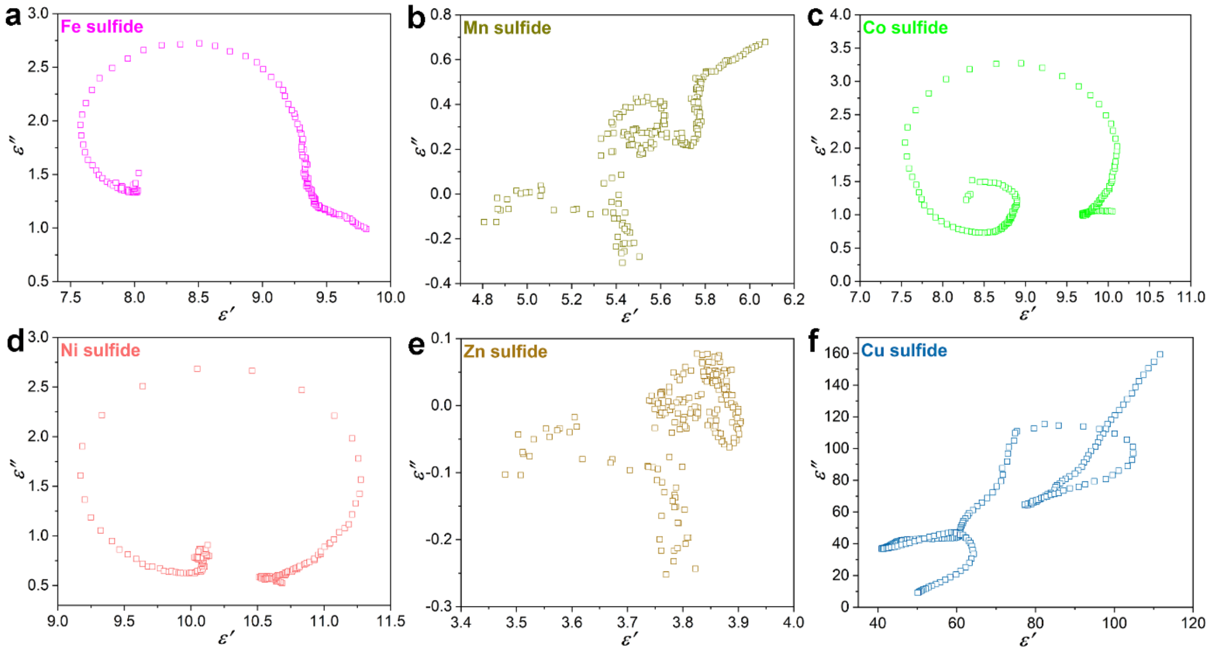

Fig. S42 ε'-ε'' curves of (**a**) Fe sulfide, (**b**) Mn sulfide, (**c**) Co sulfide, (**d**) Ni sulfide, (**e**) Zn sulfide, and (**f**) Cu sulfide

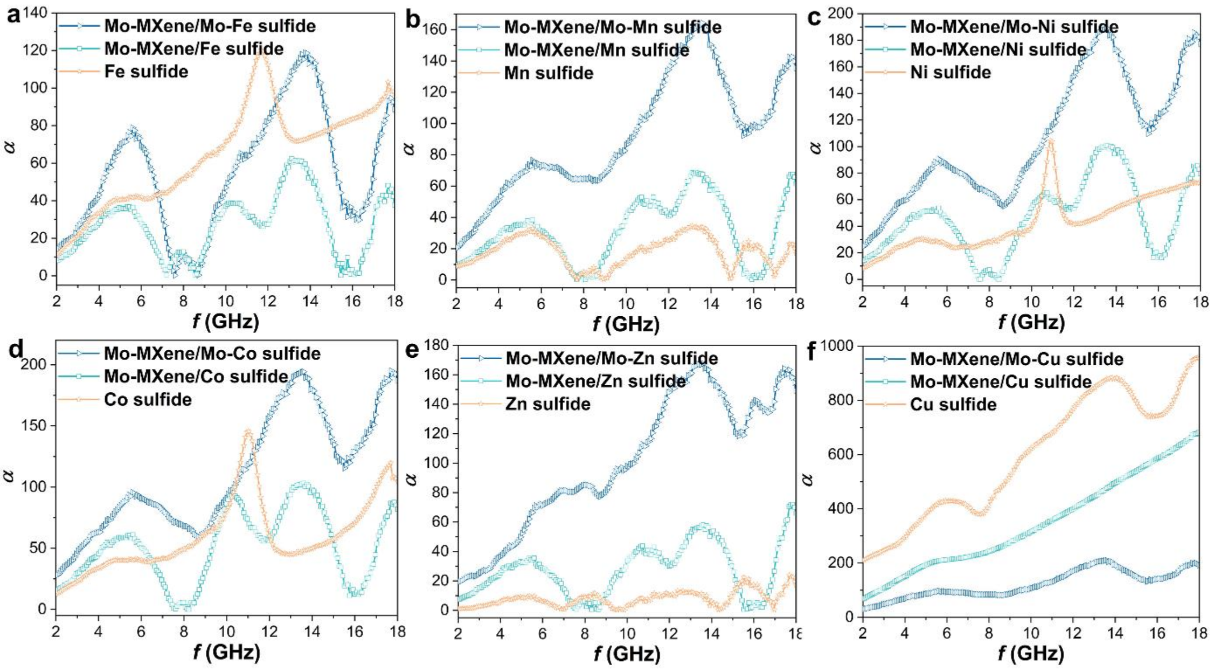

Fig. S43 Frequency dependence of α for (**a**) Fe system, (**b**) Mn system, (**c**) Co system, (d) Ni system, (**e**) Zn system, and (**f**) Cu system

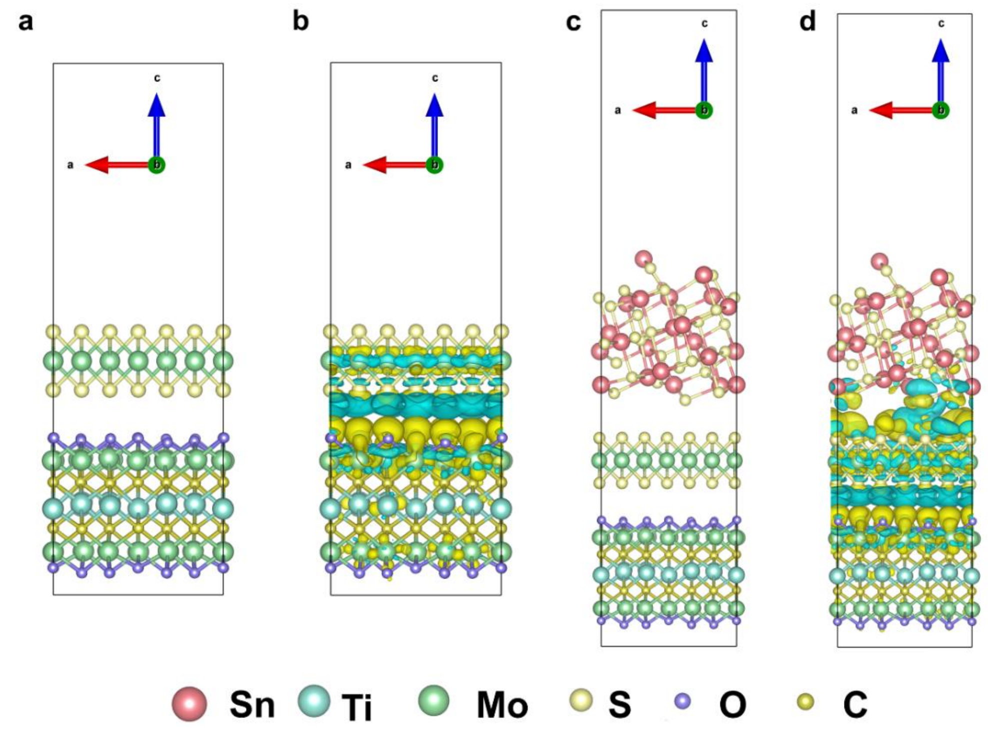

Fig. S44 Differential charge density of the (a,b) Mo-MXene/MoS_2 and (c,d) Mo-MXene/Mo-Sn-sulfide models, where the blue-green region represents electron consumption and the yellow region represents electron accumulation

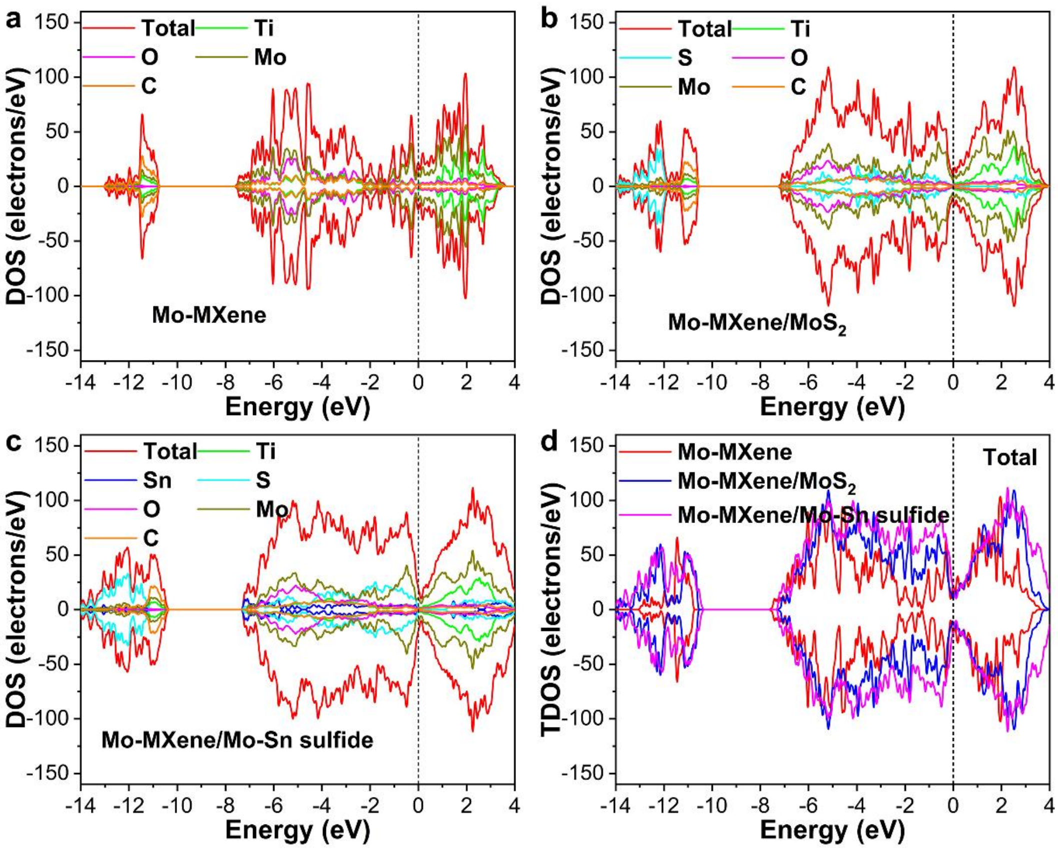

Fig. S45 DOS plots for (**a**) Mo-MXene, (**b**) Mo-MXene/MoS_2, and (**c**) Mo-MXene/Mo-Sn sulfide. (**d**) TDOS plots for different samples

Table S1 EMW absorption performance of Mo-MXene/Mo-Sn sulfide and recently advanced MXene-based absorbers

No.	Materials	Shapes	Thickness /mm	R_L /dB	Bandwidth (< -10 dB) /GHz	R_L/d /dB mm^{-1}	Refs
S1	$Ti_3C_2T_x$@MoS_2@C	Nanosheets	4.80	-20.80	1.00	-4.33	[S8]
S2	$Ti_3C_2T_x$	Layered	4.00	-27.50	3.00	-6.88	[S9]
S3	rGO/MXene/FeS	3D networks	4.78	-47.17	7.85	-9.87	[S10]
S4	FeNi/$Ti_3C_2T_x$	Layered	1.60	-16.96	6.20	-10.60	[S11]
S5	MXene nanoribbons-NiCo@NC	hierarchical network	4.82	-57.10	4.82	-11.85	[S12]
S6	Ti_3C_2	Nanosheets	1.40	-17.00	6.00	-12.14	[S13]
S7	MoO_3/TiO_2/$Mo_2TiC_2T_x$	Layered	2.30	-30.76	8.60	-13.37	[S14]
S8	$NiFe_2O_4$/MXene	Nanosheets	2.90	-41.83	1.60	-14.42	[S15]
S9	MXene/Ni	Layered	3.50	-50.50	5.28	-14.43	[S16]
S10	MXene/PI	Aerogel	3.00	-45.40	3.70	-15.13	[S17]
S11	$Mo_2TiC_2T_x$ MXene	Layered	1.60	-25.39	3.20	-15.87	[S18]
S12	CoO/$NiCo_2O_4$/MXene	Flower-like	2.90	-47.17	5.44	-16.27	[S19]
S13	MXene/C-CNTs	Microspheres	2.70	-45.00	4.90	-16.67	[S20]
S14	MXene@C	Nanosheets	2.80	-46.92	7.01	-16.76	[S21]
S15	MXene/Fe-MOFs	Sheets	3.00	-51.80	6.50	-17.27	[S22]
S16	GO/MXene/Fe_3O_4	Microspheres	2.90	-51.20	4.70	-17.66	[S23]
S17	Co_9S_8/C/$Ti_3C_2T_x$	Flakes	2.51	-50.07	4.24	-19.95	[S24]
S18	Fe/MXene	3D networks	2.00	-40.30	1.40	-20.15	[S25]
S19	$CoFe_2O_4$-Ti_3C_2	Layered	1.50	-30.90	8.50	-20.60	[S26]
S20	SiO_2@MXene@MoS_2	Layered	2.40	-52.11	6.72	-21.71	[S27]
S21	rGO/MXene/TiO_2/Fe_2C	Flowers	3.10	-67.40	5.47	-21.74	[S28]
S22	MXene/FeCo	Film	2.00	-43.70	1.00	-21.85	[S29]
S23	Fe-doped Ti_3AlC_2	Ternary layered	1.50	-33.30	3.90	-22.20	[S30]
S24	NiFe LDH/MXene	Sheet-fiber	2.50	-58.00	7.00	-23.20	[S31]
S25	MXene/MoS_2	Nanosheets	2.00	-46.72	4.32	-23.36	[S32]
S26	$Ti_3C_2T_x$/$NiCo_2O_4$	Nanosheets	2.18	-50.96	0.88	-23.38	[S33]
S27	MXene-CNTs/Ni	Seed-germination-like	2.40	-56.40	2.00	-23.50	[S34]
S28	MXene-CNTs/Ni	Layered	2.40	-56.40	3.95	-23.50	[S34]
S29	rGO/Nb_2CT_x/Fe_3O_4	Layered	2.50	-59.17	6.80	-23.67	[S35]
S30	NiS/MoS_2/$Ti_3C_2T_x$	Layered	2.40	-58.48	5.04	-24.37	[S36]
S31	Co/CNTs-MXene@CF	Nanotubes	2.52	-61.41	5.04	-24.37	[S37]
S32	NiCo/TiC/TiO/CNTs	Nanotubers	2.10	-51.98	7.76	-24.75	[S38]
S33	CNF/MXene	Loofah-like	2.50	-63.80	7.32	-25.52	[S39]
S34	$Ti_3C_2T_x$ MXene	Single-layer	1.70	-43.50	6.88	-25.59	[S40]
S35	M-SiC_{nw}/MXene	Fibers	1.58	-41.70	3.36	-26.39	[S41]
S36	$NiFe_2O_4$@SiO_2@MXene	Layered	2.00	-52.80	7.20	-26.40	[S42]

S37	MXenes/MnO_2/$NiCo_2S_4$	Nanorods	2.17	-59.23	5.80	-27.29	[S43]
S38	MXene/Ni	Flowers	1.90	-52.70	3.90	-27.74	[S44]
S39	$Ti_3C_2T_x$	Foam	1.80	-50.60	4.20	-28.11	[S45]
S40	MXene/RGO/CNCs	Nanosheets	2.56	-72.32	4.96	-28.25	[S46]
S41	MXene/Ni	Chain	1.75	-49.90	2.00	-28.51	[S47]
S42	$Ti_3C_2T_x$@ZnO	Hollow spheres	2.00	-57.40	6.56	-28.70	[S48]
S43	Alk-$Ti_3C_2T_x$	Layered	1.70	-49.10	3.90	-28.88	[S49]
S44	$Ti_3C_2T_x$ MXene	Bowls	1.80	-53.80	4.20	-29.89	[S50]
S45	MXene/Ni	Chains	1.75	-52.47	7.00	-29.98	[S51]
S46	TiO_2/$Ti_3C_2T_x$/Fe_3O_4	Layered	1.90	-57.30	2.00	-30.16	[S52]
S47	Co/ZnO@CMWCNTs/$Ti_3C_2T_x$	Flower-like	1.50	-46.00	4.00	-30.67	[S53]
S48	$Ti_3C_2T_x$/CNFs/TiO_2/CoNi	Layered	1.76	-54.60	4.00	-31.02	[S54]
S49	NiCo-LDH/MXene	Layered	2.00	-64.24	4.48	-32.12	[S55]
S50	MXene/CoNi/C	Fibers	1.60	-51.60	4.50	-32.25	[S56]
-	Mo-MXene/Mo-Sn sulfide	Layered	1.885	-70.60	3.92	-37.45	This work

Note: The exact R_L values, thickness, and bandwidth were not presented in some references, thus, those values were dug out according to the R_L-f curves.

Supplementary References

[S1] G. Kresse, J. Furthmüller Efficiency of ab-initio total energy calculations for metals and semiconductors using a plane-wave basis set. Comput. Mater. Sci. **6**, 15–50 (1996). https://doi.org/10.1016/0927-0256(96)00008-0

[S2] G. Kresse, J. Furthmüller Efficient iterative schemes for *ab initio* total-energy calculations using a plane-wave basis set. Phys. Rev. B Condens. Matter **54**, 11169–11186 (1996). https://doi.org/10.1103/physrevb.54.11169

[S3] J.P. Perdew, K. Burke, M. Ernzerhof Generalized gradient approximation made simple. Phys. Rev. Lett. **77**, 3865–3868 (1996). https://doi.org/10.1103/physrevlett.77.3865

[S4] G. Kresse, D. Joubert From ultrasoft pseudopotentials to the projector augmented-wave method. Phys. Rev. B **59**, 1758–1775 (1999). https://doi.org/10.1103/physrevb.59.1758

[S5] P.E. Blöchl Projector augmented-wave method. Phys. Rev. B **50**, 17953–17979 (1994). https://doi.org/10.1103/physrevb.50.17953

[S6] S. Grimme, J. Antony, S. Ehrlich, H. Krieg A consistent and accurate *ab initio* parametrization of density functional dispersion correction (DFT-D) for the 94 elements H-Pu. J. Chem. Phys. **132**, 154104 (2010). https://doi.org/10.1063/1.3382344

[S7] S. Grimme, S. Ehrlich, L. Goerigk Effect of the damping function in dispersion corrected density functional theory. J. Comput. Chem. **32**, 1456–1465 (2011). https://doi.org/10.1002/jcc.21759

[S8] X. Zhou, H. Han, H. Yan, Y. Wang, C. Zhang et al., Multi-interface self-assembling on MXenes skeleton towards wideband electromagnetic dissipation. Mater. Today Phys. **24**, 100685 (2022). https://doi.org/10.1016/j.mtphys.2022.100685

[S9] G. Cui, X. Sun, G. Zhang, Z. Zhang, H. Liu et al., Electromagnetic absorption performance of two-dimensional MXene Ti3C2Tx exfoliated by HCl + LiF etchant with diverse etching times. Mater. Lett. **252**, 8–10 (2019). https://doi.org/10.1016/j.matlet.2019.05.053

[S10] S. Li, X. Tang, X. Zhao, S. Lu, J. Luo et al., Hierarchical graphene@MXene composite foam modified with flower-shaped FeS for efficient and broadband electromagnetic absorption. J. Mater. Sci. Technol. **133**, 238–248 (2023). https://doi.org/10.1016/j.jmst.2022.06.018

[S11] J. He, X. Liu, Y. Deng, Y. Peng, L. Deng et al., Improved magnetic loss and impedance matching of the FeNi-decorated $Ti_3C_2T_x$ MXene composite toward the broadband microwave absorption performance. J. Alloys Compd. **862**, 158684 (2021). https://doi.org/10.1016/j.jallcom.2021.158684

[S12] X. Zeng, C. Zhao, T. Nie, Z.-Y. Shen, R. Yu et al., Construction of 0D/1D/2D MXene nanoribbons-NiCo@NC hierarchical network and their coupling effect on electromagnetic wave absorption. Mater. Today Phys. **28**, 100888 (2022). https://doi.org/10.1016/j.mtphys.2022.100888

[S13] Y. Qing, W. Zhou, F. Luo, D. Zhu Titanium carbide (MXene) nanosheets as promising microwave absorbers. Ceram. Int. **42**, 16412–16416 (2016). https://doi.org/10.1016/j.ceramint.2016.07.150

[S14] F. Hu, F. Zhang, X. Wang, Y. Li, H. Wang et al., Ultrabroad band microwave absorption from hierarchical $MoO_3/TiO_2/Mo_2TiC_2$Tx hybrids via annealing treatment. J. Adv. Ceram. **11**, 1466–1478 (2022). https://doi.org/10.1007/s40145-022-0624-0

[S15] Y. Guo, D. Wang, T. Bai, H. Liu, Y. Zheng et al., Electrostatic self-assembled $NiFe_2O_4$/Ti3C2Tx MXene nanocomposites for efficient electromagnetic wave absorption at ultralow loading level. Adv. Compos. Hybrid Mater. **4**, 602–613 (2021). https://doi.org/10.1007/s42114-021-00279-0

[S16] X. Li, W. You, L. Wang, J. Liu, Z. Wu et al., Self-assembly-magnetized MXene avoid dual-agglomeration with enhanced interfaces for strong microwave absorption through a tunable electromagnetic property. ACS Appl. Mater. Interfaces **11**, 44536–44544 (2019). https://doi.org/10.1021/acsami.9b11861

[S17] J. Liu, H.-B. Zhang, X. Xie, R. Yang, Z. Liu et al., Multifunctional, superelastic, and lightweight MXene/polyimide aerogels. Small **14**, e1802479 (2018). https://doi.org/10.1002/smll.201802479

[S18] F. Hu, X. Wang, H. Niu, S. Zhang, B. Fan et al., Synthesis and electromagnetic wave absorption of novel Mo_2TiC_2Tx MXene with diverse etching methods. J. Mater. Sci. **57**, 7849–7862 (2022). https://doi.org/10.1007/s10853-022-07202-y

[S19] H.-Y. Wang, X.-B. Sun, G.-S. Wang A MXene-modulated 3D crosslinking network of hierarchical flower-like MOF derivatives towards ultra-efficient microwave absorption properties. J. Mater. Chem. A **9**, 24571–24581 (2021). https://doi.org/10.1039/D1TA06505J

[S20] Y. Cui, F. Wu, J. Wang, Y. Wang, T. Shah et al., Three dimensional porous MXene/CNTs microspheres: Preparation, characterization and microwave absorbing

properties. Compos. Part A Appl. Sci. Manuf. **145**, 106378 (2021). https://doi.org/10.1016/j.compositesa.2021.106378

[S21] W. Huyan, J. Yao, J. Tao, F. Yang, X. Tao et al., MXene@C heterogeneous nanocomposites with the 2D-0D structure for ultra-light and broadband electromagnetic wave absorption. Carbon **197**, 444–454 (2022). https://doi.org/10.1016/j.carbon.2022.06.070

[S22] B. Deng, Z. Xiang, J. Xiong, Z. Liu, L. Yu et al., Sandwich-like Fe&TiO_2@C nanocomposites derived from MXene/Fe-MOFs hybrids for electromagnetic absorption. Nano-Micro Lett. **12**, 55 (2020). https://doi.org/10.1007/s40820-020-0398-2

[S23] Y. Cui, K. Yang, J. Wang, T. Shah, Q. Zhang et al., Preparation of pleated RGO/MXene/Fe_3O_4 microsphere and its absorption properties for electromagnetic wave. Carbon **172**, 1–14 (2021). https://doi.org/10.1016/j.carbon.2020.09.093

[S24] T. Hou, Z. Jia, B. Wang, H. Li, X. Liu et al., MXene-based accordion 2D hybrid structure with Co9S8/C/$Ti_3C_2T_x$ as efficient electromagnetic wave absorber. Chem. Eng. J. **414**, 128875 (2021). https://doi.org/10.1016/j.cej.2021.128875

[S25] J. Liu, W. Yu, Z. Zhao, D. Liu, S. Liu et al., 3D honeycomb Fe/MXene derived from Prussian blue microcubes with a tunable structure for efficient low-frequency and flexible electromagnetic absorbers. ACS Appl. Mater. Interfaces **15**, 48519–48528 (2023). https://doi.org/10.1021/acsami.3c09799

[S26] J. He, S. Liu, L. Deng, D. Shan, C. Cao et al., Tunable electromagnetic and enhanced microwave absorption properties in $CoFe_2O_4$ decorated Ti_3C_2 MXene composites. Appl. Surf. Sci. **504**, 144210 (2020). https://doi.org/10.1016/j.apsusc.2019.144210

[S27] X. Jiang, Q. Wang, L. Song, H. Lu, H. Xu et al., Enhancing electromagnetic wave absorption with core-shell structured SiO_2@MXene@MoS_2 nanospheres. Carbon Energy, e502 (2024). https://doi.org/10.1002/cey2.502

[S28] G. Wang, C. Li, D. Estevez, P. Xu, M. Peng et al., Boosting interfacial polarization through heterointerface engineering in MXene/graphene intercalated-based microspheres for electromagnetic wave absorption. Nano-Micro Lett. **15**, 152 (2023). https://doi.org/10.1007/s40820-023-01123-4

[S29] X. Li, C. Wen, L. Yang, R. Zhang, X. Li et al., MXene/FeCo films with distinct and tunable electromagnetic wave absorption by morphology control and magnetic anisotropy. Carbon **175**, 509–518 (2021). https://doi.org/10.1016/j.carbon.2020.11.089

[S30] J. Li, T. Xu, H. Bai, Z. Shen, Y. Huang et al., Structural modifications and electromagnetic property regulations of Ti_3AlC_2 MAX for enhancing microwave absorption through the strategy of Fe doping. Adv. Mater. Interfaces **9**, 2101510 (2022). https://doi.org/10.1002/admi.202101510

[S31] Z. Wang, L. Yang, Y. Zhou, C. Xu, M. Yan et al., NiFe LDH/MXene derivatives interconnected with carbon fabric for flexible electromagnetic wave absorption. ACS Appl. Mater. Interfaces **13**, 16713–16721 (2021). https://doi.org/10.1021/acsami.1c05007

[S32] X. Li, C. Wen, L. Yang, R. Zhang, Y. Li et al., Enhanced visualizing charge distribution of 2D/2D MXene/MoS_2 heterostructure for excellent microwave absorption performance. J. Alloys Compd. **869**, 159365 (2021). https://doi.org/10.1016/j.jallcom.2021.159365

[S33] T. Hou, B. Wang, M. Ma, A. Feng, Z. Huang et al., Preparation of two-dimensional titanium carbide ($Ti_3C_2T_x$) and $NiCo_2O_4$ composites to achieve excellent microwave absorption properties. Compos. Part B Eng. **180**, 107577 (2020). https://doi.org/10.1016/j.compositesb.2019.107577

[S34] X. Li, W. You, C. Xu, L. Wang, L. Yang et al., 3D seed-germination-like MXene with *in situ* growing CNTs/Ni heterojunction for enhanced microwave absorption via polarization and magnetization. Nano-Micro Lett. **13**, 157 (2021). https://doi.org/10.1007/s40820-021-00680-w

[S35] C. Cui, R. Guo, E. Ren, H. Xiao, M. Zhou et al., MXene-based rGO/Nb_2CT_x/Fe_3O_4 composite for high absorption of electromagnetic wave. Chem. Eng. J. **405**, 126626 (2021). https://doi.org/10.1016/j.cej.2020.126626

[S36] M. Chang, Z. Jia, S. He, J. Zhou, S. Zhang et al., Two-dimensional interface engineering of NiS/MoS_2/$Ti_3C_2T_x$ heterostructures for promoting electromagnetic wave absorption capability. Compos. Part B Eng. **225**, 109306 (2021). https://doi.org/10.1016/j.compositesb.2021.109306

[S37] B. Wang, S. Li, F. Huang, S. Wang, H. Zhang et al., Construction of multiple electron transfer paths in 1D core-shell hetetrostructures with MXene as interlayer enabling efficient microwave absorption. Carbon **187**, 56–66 (2022). https://doi.org/10.1016/j.carbon.2021.10.080

[S38] T. Hou, Z. Jia, Y. Dong, X. Liu, G. Wu Layered 3D structure derived from MXene/magnetic carbon nanotubes for ultra-broadband electromagnetic wave absorption. Chem. Eng. J. **431**, 133919 (2022). https://doi.org/10.1016/j.cej.2021.133919

[S39] H. Peng, M. He, Y. Zhou, Z. Song, Y. Wang et al., Low-temperature carbonized biomimetic cellulose nanofiber/MXene composite membrane with excellent microwave absorption performance and tunable absorption bands. Chem. Eng. J. **433**, 133269 (2022). https://doi.org/10.1016/j.cej.2021.133269

[S40] X. Zhou, J. Wen, Z. Wang, X. Ma, H. Wu Broadband high-performance microwave absorption of the single-layer $Ti_3C_2T_x$ MXene. J. Mater. Sci. Technol. **115**, 148–155 (2022). https://doi.org/10.1016/j.jmst.2021.11.029

[S41] Y. Wang, Q. Dou, W. Jiang, K. Su, J. You et al., $Ti_3C_2T_X$MXene beaded SiC nanowires for efficient microwave absorption. ACS Appl. Nano Mater. **5**, 9209–9222 (2022). https://doi.org/10.1021/acsanm.2c01553

[S42] M. Yuan, M. Zhou, H. Fu Synergistic microstructure of sandwich-like $NiFe_2O_4$@SiO_2@MXene nanocomposites for enhancement of microwave absorption in the whole Ku-band. Compos. Part B Eng. **224**, 109178 (2021). https://doi.org/10.1016/j.compositesb.2021.109178

[S43] F. Pan, X. Wu, D. Batalu, W. Lu, H. Guan Assembling of low-dimensional aggregates with interlaminar electromagnetic synergy network for high-efficient microwave absorption. Adv. Powder Mater. **2**, 100100 (2023). https://doi.org/10.1016/j.apmate.2022.100100

[S44] Y. Cui, Z. Liu, Y. Zhang, P. Liu, M. Ahmad et al., Wrinkled three-dimensional porous MXene/Ni composite microspheres for efficient broadband microwave absorption. Carbon **181**, 58–68 (2021). https://doi.org/10.1016/j.carbon.2021.05.022

[S45] K. Hu, H. Wang, X. Zhang, H. Huang, T. Qiu et al., Ultralight $Ti_3C_2T_x$ MXene foam with superior microwave absorption performance. Chem. Eng. J. **408**, 127283 (2021). https://doi.org/10.1016/j.cej.2020.127283

[S46] L. Zhu, Y. Cao, B. Jiang, J. Yu, J. Zhu et al., Compressible, elastic and 3D porous $Ti_3C_2T_x$ MXene/RGO/CNCs composite aerogel for electromagnetic wave absorbing. J. Alloys Compd. **969**, 172117 (2023). https://doi.org/10.1016/j.jallcom.2023.172117

[S47] L. Liang, G. Han, Y. Li, B. Zhao, B. Zhou et al., Promising $Ti_3C_2T_x$ MXene/Ni chain hybrid with excellent electromagnetic wave absorption and shielding capacity. ACS Appl. Mater. Interfaces **11**, 25399–25409 (2019). https://doi.org/10.1021/acsami.9b07294

[S48] Y.-Q. Wang, H.-B. Zhao, J.-B. Cheng, B.-W. Liu, Q. Fu et al., Hierarchical $Ti_3C_2T_x$@ZnO hollow spheres with excellent microwave absorption inspired by the visual phenomenon of eyeless urchins. Nano-Micro Lett. **14**, 76 (2022). https://doi.org/10.1007/s40820-022-00817-5

[S49] Y. Du, Z. Yan, W. You, Q. Men, G. Chen et al., Balancing MXene surface termination and interlayer spacing enables superior microwave absorption. Adv. Funct. Mater. **33**, 2301449 (2023). https://doi.org/10.1002/adfm.202301449

[S50] Q. Du, Q. Men, R. Li, Y. Cheng, B. Zhao et al., Electrostatic adsorption enables layer stacking thickness-dependent hollow Ti3C2Tx MXene bowls for superior electromagnetic wave absorption. Small ***18**, 2203609 (2022).* https://doi.org/10.1002/smll.202203609

[S51] F. Pan, Y. Rao, D. Batalu, L. Cai, Y. Dong et al., Macroscopic electromagnetic cooperative network-enhanced MXene/Ni chains aerogel-based microwave absorber with ultra-low matching thickness. Nano-Micro Lett. **14**, 140 (2022). https://doi.org/10.1007/s40820-022-00869-7

[S52] P. Liu, Z. Yao, V.M.H. Ng, J. Zhou, L.B. Kong et al., Facile synthesis of ultrasmall Fe_3O_4 nanoparticles on MXenes for high microwave absorption performance. Compos. Part A Appl. Sci. Manuf. **115**, 371–382 (2018). https://doi.org/10.1016/j.compositesa.2018.10.014

[S53] C. Sun, Q. Li, Z. Jia, G. Wu, P. Yin Hierarchically flower-like structure assembled with porous nanosheet-supported MXene for ultrathin electromagnetic wave absorption. Chem. Eng. J. **454**, 140277 (2023). https://doi.org/10.1016/j.cej.2022.140277

[S54] Z. Liu, J. Chen, M. Que, H. Zheng, L. Yang et al., 2D $Ti_3C_2T_x$ MXene/MOFs composites derived CoNi bimetallic nanoparticles for enhanced microwave absorption. Chem. Eng. J. **450**, 138442 (2022). https://doi.org/10.1016/j.cej.2022.138442

[S55] X. Gao, Z. Jia, B. Wang, X. Wu, T. Sun et al., Synthesis of NiCo-LDH/MXene hybrids with abundant heterojunction surfaces as a lightweight electromagnetic wave absorber. Chem. Eng. J. **419**, 130019 (2021). https://doi.org/10.1016/j.cej.2021.130019

[S56] F. Wu, Z. Liu, J. Wang, T. Shah, P. Liu et al., Template-free self-assembly of MXene and CoNi-bimetal MOF into intertwined one-dimensional heterostructure and its microwave absorbing properties. Chem. Eng. J. **422**, 130591 (2021). https://doi.org/10.1016/j.cej.2021.130591
